# Identification of ferroptosis-related genes in syncytiotrophoblast-derived extracellular vesicles of preeclampsia

**DOI:** 10.1097/MD.0000000000031583

**Published:** 2022-11-04

**Authors:** Quanfeng Wu, Xiang Ying, Weiwei Yu, Huanxi Li, Wei Wei, Xueyan Lin, Xueqin Zhang

**Affiliations:** a Department of Obstetrics, Women and Children’s Hospital, School of Medicine, Xiamen University, Xiamen, China; b Department of Gynecology and Obstetrics, Shanghai Jiaotong University School of Medicine Xinhua Hospital, Shanghai, China.

**Keywords:** bioinformatical analysis, extracellular vesicles, ferroptosis, hub gene, preeclampsia

## Abstract

Preeclampsia (PE), defined as new-onset hypertension and multi-organ systemic complication during pregnancy, is the leading cause of maternal and neonatal mortality and morbidity. With extracellular vesicles research progresses, current data refers to the possibility that ferroptosis may play a role in exosomal effects. Evidence has suggested that ferroptosis may contribute to the pathogenesis of preeclampsia by bioinformatics analyses. The purpose of the current study is to identify the potential ferroptosis-related genes in syncytiotrophoblast-derived extracellular vesicles (STB-EVs) of preeclampsia using bioinformatics analyses. Clinical characteristics and gene expression data of all samples were obtained from the NCBI GEO database. The differentially expressed mRNAs (DE-mRNAs) in STB-EVs of preeclampsia were screened and then were intersected with ferroptosis genes. Functional and pathway enrichment analyses of ferroptosis-related DE-mRNAs in STB-EVs were performed. Ferroptosis-related hub genes in STB-EVs were identified by Cytoscape plugin CytoHubba with a Degree algorithm using a protein-protein interaction network built constructed from the STRING database. The predictive performance of ferroptosis-related hub genes was determined by a univariate analysis of receiver operating characteristic (ROC). The miRNA-hub gene regulatory network was constructed using the miRwalk database. A total of 1976 DE-mRNAs in STB-EVs were identified and the most enriched item identified by gene set enrichment analysis was signaling by G Protein-Coupled Receptors (normalized enrichment score = 1.238). These DE-mRNAs obtained 26 ferroptosis-related DE-mRNAs. Ferroptosis-related DE-mRNAs of gene ontology terms and Encyclopedia of Genes and Genomes pathway enrichment analysis were enriched significantly in response to oxidative stress and ferroptosis. Five hub genes (ALB, NOX4, CDKN2A, TXNRD1, and CAV1) were found in the constructed protein-protein interaction network with ferroptosis-related DE-mRNAs and the areas under the ROC curves for ALB, NOX4, CDKN2A, TXNRD1, and CAV1 were 0.938 (CI: 0.815−1.000), 0.833 (CI: 0.612−1.000), 0.875 (CI: 0.704−1.000), 0.958 (CI: 0.862−1.000), and 0.854 (CI: 0.652−1.000) in univariate analysis of ROC. We constructed a regulatory network of miRNA-hub gene and the findings demonstrate that hsa-miR-26b-5p, hsa-miR-192-5p, hsa-miR-124-3p, hsa-miR-492, hsa-miR-34a-5p and hsa-miR-155-5p could regulate most hub genes. In this study, we identified several central genes closely related to ferroptosis in STB-EVs (ALB, NOX4, CDKN2A, TXNRD1, and CAV1) that are potential biomarkers related to ferroptosis in preeclampsia. Our findings will provide evidence for the involvement of ferroptosis in preeclampsia and improve the understanding of ferroptosis-related molecular pathways in the pathogenesis of preeclampsia.

## 1. Introduction

Preeclampsia (PE), a multiorgan systemic disorder related to placental pathology, is the major risk factor for long-term cardiovascular disease in mothers.^[1]^ PE is the leading primary cause of maternal and neonatal mortality and morbidity, affecting 2% to 8% of pregnancies and leading to over 46,000 maternal deaths and 500,000 fetal deaths globally each year.^[2,3]^ PE is considered to develop as a result of inadequate placentation and will eventually disappear after the placenta removed. However, the fundamental processes by which the dysfunctional placenta affects the development of preeclampsia is not yet known.^[4]^ The placenta, an endocrine organ during pregnancy, coordinates the maternal adaptive response to pregnancy and extracellular vesicles (EVs) derived from placenta can contribute to the development of preeclampsia.^[5]^ EVs are about 0.1 to 1.0 m in diameter and contain lot of bioactive carriers (protein, lipids, nucleic acids, etc)^[6]^ and participate in physiological and pathological processes such as gene regulation, immune regulation, maternal metabolism and maintenance of pregnancy.^[7]^ During pregnancy, syncytiotrophoblast (STB) cells of placenta continuously release EVs into maternal circulation and the concentration of STB-EVs in maternal peripheral blood will increase due to pathological pregnancy.^[8,9]^ STB-EVs may contribute to preeclampsia by expressing angiogenic and anti-angiogenic factors on its membrane.^[10,11]^ The roles of STB-EVs in normal and pathologic pregnancy are only beginning to be understood and appreciated. Therefore, a thorough knowledge of STB-EVs dysfunction at the molecular level would be beneficial for the treatment and early diagnosis of preeclampsia.

Ferroptosis is a recently discovered type of iron-dependent cell death and is characterized by labile iron overload, an accumulation of iron-dependent lipid peroxidation products and a redox imbalance.^[12]^ Ferroptosis, together with netosis, necroptosis, apoptosis, pyroptosis and entosis, establishes a sequence of various modes of programmed cell death to regulate a variety of physiological and pathological processes.^[13]^ Ferroptosis has received a lot of interest in recent years and has been thoroughly researched in numerous pathogenic processes. By focusing on ferroptosis, novel medicines and disease prevention strategies can be developed. Evidence suggests that ferroptosis may be involved in the pathophysiology of preeclampsia.^[14]^ As a byproduct of lipid peroxidation and a hallmark of ferroptosis, elevated levels of MDA have been found in preeclamptic sera compared to normal pregnancy in several studies and MDA levels were positively correlated with the severity of the disease and negatively correlated with ferroptosis inhibitor concentrations.^[15,16]^ Several investigations suggested that GPX4 (the loss of antioxidant production and turnover, a hallmark of ferroptosis) played a crucial role in preeclamptic pregnancies. Imai et al^[17]^ demonstrating that GPX4-knockout mice embryos could not survive through day 8.5. GSH levels, GPX4 activity, and GPX4 expression are lower in preeclampsia pregnancies compared to normal pregnancy, and these variations are correlated with the severity of the disease.^[15,16,18,19]^ More and more researches focus on the treatment and diagnosis strategies based on the death effect of exocrine iron. Several studies showed that two key genes involved in regulating intracellular iron transport are down regulated by exosomes: bivalent metal transporter 1^[20]^ and iron regulatory protein 2.^[21]^ Exosomes regulate the expression of GPX4 and FSP1,thereby inhibiting ferroptosis.^[22,23]^ However, further improvements in preeclampsia therapeutic interventions that target ferroptosis demand a deep understanding of ferroptosis-related gene expression patterns in the STB-EVs. Therefore, the molecular mechanisms leading to preeclampsia from the perspective of ferroptosis have substantial clinical value for the treatment and early diagnosis.

The ferroptosis-related genes in STB-EVs of preeclampsia have not yet been the focus of bioinformatic investigations. In this study, we employed a data mining and data analysis approach to investigate the gene-expression profile of STB-EVs identified in preeclampsia and normal pregnancy. Gene ontology (GO) annotation, Kyoto Encyclopedia of Genes and Genomes (KEGG) pathway enrichment analysis, correlation analysis of the ferroptosis-related differentially expressed mRNAs (DE-mRNAs) in STB-EVs of preeclampsia, and establishment of a protein-protein interaction (PPI) network were performed. Then, ferroptosis-related hub genes were identified. Transcription factors and microRNAs were then predicted. Overall, this study will improve the knowledge of STEB-EVs caused by ferroptosis in preeclampsia at the molecular level and offer fresh insights into the early clinical diagnosis and treatment of preeclampsia.

## 2. Method

### 2.1. Microarray data acquisition and processing

**Figure 1.**
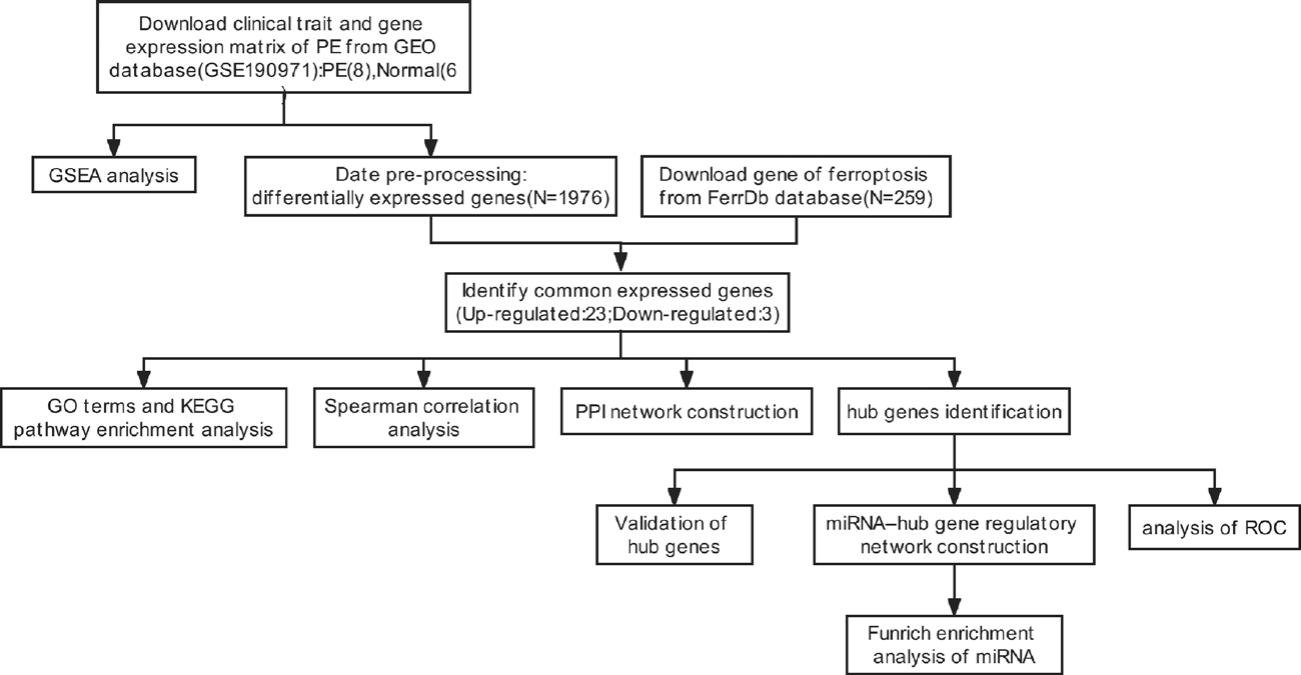
Schematic presentation of the analysis process.

To find the gene expression datasets, we searched the GEO database using the keywords “(preeclampsia) AND “extracellular vesicles or exosome” and “Expression profiling by array” Two GEO datasets (GSE166846, GSE190971) were found following the thorough review. Due to the limited number of samples and differentially expressed genes found in GSE166846, this GSE profiles were excluded. The mRNA expression profiling data of STB-EVs from GSE190971, which was based on GPL11154 Illumina HiSeq 2000 (Homo sapiens), was selected and downloaded. The array data for GSE190971 included 8 STB-EVs of preeclampsia and 6 STB-EVs of normal pregnancy. The characteristics of the patients in the two groups are presented in Table 1. The flowchart for data processing is shown in Figure 1. Our study used open-source data, NCBI’s GEO public database, and the patients who have access to it have ethical approval. There are no ethical problems or other potential conflicts of interest. Additionally, the principal component analysis plot of 14 samples was constructed using the “ggplot2” package (version 3.3.3) in R (version 4.2.1).

**Table 1:** Basic characteristics of the included population.

Variables	PE (n = 8)	Normal (n = 6)	*P*
Maternal age (yr)	33.38 ± 4.17	34.67 ± 4.5	.589
Maternal weight (kg)	84.75 ± 25.15	76.00 ± 20.58	.501
Maternal height (cm)	164.00 ± 6.69	165.17 ± 6.61	.751
BMI (kg/m^2^)	31.38 ± 9.47	27.67 ± 7.11	.439
Parity, n			
Nulliparous	4	0	.04
Multiparous	4	6	
Gravidity, n			
1–2	6	3	.124
>2	1	3	
Gestational age at diagnosis (wk)	30.63 ± 4.43	N/A	N/A
Gestational age at delivery (wk)	32.25 ± 3.57	39.33 ± 1.03	.001
Maximum systolic blood pressure (mm Hg)	167.63 ± 19.34	124.33 ± 13.60	.001
Maximum diastolic blood pressure (mm Hg)	107.13 ± 10.10	69.50 ± 7.12	.000
Urine dipstick, n			
Negative	0	8	.01
1+	2	0	
2+	2	0	
3+	2	0	
4+	2	0	
Birth weight (g)	1686.25 ± 825.43	3755.83 ± 447.48	.000
Infant sex, n			
Male	4	4	.533
Female	4	2	

BMI = body mass index, N/A = not applicable.

### 2.2. Identification of DE-mRNAs in STB-EVs

Differential expression analysis was performed using the “DESeq2” package in R. To acquire more differentially expressed genes (DEGs), the threshold was set at |log fold change| >1.0 and *P*-value < .05. Using a visual hierarchical cluster analysis in the “ggplot2” package (version 3.3.3), DE-mRNAs volcano plots were created.

### 2.3. Gene set enrichment analysis

The C2(CP) gene sets (C2.cp.all.v7.5.1.symbols.gmt) from the Molecular Signatures Database (MSigDB) of the “clusterProfiler” package (version 4.4.4) were used to perform gene set enrichment analysis (GSEA) on the gene expression data in GSE190971.The following criteria were used to evaluate significantly enriched items: false discovery rate (FDR) (*q*value) < 0.25 and *P*.adj < .05.

### 2.4. Ferroptosis-related DEGs

The 259 ferroptosis-related genes were downloaded from Ferroptosis Database(https://www.zhounan.org/ferrdb/legacy/index.html)^[24]^ and a Venn diagram was used to find ferroptosis-related DE-mRNAs by intersecting 259 ferroptosis-related genes with DE-mRNAs in GSE190971. Visual hierarchical cluster analysis in the “ggplot2” package (version 3.3.3) showed ferroptosis-related DE-mRNAs volcano plot. The “complexheatmap” package (version 2.13.1) in R was utilized to construct a heat map of ferroptosis-related DE-mRNAs.

### 2.5. GO terms and KEGG pathway enrichment analysis

To obtain function and involved biological processes of ferroptosis-related DE-mRNAs, Gene Ontology (GO) and Kyoto Encyclopedia of Genes and Genomes (KEGG) annotation was performed using the “clusterprofile” (version 4.4.4), “GOplot” (version 1.0.2) and “org.Hs.e.g..db” (version 3.10.0) packages in R. Visualize enrichment analysis results with the “ggplot2” package (version 3.3.3). The GO analysis included three categories: biological process, cellular component, and molecular function and the KEGG analysis included pathway enrichment analysis. The items with the following criteria were regarded as significantly enriched: *q*value < 0.2 and *P*.adj < .05.

### 2.6. PPI network construction and top 5 hub genes identification

A functional protein association network of ferroptosis-related DE-mRNAs was established using the Search Tool for the Retrieval of Interacting Genes (STRING). After uploading Ferroptosis-related DE-mRNAs to the STRING database, PPI pairs with a combined score of more than 0.4 were retrieved. Furthermore, we find the top 5 hub genes by degree method using the plug-in CytoHubba app in the Cytoscape software (version 3.7.2).^[25]^

### 2.7. miRNa prediction of hub genes and construction of miRNA–hub gene regulatory network

The network-based visual analytics tool Network analyst (http://www.networkanalyst.ca/faces/home.xhtml) is a complete web-based tool for profiling, meta-analysis, and interpretation of gene expression. The gene-miRNA interaction of hub genes was predicted from comprehensive experimentally validated miRNA-gene interaction data base on miRtarbase v8.0 by the web site. Using the Cytoscape software, a miRNA-hub gene regulatory network was constructed.

### 2.8. Enrichment analysis and prediction of potential transcription factors of miRNAs

Using the Funrich program 3.1.3, which is generally used for the investigation of functional enrichment and interaction networks of genes and proteins, the biological pathways, molecular functions, and proximal transcription factors of the miRNAs were predicted.^[26]^

### 2.9. Statistical analysis

Statistical analyses of the included population’s characteristics are shown as the means ± SD. The significance between the means was examined using the Student’s *t* test with *P* value < .05 being regarded as statistically significant. The normally distributed continuous variables are expressed as means standard deviations. The data were examined using R software (version 4.1.2). The Spearman’s method was used to analyze the correlation between genes.

## 3. Result

### 3.1. Identification of DE-mRNAs

**Figure 2.**
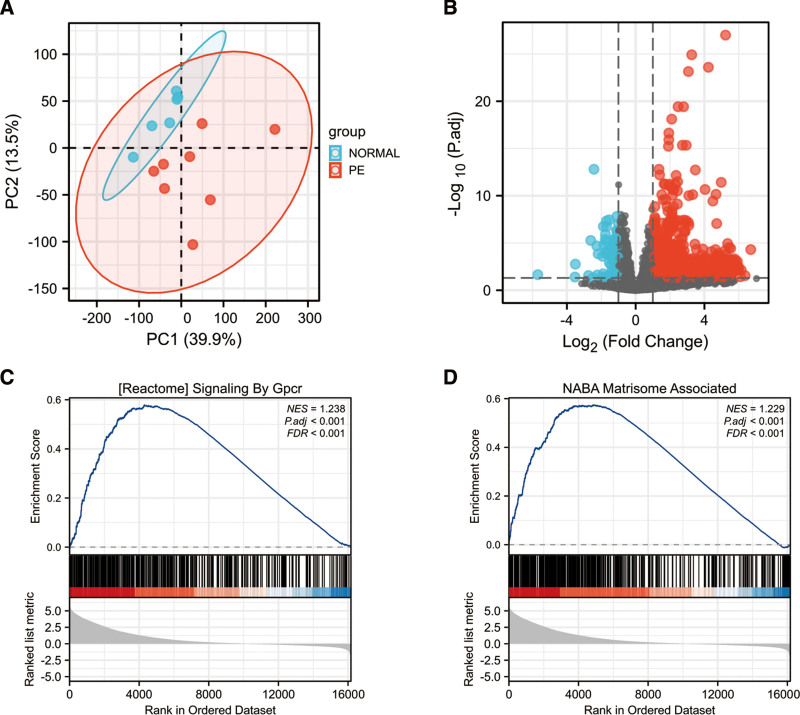
The PCA analysis, the volcano plot of the identified DE-mRNAs and representative results of GSEA analysis in the gene expression data in GSE190971. (A) The visualization of principal component analysis for GSE190971. (B) Volcano plot of the identification of DE-mRNAs, including 1880 upregulated and 96 downregulated genes, Red, upregulation; Blue, downregulation. (C) The most significant enriched gene set correlated with the PE group was signaling by Gpcr (NES = 1.238, *P*.adj < .001, FDR < 0.001). (D) The second significant enriched gene set correlated with the PE group was matrisome associate (NES = 1.229, *P*.adj < .001, FDR < 0.001). DE-mRNAs = differentially expressed mRNAs, FDR = false discovery rate, Gpcr = G-protein coupled receptor, GSEA = gene set enrichment analysis, NES = normalized enrichment score, PCA = principal component analysis.

Firstly, a principal component analysis of gene expression in all samples demonstrated that the repeatability of the data in GSE190971 was adequate (Fig. 2A).The differential expression analysis of the GSE190971 dataset demonstrated a total of 1976 DE-mRNAs were identified, including 1880 upregulated and 96 downregulated genes (Table S1, Supplemental Digital Content, http://links.lww.com/MD/H844). The resulting volcano plot shows upregulated and downregulated genes, which were marked in the red and blue (Fig. 2B). Next, GSEA on the gene expression data in GSE190971 was performed. The most enriched item identified by GSEA was signaling by Gpcr (G Protein-Coupled Receptors) (normalized enrichment score = 1.238, FDR < 0.001, *P*.adj < .001) (Fig. 2C). The second significant enriched gene set correlated with the PE group was matrisome associate (normalized enrichment score = 1.229, FDR < 0.001, *P*.adj < 0.001) (Fig. 2D).

### 3.2. Ferroptosis-related DE-mRNAs

**Figure 3.**
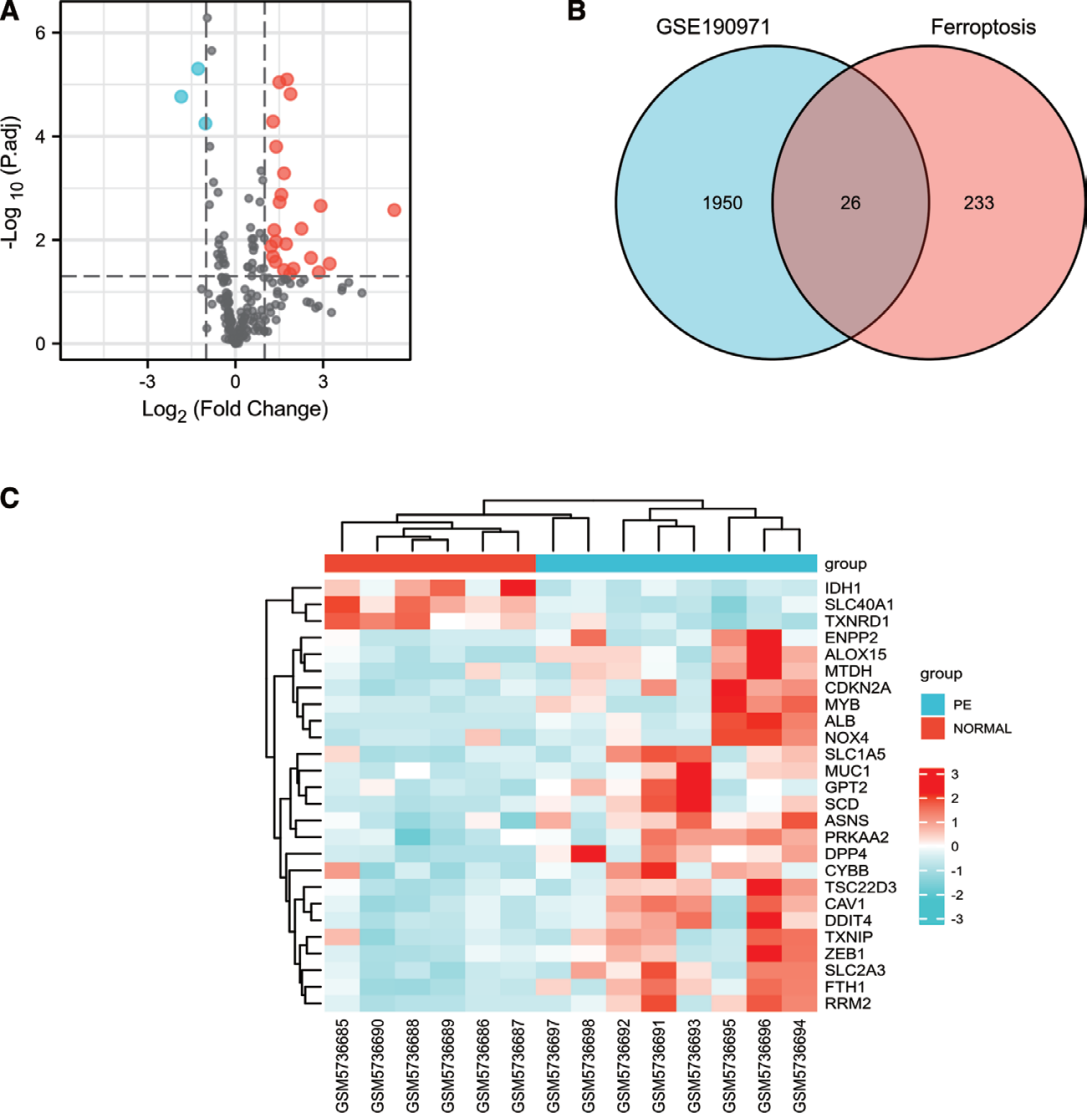
Ferroptosis-related DE-mRNAs between PE STB-EVs and normal STB-EVs samples. (A) Volcano plot of 216 ferroptosis-related genes including 23 upregulated and 3 downregulated genes, Red, upregulation; blue, downregulation. (B) Venn diagram to screen ferroptosis-related DE-mRNAs. (C) The heat map of 26 ferroptosis-related DE-mRNAs between PE STB-EVs and Normal STB-EVs samples. DE-mRNAs = differentially expressed mRNAs, PE = preeclampsia, STB-EVs = syncytiotrophoblast-derived extracellular vesicles.

After intersecting 259 ferroptosis-related genes with GSE190971 dataset, 216 ferroptosis-related genes were identified and volcano plots showed a global overview of 216 ferroptosis-related genes expression changes (Fig. 3A). Next, we combined the analysis of the DE-mRNAs in STB-EVs and 216 ferroptosis-related genes and the Venn diagram in Figure 3B showed 26 ferroptosis-related DE-mRNAs, including 23 upregulated and 3 downregulated genes (Fig. 3B). The heat map of the 26 ferroptosis-related DE-mRNAs were shown in Figure 3C. The 26 ferroptosis-related DE-mRNAs were classified further by the ferrdb online tool as either a ferroptosis driver, ferroptosis suppressor, or ferroptosis marker (Table 2).

**Table 2:** The ferroptosis-related DE-mRNAs were divided into ferroptosis driver, suppressor, and marker.

Marker	Driver	Suppressor
ALB, ALOX15, ASNS, DDIT4	CDKN2A, CYBB, DPP4, IDH1	
FTH1, GPT2, RRM2, SLC2A3	MTDH, MYB, NOX4, PRKAA2	CAV1, ENPP2, MUC1, SCD
SLC40A1, TSC22D3, TXNIP	SLC1A5, ZEB1, ALOX15	FTH1, SLC40A1
TXNRD1		

DE-mRNAs = differentially expressed mRNAs.

### 3.3. GO terms and KEGG pathway enrichment analysis of ferroptosis-related DE-mRNAs

**Figure 4.**
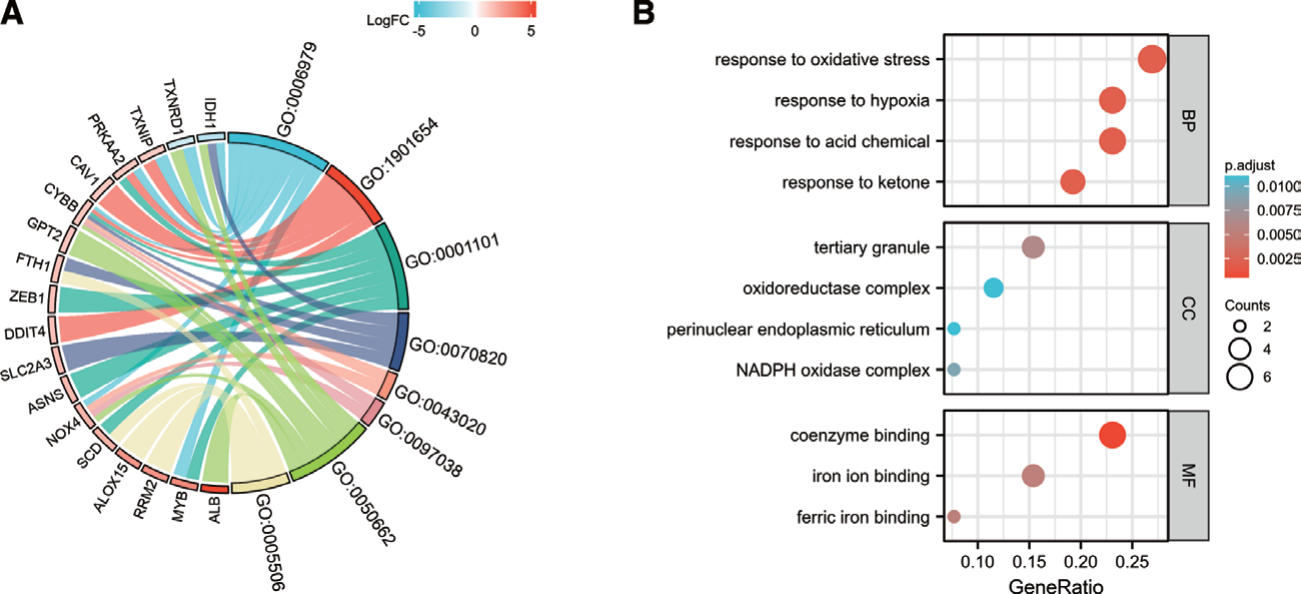
GO functional analysis of 26 ferroptosis-related DE-mRNAs, Chord (A) and Bubble plot (B) of enriched GO terms of 26 ferroptosis-related DE-mRNAs, Y-axis: name of GO items; X-axis: percentage of the number of genes assigned to a term mong the total number of genes annotated in the network; Bubble size, number of genes assigned to a pathway; Color: enriched −log10(*P*-value). DE-mRNAs = differentially expressed mRNAs, GO = gene ontology.

**Figure 5.**
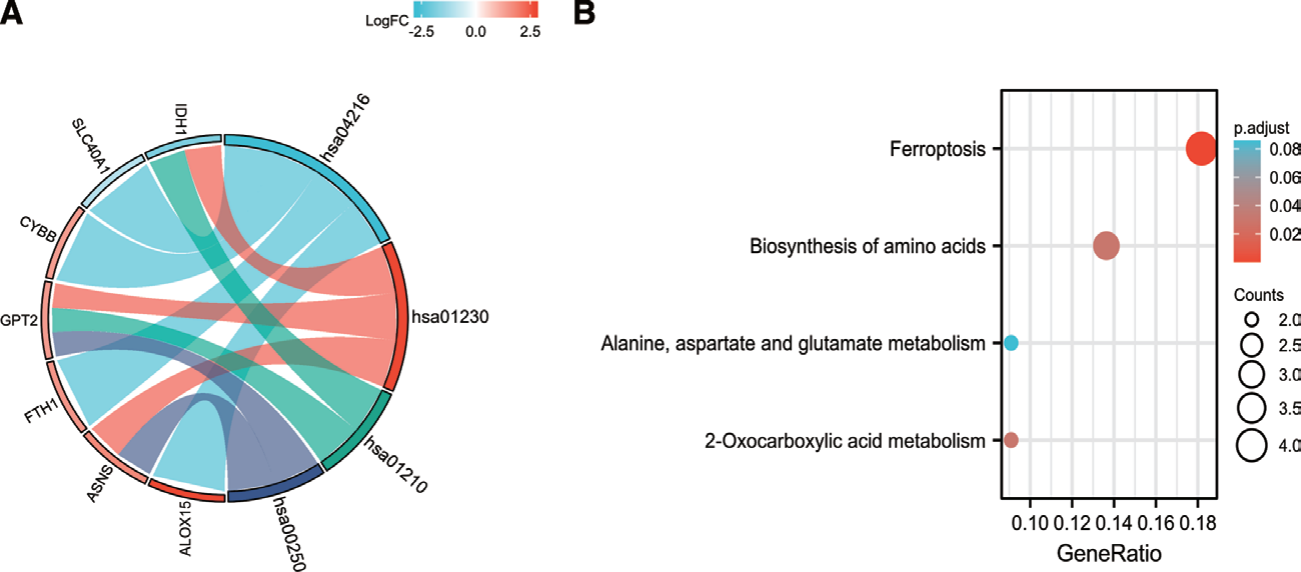
KEGG of 26 ferroptosis-related DE-mRNAs, Chord (A) and Bubble plot (B) of enriched KEGG terms of 26 ferroptosis-related DE-mRNAs, Y-axis: name of the KEGG signaling pathway; X-axis: percentage of the number of genes assigned to a term among the total number of genes annotated in the network; Bubble size, number of genes assigned to a pathway; Color: enriched −log10(*P*-value). DE-mRNAs = differentially expressed mRNAs, KEGG = Kyoto Encyclopedia of Genes and Genomes analysis.

Analysis for GO-biological process revealed that these 26 DE-mRNAs related to ferroptosis were substantially enriched in a variety of biological processes, including such response to oxidative stress, response to ketone, response to acid chemical, and response to hypoxia (Fig. 4). The top three significantly enriched terms in the GO cellular component analysis were tertiary granule, NADPH oxidase complex, perinuclear endoplasmic reticulum and oxidoreductase complex (Fig. 4). The top three significantly enriched terms in the GO molecular function analysis were coenzyme binding, iron ion binding, and ferric iron binding. Figure 5 displays the enriched KEGG items, which include ferroptosis, amino acid biosynthesis, alanine, aspartate, and glutamate metabolism, and 2-oxocarboxylic acid metabolism.

### 3.4. Correlation analysis, PPI network construction, and hub genes identification

**Figure 6.**
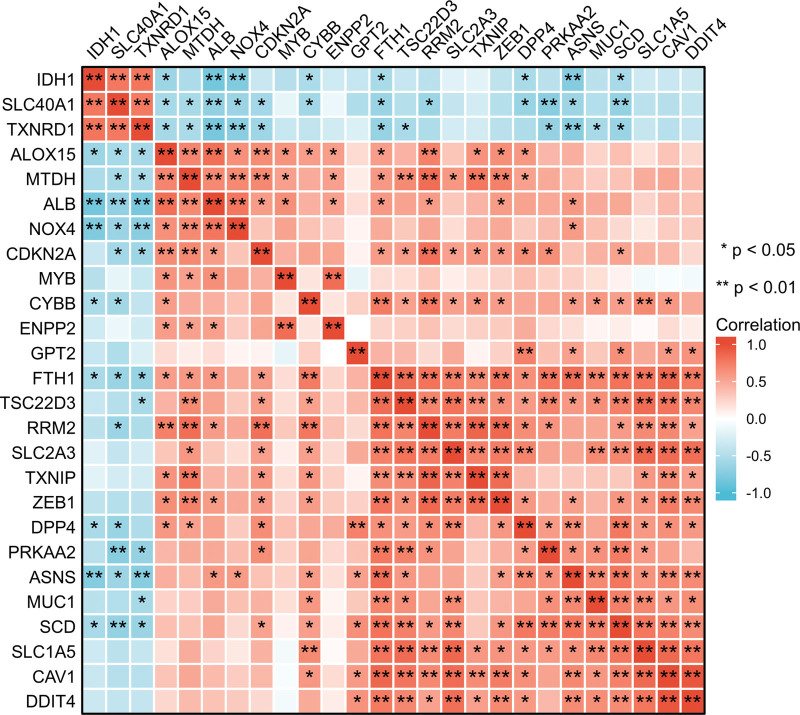
Spearman correlation analysis of the 26 ferroptosis-related DE-mRNAs. DE-mRNAs = differentially expressed mRNAs.

**Figure 7.**
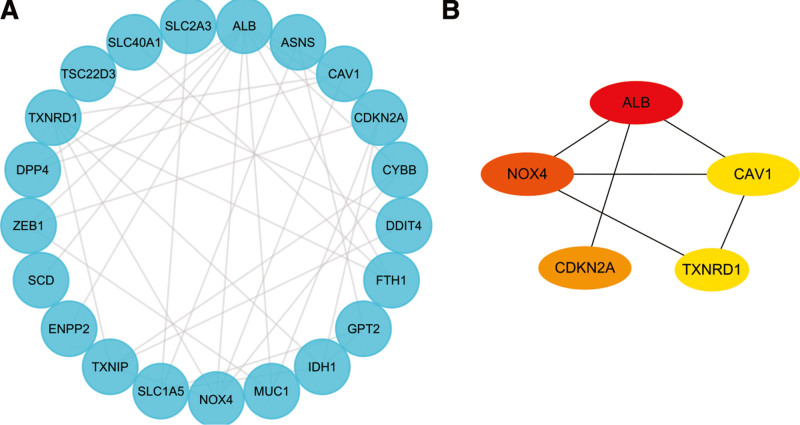
Identification of ferroptosis-related hub genes in PE STB-EVs. (A) PPI network constructed with ferroptosis-related DE-mRNAs were performed using the STRING, the nodes represent proteins, and the edges represent the interaction of the proteins. (B) Cytohubba in Cytoscape was used to find the top five hub genes in the PPI network by Degree, PPI network of the top 5 hub genes was visualized by Cytoscape, and the top 5 hub genes are displayed from red (high Degree value) to yellow (low Degree value). DE-mRNAs = differentially expressed mRNAs, PE = preeclampsia, PPI = protein-protein interaction, STB-EVs = syncytiotrophoblast-derived extracellular vesicles.

A correlation analysis was performed to investigate the expression correlation of these 26 ferroptosis-related DE-mRNAs. The correlation heatmap showed these are synergistic interactions on expression the relationship between the 26 ferroptosis-related DE-mRNAs (Fig. 6). We utilized the Cytoscape software to display the PPI network of DE-mRNAs acquired from the STRING database (Fig. 7A). Twenty-six nodes (genes) and 33 edges (interactions) composed the PPI network, with an enrichment *P* value of less than 1.11E-08. The top five nodes in the PPI network were constructed using Cytoscape’s Cytohubba plugin by the degree method. Albumin (ALB) had the highest connectivity degree, followed by Nicotinamide adenine dinucleotide phosphate (NADPH) oxidase 4 (NOX4), cyclin dependent kinase inhibitor 2A (CDKN2A), thioredoxin reductase 1 (TXNRD1) and caveolin 1 (CAV1) (Fig. 7B).

### 3.5. Construction of the logistic regression for hub genes

**Figure 8.**
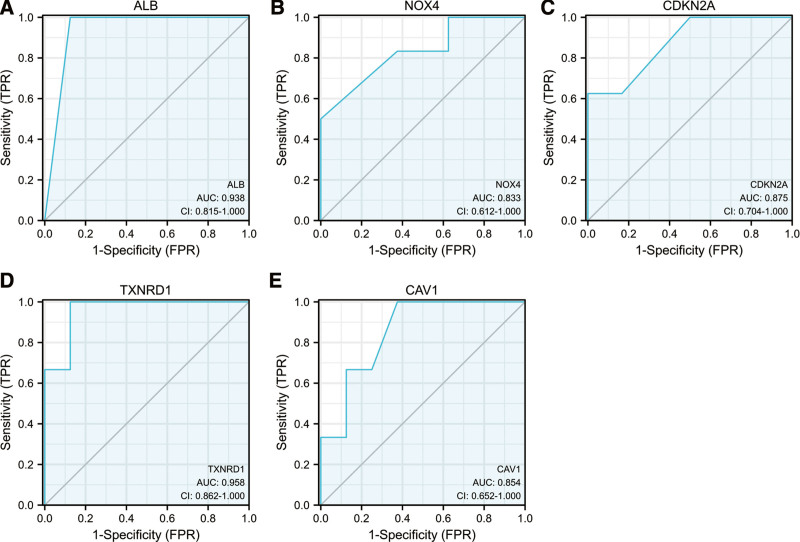
Receiver operating characteristic analysis revealed the predictive performance of ferroptosis-related hub genes for PE. (A) The AUC of ALB (A), NOX4 (B), CDKN2A (C), TXNRD1 (D), CAV1 (E) in ROC monofactor analysis. ALB = albumin, AUC = area under the curve, CAV1 = caveolin 1, CDKN2A = cyclin dependent kinase inhibitor 2A, NOX4 = nicotinamide adenine dinucleotide phosphate (NADPH) oxidase 4, ROC = receiver operating characteristic, TXNRD1 = thioredoxin reductase.

The areas under the ROC curves by univariate ROC analysis for ALB, NOX4, CDKN2A, TXNRD1 and CAV1 were 0.938 (CI: 0.815−1.000), 0.833 (CI: 0.612−1.000), 0.875 (CI: 0.704−1.000), 0.958 (CI: 0.862−1.000) and 0.854 (CI: 0.652−1.000), respectively (Fig. 8). The predictive performance of five ferroptosis-related hub genes in preeclampsia was observed.

### 3.6. Further miRNAs prediction of hub genes

**Figure 9.**
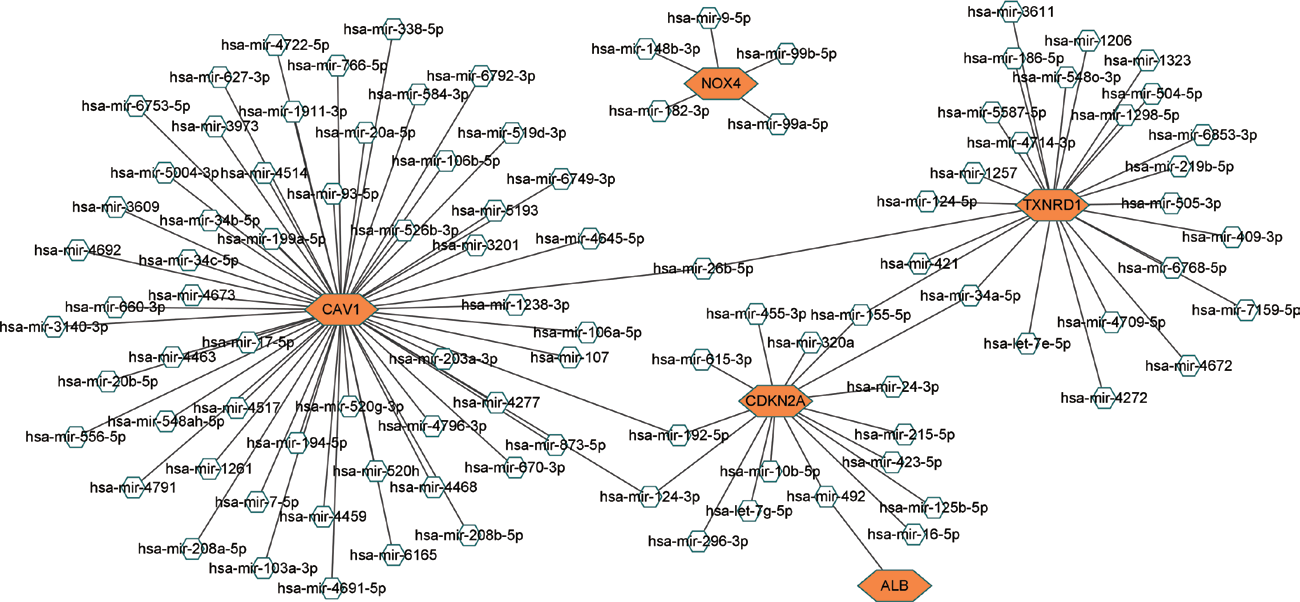
A miRNA–hub gene regulatory network construction in PE STB-EVs, Interaction network between hub genes and its targeted miRNAs, Genes were colored in red, miRNAs were colored in blue, the higher amounts of cross-linked genes including hsa-miR-26b-5p, hsa-miR-192-5p, hsa-miR-124-3p, hsa-miR-492, hsa-miR-34a-5p and hsa-miR-155-5p. PE = preeclampsia, STB-EVs = syncytiotrophoblast-derived extracellular vesicles.

**Figure 10.**
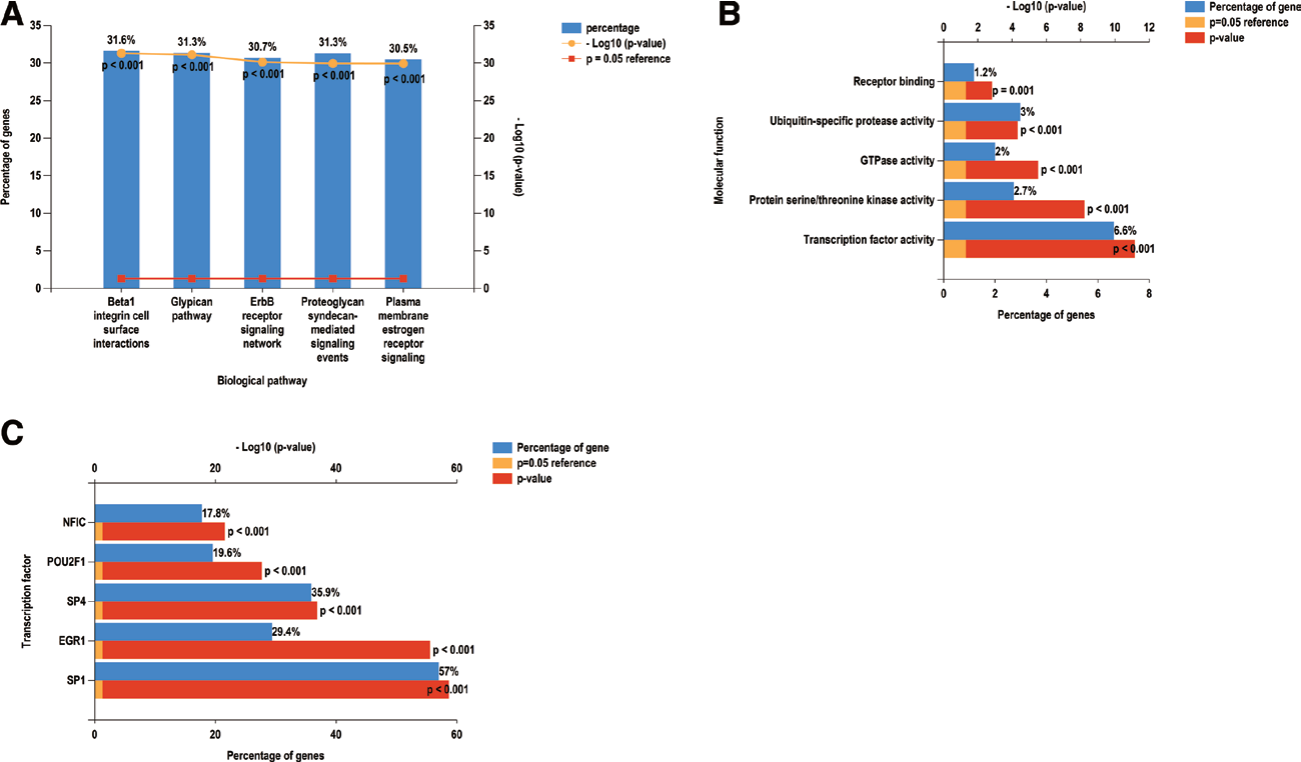
Biological pathways, (A) the molecular function, (B) and prediction of potential transcription factors (C) of miRNAs.

We constructed a miRNA–hub gene regulatory network in PE STB-EVs and hsa-miR-26b-5p, hsa-miR-192-5p, hsa-miR-124-3p, hsa-miR-492, hsa-miR-34a-5p and hsa-miR-155-5p had higher amounts of cross-linked genes (≥2) (Fig. 9). Subsequently, we uploaded 99 miRNAs to Funrich and the results of the enrichment analysis showed that the biological pathways enriched included Beta 1 integrin cell surface interactions, Glypican pathway, ErbB receptor signaling network, Plasma membrane estrogen receptor signaling and Proteglycan syndecan-mediated signaling events (Fig. 10A). The molecular functions were significantly enriched in Transcription factor activity, Protein serine/threonine kinase activity, gtpase activity, Ubiquitin-specific protease activity and Receptor binding (Fig. 10B). The SP1, EGR1, SP4, POU2F1, and NFIC were the top five transcription factors for these miRNAs, as depicted in Figure 10C.

## 4. Discussion

Ferroptosis, a particular type of programmed cell death spurred on by an iron-dependent accumulation of phospholipid peroxides,^[27]^ was first identified in the Stockwell Lab in 2012. Previous studies have shown that preeclampsia was considered to be associated with high iron status and ferroptosis. Placenta^[28,29]^ and different cell lineages at the maternal-fetal interface,^[30]^ including trophoblasts, dendritic cells, stromal fibroblasts, and decidual cells are proved to have different modes of intracellular iron regulation and distinctive sensitivities to ferroptosis. However, many ferroptosis genes have yet to be characterized, and ferroptosis-related genes in STB-EVs are not explored. Ferroptosis in STB-EVs of preeclampsia needs to be further comprehensively investigated. Our present study for the first time revealed ferroptosis-related DE-mRNAs in STB-EVs of PE using mRNA expression profile data from NCBI’s GEO public database. In this study, we identified 26 ferroptosis-related DE-mRNAs, including 23 upregulated and 3 downregulated genes from the intersection of DE-mRNAs and Ferroptosis Database. The differentially expressed ferroptosis-related genes are mainly enriched in response to oxidative stress, amino acid metabolism and iron related pathways. Next, we used these ferroptosis-related DEGs to identify six hub genes from the constructed PPI network (ALB, NOX4, CDKN2A, TXNRD1, and CAV1). We constructed a miRNA–hub gene regulatory network and the results showed that hsa-miR-26b-5p, hsa-miR-192-5p, hsa-miR-124-3p, hsa-miR-492, hsa-miR-34a-5p and hsa-miR-155-5p had higher amounts of cross-linked genes (≥2). In summary, this bioinformatics identification may indicate the treatment and early identification of preeclampsia and provide clues for STB-EVs dysfunction at the molecular level.

STB-EVs enters the maternal circulation from the first month of pregnancy to delivery and mediate the communications between the placenta and peripheral vascular endothelial cells.^[31,32]^ Under pathological conditions, STB-EVs affect the anti-angiogenesis and oxidative stress of maternal endothelial cells, which is considered as a stimulus for systemic inflammatory reaction and endothelial cell damage in preeclampsia.^[33]^ Recently, Gpcrs have been considered as an important participant in the pathogenesis of preeclampsia, rising during normal pregnancy and decreasing in patients with preeclampsia.^[34]^ The results of GSEA showed significantly differentially expressed genes related to signaling by Gpcr in STB-EVs between preeclampsia and normal pregnancy. Here, we provide additional evidence that STB-EVs in patients with preeclampsia may lead to disease progression by Gpcr pathway. Some studies have confirmed the close relationship between endothelial cell damage and ferroptosis.^[35,36]^ Ferroptosis increases cellular reactive oxygen species (ROS), monocyte adhesion and damage to endothelial cell derived nitric oxide, eventually leading to endothelial damage/death and increased vascular permeability.^[37]^ In contrast, the ferroptosis inhibitor ferrostatin-1(Fer-1) increases endothelial cell activity, reduces cell death, downregulates the expression of adhesion molecules (intercellular adhesion molecule-1 and vascular cell adhesion molecule-1), weakens the expression of VEGFA (vascular endothelial growth factor A), and upregulates endothelial nitric oxide synthase by inhibiting ferroptosis in mouse aortic endothelial cells exposed to oxidized low density lipoprotein.^[38]^ In our study, we identified 26 ferroptosis-related DEGs in STB-EVs which are mainly enriched in response to oxidative stress, amino acid metabolism and iron related pathways and these pathways are closely related to systemic inflammatory reaction and endothelial cell damage.^[38–40]^ Thus, we suggested that ferroptosis-related genes in STB-EVs can vary under physiological or pathological environments and are capable of affecting endothelial cell.

Next, we reviewed the relationship between six hub-gene of 26 ferroptosis-related DE-mRNAs and preeclampsia. ALB, NOX4 and CAV1 are associate with the prosses of preeclampsia, and the other two genes (CDKN2A, TXNRD1) have not been linked directly to the occurrence of preeclampsia. ALB, which encodes the most abundant protein in human blood, has been proved to be a strong predictor of preeclampsia,^[41]^ especially can assess blood-brain barrier integrity and neuroinflammation in preeclampsia.^[42]^ ALB is highly expressed in the serum of preeclampsia, and our study also shows that ALB expression is up-regulated in STB-EVs derived from preeclampsia. ALB, the ALB encodes protein, is a nonspecific transport protein that can bind many inorganic ions and insoluble small molecules and convert them into soluble substances, which play a physiological function in the body. ALB may cooperate with transferrin to limit the supply of iron, which may lead to preeclampsia.^[43]^ Ferroptosis associated with ALB may act in hypertension through pathways such as retinol metabolism, branched-chain amino acid metabolism, drug metabolism-cytochrome P450 and biological processes.^[44]^ PE showed elevated gene and protein expression of NOX4, one of the causes of cellular ROS. Vascular pathogenic diseases have been conclusively associated with excessive ROS production.^[45]^ NOX4 in a sflt1-based model of early-onset preeclampsia may mediate oxidative stress and possibly inflammatory processes.^[46]^ Ferroptosis is characterized by the toxic lipid ROS accumulation. Some studies have demonstrated that NOX4 contributes to ferroptosis of astrocytes through oxidative stress-induced lipid peroxidation in Alzheimer’s disease.^[47]^ NOX4 was upregulating through the activation of endoplasmic reticulum stress-induced activation of ATF3 thereby which led to produce hydrogen peroxide to promote ferroptosis in glioma cells.^[48]^ Similarly, our study shows that NOX4 mRNA expression is identified as down regulated in STB-EVs derived from preeclampsia, which indicates the potential role of NOX4 as an Ferroptosis Driver in STB-EVs derived from preeclampsia. CAV1 is a scaffold protein of caveolae and plays a crucial role in maintaining the curved structure as well as functions of caveolae. CAV-1 is an important mechanism mediating oxidant induced hyperpermeability of vascular.^[49]^ CAV1 is abnormally up-regulated in the placenta of preeclampsia, which affects the invasion of trophoblast cells through CAV-1/Met/HGF signal pathway and promotes the development of preeclampsia.^[50]^ CAV-1 can scavenge the intracellular reactive species and is involved in antioxidant prevention after ROS-induced oxidative stress in astrocytes.^[51]^ In the type 2 diabetes mellitus population, neuron-targeted CAV-1 overexpression activated AMPK and subsequently increased NRF2 and FPN expressions, and ultimately suppressed ferroptosis.^[52]^ Interestingly, although CAV-1 has been proved to be an Ferroptosis inhibitor in the Ferroptosis database, CAV-1 has been identified as up-regulated in STB-EVs derived from preeclampsia in our study. This comparison shows that CAV-1 may have different functions in different disease environments. CDKN2A is ferroptosis driver and TXNRD1 is ferroptosis marker in the Ferroptosis Database. These two gene have been shown to affect ferroptosis pathway from studies performed on various diseases, but no previous study has identified the association with preeclampsia. Our results indicate that CDKN2A and TXNRD1 are highly expressed in STB-EVs derived from preeclampsia, further studies are needed to identify the impact of CDKN2A and TXNRD1 on ferroptosis pathway.

## 5. Conclusion

In this investigation, we demonstrated numerous key genes (ALB, NOX4, CDKN2A, TXNRD1, and CAV1) that are expected to act as biomarkers for ferroptosis in STB-EVs of preeclampsia. Some of them were only recently mentioned in the literature in relation to preeclampsia. These results might uncover new knowledge on how ferroptosis-related genes are regulated as well as identifying new candidate genes. Overall, our findings will ultimately present pertinent evidence for the role of ferroptosis in preeclampsia. However, the deeper mechanism of ferroptosis in STB-EVs and its function in the pathogenesis of preeclampsia require more study.

## Author contributions

**Conceptualization:** Xueqin Zhang.

**Data curation:** Wei Wei.

**Formal analysis:** Wei Wei.

**Funding acquisition:** Xueqin Zhang.

**Investigation:** Huanxi Li.

**Methodology:** Huanxi Li, Wei Wei, Xueyan Lin.

**Project administration:** Huanxi Li, Xueyan Lin.

**Resources:** Weiwei Yu.

**Software:** Weiwei Yu, Xueyan Lin.

**Supervision:** Weiwei Yu.

**Validation:** Xiang Ying.

**Visualization:** Quanfeng Wu.

**Writing – original draft:** Quanfeng Wu, Xiang Ying.

**Writing – review & editing:** Quanfeng Wu, Xiang Ying.

## Supplementary Material


