# Supplementary material for: Identification of ferroptosis-related genes in syncytiotrophoblast-derived extracellular vesicles of preeclampsia
Source: Medicine (Baltimore). 2022 Nov 4;101(44):e31583. doi: 10.1097/MD.0000000000031583 (PMC9646584; doi:10.1097/MD.0000000000031583)
Supplement: Supplementary file 1 [file medi-101-e31583-s001.pdf]

id	baseMean	log2FoldChange	lfcSE	stat	pvalue	padj
A1CF	8.295643	5.959823	1.56577	3.80631	0.000141	0.00283
AADAT	7.147836	3.284278	0.79777	4.11682	3.84E-05	0.00106
AASS	20.66291	1.946225	0.6238	3.11995	0.001809	0.015833
ABCA1	196.9982	1.075252	0.32801	3.27812	0.001045	0.010968
ABCA12	25.38591	2.034334	0.71972	2.82656	0.004705	0.029562
ABCA13	20.80088	2.295262	0.83321	2.75472	0.005874	0.034026
ABCA17P	3.718732	5.211693	1.43345	3.63577	0.000277	0.004526
ABCA3	2.803627	3.846925	1.05055	3.66183	0.00025	0.004218
ABCA7	11.9747	1.536257	0.51482	2.98405	0.002845	0.02136
ABCA8	9.737935	3.214558	1.01753	3.15916	0.001582	0.014584
ABCB11	2.925585	3.843166	1.46357	2.62589	0.008642	0.043324
ABCB5	11.38569	5.423432	1.43476	3.78004	0.000157	0.003065
ABCC9	12.34539	3.953262	0.98676	4.00629	6.17E-05	0.001548
ABCD2	7.48242	2.792828	1.0551	2.64698	0.008121	0.041551
ABHD2	16.89635	1.114353	0.38544	2.8911	0.003839	0.026147
ABI3BP	12.79183	2.796295	0.77801	3.59418	0.000325	0.005064
ABLIM3	28.67073	1.86818	0.43661	4.27884	1.88E-05	0.000614
ACAP1	7.524122	1.621669	0.61345	2.64351	0.008205	0.041741
ACKR3	13.96508	2.047564	0.61496	3.32961	0.00087	0.009689
ACKR4	1.690322	4.026024	1.57426	2.55741	0.010545	0.049417
ACOT4	31.94865	-1.08207	0.36023	-3.0039	0.002666	0.020434
ACOXL	195.9034	-1.01143	0.2264	-4.4675	7.92E-06	0.000324
ACP6	19.44818	1.044759	0.31618	3.30431	0.000952	0.010278
ACSM1	6.032311	4.450732	1.41892	3.13671	0.001709	0.015286
ACSS3	10.16864	2.652344	1.00129	2.64894	0.008074	0.041429
ACTN1	68.60407	1.352274	0.30088	4.49441	6.98E-06	0.000292
ADA	2.780251	3.353949	1.31322	2.55399	0.01065	0.049588
ADAD1	4.172711	4.946844	1.60367	3.0847	0.002038	0.017059
ADAM20	9.84865	2.419209	0.90589	2.67054	0.007573	0.039805
ADAM22	15.28208	3.721227	1.15586	3.21946	0.001284	0.012703
ADAM28	12.17895	3.642067	1.08129	3.36826	0.000756	0.008888
ADAM32	1.420913	3.921516	1.50645	2.60315	0.009237	0.045407
ADAM9	130.5419	1.09599	0.22743	4.81903	1.44E-06	8.21E-05
ADAMTS1	22.07584	1.938805	0.40959	4.73356	2.21E-06	0.000115
ADAMTS20	15.7618	4.334039	1.07633	4.0267	5.66E-05	0.001438
ADAMTS5	13.09089	1.492149	0.50463	2.95689	0.003108	0.022703
ADAMTS9	11.01317	2.81549	0.91361	3.08171	0.002058	0.017194
ADAMTSL3	9.476522	3.905851	1.09113	3.57964	0.000344	0.005268
ADAT2	7.757415	3.715517	1.10683	3.35689	0.000788	0.009168
ADCY1	9.66093	4.703695	1.24493	3.77828	0.000158	0.003077
ADCY10P1	6.619845	4.557223	1.26941	3.59003	0.000331	0.00512
ADD2	4.782876	3.310702	1.1924	2.7765	0.005495	0.032731
ADGB	12.66366	3.939739	1.29193	3.0495	0.002292	0.018585

ADGRE4P	4.87618	5.167061	1.85665	2.783	0.005386	0.032321
ADGRF1	6.25016	3.710013	1.21692	3.04868	0.002298	0.018617
ADGRF5	9.25461	1.6943	0.6072	2.79035	0.005265	0.031797
ADGRG1	97.43557	1.078316	0.29503	3.65497	0.000257	0.004302
ADGRG4	4.552291	4.028419	1.42952	2.81803	0.004832	0.030045
ADGRG7	3.252252	4.495644	1.56575	2.87124	0.004089	0.027179
ADH1B	6.591873	3.343797	1.21083	2.76156	0.005753	0.033574
ADIRF	248.7479	-1.08762	0.21866	-4.9741	6.56E-07	4.29E-05
ADRA1A	17.43804	4.082216	1.17426	3.47643	0.000508	0.006909
ADRA2C	6.071885	2.134454	0.80653	2.64646	0.008134	0.041565
ADSS1	20.39641	-1.18518	0.43916	-2.6988	0.00696	0.037645
AFAP1	117.4915	2.305902	0.29677	7.76989	7.86E-15	5.94E-12
AFAP1L2	7.206636	2.220933	0.56597	3.92409	8.71E-05	0.002002
AFF3	111.9992	-1.74441	0.37115	-4.7	2.60E-06	0.000129
AFM	4.26953	4.427109	1.65787	2.67035	0.007577	0.03981
AGAP11	11.68887	-1.05889	0.38107	-2.7787	0.005457	0.032572
AGBL3	7.279536	2.566402	0.88601	2.89659	0.003772	0.025777
AGMO	4.300644	4.976231	1.57752	3.15446	0.001608	0.014704
AGPAT1	4.487933	-2.74396	0.95981	-2.8589	0.004252	0.027841
AGPAT5	413.1159	-1.23217	0.47265	-2.6069	0.009136	0.045142
AGXT2	5.345279	3.787607	1.12965	3.35289	0.0008	0.009229
AHNAK2	9.957996	1.924572	0.67012	2.872	0.004079	0.027159
AHRR	8.588824	1.50205	0.4971	3.02163	0.002514	0.019773
AHSP	13.41883	2.116514	0.81917	2.58373	0.009774	0.047028
AICDA	22.87105	-1.25813	0.37704	-3.3369	0.000847	0.009519
AIF1L	218.8066	1.335565	0.41001	3.25739	0.001124	0.011564
AK4	30.04944	2.686298	0.57313	4.68708	2.77E-06	0.000136
AKAP1	9.941107	1.618211	0.55657	2.90745	0.003644	0.025218
AKAP6	14.85976	2.366883	0.83501	2.83457	0.004589	0.02909
AKNA	28.40596	1.368178	0.51868	2.6378	0.008345	0.042211
AKR1C6P	3.097389	4.973931	1.73764	2.86247	0.004204	0.02767
ALB	4.252465	5.435157	1.41947	3.829	0.000129	0.002644
ALCAM	10.66593	2.588333	0.79977	3.23636	0.001211	0.012119
ALDH1A1	14.28302	1.875985	0.62833	2.98565	0.00283	0.021288
ALDH1B1	5.007013	3.091356	0.80528	3.83884	0.000124	0.002574
ALDH1L1	2.422918	3.540818	1.29937	2.72503	0.00643	0.035906
ALDH1L2	6.104613	3.300515	1.24177	2.65791	0.007863	0.040806
ALDH7A1	22.40005	1.044436	0.31823	3.282	0.001031	0.010863
ALDOB	2.239006	4.5116	1.74351	2.58766	0.009663	0.046642
ALOX15	2.755411	2.850541	1.08007	2.63921	0.00831	0.042098
ALX1	3.611005	5.208144	1.77228	2.93867	0.003296	0.023569
AMIGO2	6.076522	2.209212	0.74047	2.98352	0.00285	0.021373
AMOTL2	239.4417	1.141197	0.20783	5.49107	3.99E-08	4.39E-06
AMPH	6.038543	3.658581	1.37149	2.6676	0.007639	0.039996

ANGPTL1	4.02589	3.780421	1.14204	3.31024	0.000932	0.010155
ANGPTL5	4.612715	5.089361	1.79766	2.8311	0.004639	0.029364
ANK2	19.02952	3.262561	0.92897	3.51204	0.000445	0.006273
ANKRD18A	7.291941	2.27878	0.81704	2.78906	0.005286	0.03189
ANKRD20A5P	26.48322	-1.0932	0.38672	-2.8269	0.0047	0.029558
ANKRD20A8P	6.836237	3.969343	1.395	2.8454	0.004436	0.028569
ANKRD26P1	9.520297	2.516742	0.96431	2.60988	0.009057	0.044876
ANKRD26P3	5.203386	3.749	1.38573	2.70544	0.006821	0.037257
ANKRD30A	8.880893	4.255658	1.24008	3.43176	0.0006	0.007682
ANKRD30B	12.23072	4.672374	1.29588	3.60556	0.000311	0.004908
ANKRD30BL	2.558715	4.679052	1.82583	2.5627	0.010386	0.048983
ANKRD30BP2	4.479079	5.012639	1.51613	3.30622	0.000946	0.01026
ANKRD31	11.41949	3.156879	0.93404	3.3798	0.000725	0.008617
ANKRD33B	10.80402	2.159386	0.60659	3.55989	0.000371	0.005575
ANKRD36	10.91766	3.63811	1.16584	3.12059	0.001805	0.015825
ANKRD36B	4.002793	4.340937	1.49322	2.9071	0.003648	0.025236
ANKRD36C	13.54102	2.726807	0.99341	2.74489	0.006053	0.034629
ANKRD37	7.035523	2.171871	0.58202	3.73159	0.00019	0.003496
ANKRD45	7.674339	3.345603	1.21372	2.75649	0.005842	0.033905
ANKRD62	8.681294	3.480774	1.06279	3.27513	0.001056	0.011051
ANKRD65	12.26129	1.671063	0.49979	3.34355	0.000827	0.009424
ANLN	15.11721	1.644013	0.60165	2.73253	0.006285	0.035516
ANO4	4.488811	3.460308	1.29948	2.66284	0.007748	0.040347
ANTXR1	22.42895	1.495736	0.40574	3.68643	0.000227	0.003953
ANXA10	2.867669	4.864014	1.76373	2.7578	0.005819	0.033866
AOAH	7.733157	3.042634	1.16836	2.60418	0.009209	0.045323
AP1S2	26.8756	1.006567	0.37423	2.68973	0.007151	0.038269
APAF1	32.8236	1.079781	0.32746	3.29741	0.000976	0.010461
APCDD1	7.818658	1.826802	0.61581	2.96649	0.003012	0.022267
APLN	27.42494	1.909229	0.3799	5.02567	5.02E-07	3.58E-05
APOBEC3F	3.971506	2.10689	0.8181	2.57535	0.010014	0.047799
APOBEC3G	6.006799	3.382658	1.10523	3.06059	0.002209	0.018119
APOL4	85.85865	1.680082	0.50509	3.32629	0.00088	0.009759
APOL6	45.1511	2.023621	0.45939	4.40498	1.06E-05	0.000403
ARFGEF3	33.30354	1.057375	0.37117	2.84877	0.004389	0.028324
ARGFX	3.327917	4.570571	1.60703	2.84412	0.004453	0.028651
ARHGAP15	9.466173	2.370514	0.86172	2.7509	0.005943	0.034255
ARHGAP20	6.578694	3.57822	1.23964	2.8865	0.003895	0.026301
ARHGAP30	30.29844	1.365994	0.39308	3.47509	0.000511	0.006926
ARHGAP45	160.6937	2.188551	0.3514	6.22808	4.72E-10	9.31E-08
ARHGAP6	5.916247	2.769356	0.94418	2.93308	0.003356	0.023862
ARHGAP9	5.426136	1.733423	0.64816	2.67437	0.007487	0.039492
ARHGEF28	27.60482	2.307504	0.64652	3.56913	0.000358	0.005413
ARHGEF4	15.1502	2.932771	0.45832	6.39891	1.56E-10	4.22E-08

ARL13B	9.3966	3.336193	0.83684	3.98667	6.70E-05	0.001659
ARLNC1	5.260477	1.617242	0.58747	2.75291	0.005907	0.034094
ARMC3	8.708931	4.330912	1.29978	3.33204	0.000862	0.009626
ARMS2	769.3735	2.714085	0.48907	5.54946	2.87E-08	3.38E-06
ARNT2	170.9496	2.914995	0.46348	6.28937	3.19E-10	7.06E-08
ARPP21	15.33489	3.943578	1.075	3.66845	0.000244	0.004153
ARRDC4	28.83811	1.200894	0.47076	2.55098	0.010742	0.049876
ART4	3.204856	2.714041	1.03414	2.62445	0.008679	0.043441
ASAH1	387.7555	-1.1876	0.23596	-5.033	4.83E-07	3.52E-05
ASAP2	47.05652	1.144196	0.32942	3.47338	0.000514	0.00695
ASB15	3.316661	4.546729	1.7509	2.5968	0.00941	0.04589
ASB5	3.720509	3.738075	1.15242	3.24368	0.00118	0.011938
ASCL2	3.56527	2.000092	0.72682	2.75184	0.005926	0.034192
ASIP	2.045472	3.818756	1.47641	2.58651	0.009695	0.046761
ASNS	10.59	1.731258	0.53379	3.2433	0.001182	0.011946
ASPHD2	3.019806	4.404614	1.48171	2.97265	0.002952	0.021935
ASPM	22.28085	2.164106	0.78323	2.76304	0.005727	0.03349
ASTE1	57.41964	1.05139	0.40552	2.59268	0.009523	0.046184
ATL1	7.835183	3.753606	1.0835	3.46435	0.000532	0.007084
ATP10B	8.678631	3.474272	1.11412	3.11839	0.001818	0.015908
ATP11A	111.8925	1.091067	0.17238	6.32938	2.46E-10	5.85E-08
ATP11B	31.54224	1.483668	0.41975	3.53461	0.000408	0.005959
ATP13A5	6.550142	4.622946	1.73469	2.665	0.007699	0.040216
ATP2A3	15.76609	1.895912	0.56387	3.36233	0.000773	0.009037
ATP8	748.8114	1.060109	0.28762	3.68574	0.000228	0.003957
ATP8A1	18.72682	2.820416	0.69254	4.07256	4.65E-05	0.001228
ATP8B1	23.29473	1.009264	0.28419	3.55133	0.000383	0.005722
ATP8B4	16.67479	2.49947	0.78363	3.18961	0.001425	0.01358
ATP9A	14.21012	2.522235	0.77699	3.24616	0.00117	0.011856
ATRNL1	13.951	3.133238	0.96155	3.25851	0.00112	0.011525
ATXN8OS	3.339363	5.086611	1.75218	2.90302	0.003696	0.025404
AVPR1A	7.899682	3.049116	1.07954	2.82446	0.004736	0.029677
AXDND1	7.448358	3.689972	1.00607	3.6677	0.000245	0.004161
AXIN2	23.90192	2.642613	0.47541	5.55862	2.72E-08	3.26E-06
AZIN2	2.886168	4.889324	1.30517	3.74612	0.00018	0.00338
B2M	156.5188	1.147452	0.39112	2.93373	0.003349	0.023842
B3GAT1	2.786081	4.243922	1.52629	2.78055	0.005427	0.032492
B3GNT2	54.73586	-1.01488	0.31353	-3.2369	0.001208	0.012119
B4GALT6	7.017168	3.205639	1.04357	3.07181	0.002128	0.017676
BACE1	6.227056	2.6673	0.83527	3.19333	0.001406	0.013486
BAMBI	1.500485	3.246522	1.27112	2.55407	0.010647	0.049588
BATF3	2.949099	2.732975	0.94595	2.88914	0.003863	0.026185
BBOX1	36.45644	-1.03114	0.33683	-3.0613	0.002204	0.018086
BCAT1	71.80673	2.065389	0.43698	4.7265	2.28E-06	0.000117

BCHE	3.706888	5.232988	1.49473	3.50097	0.000464	0.006473
BCL11B	10.90534	2.26178	0.72781	3.10765	0.001886	0.016286
BCL6	260.3427	2.354459	0.292	8.0632	7.43E-16	7.07E-13
BCOR	17.03048	1.909589	0.43509	4.38896	1.14E-05	0.000425
BDH2	4.825778	4.592884	1.48189	3.09935	0.001939	0.016577
BEND2	12.97103	1.529148	0.59587	2.56623	0.010281	0.048729
BEST1	2.779265	4.770489	1.55414	3.06954	0.002144	0.017729
BHLHE22	2.973786	4.421665	1.26623	3.49199	0.000479	0.006614
BHLHE40	268.9213	1.84019	0.32251	5.70577	1.16E-08	1.55E-06
BHLHE40-AS1	2.938976	3.845894	1.11187	3.45893	0.000542	0.007193
BHMT2	2.542532	3.58346	1.39816	2.56299	0.010378	0.048983
BICC1	6.989252	3.897721	1.1704	3.33025	0.000868	0.009674
BICD1	12.96561	4.63613	1.061	4.36958	1.24E-05	0.000454
BIN2	115.438	2.150016	0.2842	7.56507	3.88E-14	2.24E-11
BIRC3	23.45681	1.543172	0.55949	2.75816	0.005813	0.03384
BIRC5	6.846673	2.385788	0.78807	3.02737	0.002467	0.019522
BIRC7	3.822716	2.659436	0.87638	3.03456	0.002409	0.019205
BMPER	5.282046	2.94625	0.95945	3.07076	0.002135	0.017686
BMPR1B	10.89754	2.232596	0.81747	2.73111	0.006312	0.035579
BNC2	23.18562	1.421018	0.49536	2.86867	0.004122	0.02729
BNIPL	28.25487	1.12079	0.2824	3.96874	7.23E-05	0.001754
BOC	3.818178	2.591127	0.9832	2.6354	0.008404	0.042418
BOLL	2.771002	3.761286	1.35516	2.77552	0.005511	0.032749
BRDT	5.842654	3.7499	1.09333	3.42979	0.000604	0.007714
BRINP3	3.439976	3.232706	1.05634	3.06029	0.002211	0.018129
BRIP1	19.67209	1.793102	0.62782	2.85609	0.004289	0.027926
BTBD8	13.13333	1.049183	0.40122	2.615	0.008923	0.044389
BTG2	889.7184	-1.0944	0.18573	-5.8924	3.81E-09	5.67E-07
BTN2A2	4.3909	3.134217	0.99754	3.14195	0.001678	0.015124
BTN2A3P	2.423014	4.595618	1.75573	2.61749	0.008858	0.044172
BTN3A1	7.758331	2.206151	0.75014	2.94098	0.003272	0.023456
BTN3A2	9.616029	1.58877	0.59294	2.67949	0.007373	0.039046
BUB1	8.524445	2.941725	1.1018	2.66994	0.007587	0.039835
BUB1B	8.451458	2.571351	0.94849	2.71099	0.006708	0.036838
BVES	3.720925	4.154848	1.38836	2.99264	0.002766	0.020914
C10orf67	7.640118	4.069419	1.18283	3.44041	0.000581	0.007554
C11orf87	4.443233	5.504782	1.58896	3.46439	0.000531	0.007084
C12orf40	3.590967	4.660209	1.50574	3.09496	0.001968	0.016723
C12orf42	13.84115	6.696857	1.35568	4.93984	7.82E-07	4.96E-05
C12orf50	4.734156	5.101342	1.68526	3.02704	0.00247	0.019522
C12orf75	233.0596	1.62888	0.32132	5.06933	3.99E-07	2.96E-05
C14orf39	14.78966	3.627489	0.98191	3.69433	0.00022	0.00387
C15orf48	7.043288	1.947403	0.6586	2.95688	0.003108	0.022703

C19orf33	7.266467	1.446282	0.56486	2.56044	0.010454	0.049188
C1orf21	133.3294	1.001629	0.21723	4.61102	4.01E-06	0.000182
C1QA	10.54658	2.024953	0.69099	2.93052	0.003384	0.023953
C1QB	12.87921	2.455244	0.83757	2.93138	0.003375	0.02392
C1QTNF7	4.044645	3.779265	1.29202	2.92509	0.003444	0.024189
C2CD6	9.23841	3.000074	1.02205	2.93535	0.003332	0.02376
C4BPA	2.576863	4.053517	1.24518	3.25536	0.001132	0.011617
C4orf47	3.118683	3.058852	1.1349	2.69525	0.007034	0.037887
C4orf54	6.922164	5.683245	1.45873	3.89601	9.78E-05	0.002156
C5orf46	22.33383	1.388474	0.44815	3.09826	0.001947	0.016607
C6	5.308854	4.768238	1.3948	3.41858	0.000629	0.007956
C8A	2.331494	4.017114	1.54612	2.59819	0.009372	0.045826
C8orf34	7.14657	3.645065	1.14447	3.18493	0.001448	0.013713
C8orf58	114.6231	1.945048	0.25276	7.69536	1.41E-14	9.12E-12
CA10	96.01323	-1.27213	0.49707	-2.5592	0.01049	0.049272
CA12	5.33459	3.173343	1.01909	3.11389	0.001846	0.01604
CA8	9.368459	2.482927	0.79776	3.11237	0.001856	0.016105
CACNA1D	6.382752	3.469207	1.14962	3.0177	0.002547	0.019902
CACNA1E	9.187776	5.125439	1.46179	3.50627	0.000454	0.006377
CACNA1I	2.49203	3.567524	1.38131	2.58271	0.009803	0.047137
CACNA2D1	19.28462	3.985676	1.1387	3.50021	0.000465	0.006479
CACNA2D1-AS1	4.90851	5.162957	1.64001	3.14813	0.001643	0.014944
CACNB2	3.721928	5.274657	1.95361	2.69995	0.006935	0.037611
CACNG8	4.234142	5.466541	1.95613	2.79457	0.005197	0.031527
CADM2	13.85549	2.973078	1.04175	2.85392	0.004318	0.028035
CADPS	7.151498	4.670947	1.41334	3.3049	0.00095	0.010278
CADPS2	11.37384	1.731255	0.65273	2.65233	0.007994	0.041197
CALCRL	15.98037	1.482727	0.54505	2.72037	0.006521	0.036215
CAMK2N1	43.31346	2.389638	0.6052	3.94853	7.86E-05	0.001856
CAMK4	6.473582	3.491458	1.23804	2.82015	0.0048	0.029963
CAPN13	3.293527	4.579664	1.57714	2.90378	0.003687	0.025364
CAPN2	57.89145	1.347168	0.33018	4.08008	4.50E-05	0.001207
CARD10	3.439764	2.762785	1.04353	2.64754	0.008108	0.041517
CARD17	14.21611	1.929113	0.64263	3.00189	0.002683	0.020542
CASP5	5.883542	4.925781	1.40427	3.50771	0.000452	0.006354
CASR	3.519695	4.646862	1.64335	2.82768	0.004689	0.02953
CAV1	63.35934	1.289644	0.2616	4.92992	8.23E-07	5.15E-05
CAV2	21.98483	1.8555	0.32541	5.70209	1.18E-08	1.56E-06
CAVIN2	125.2418	1.330995	0.44091	3.01871	0.002539	0.019864
CBLN2	5.530989	5.854877	1.70555	3.43283	0.000597	0.007658
CBLN4	4.19207	4.384416	1.49893	2.92504	0.003444	0.024189
CBX6	51.46573	1.018664	0.30119	3.38211	0.000719	0.008591
CCBE1	3.542211	4.638319	1.4807	3.13251	0.001733	0.015413

CCDC102B	10.37795	4.892135	1.38014	3.54467	0.000393	0.005788
CCDC110	2.098438	4.475999	1.5506	2.88662	0.003894	0.026301
CCDC138	17.2137	1.171851	0.31916	3.67168	0.000241	0.004122
CCDC141	11.39303	3.516355	1.13946	3.08598	0.002029	0.017021
CCDC144NL	7.948319	5.368272	1.36845	3.92287	8.75E-05	0.002007
CCDC146	10.46113	1.310883	0.46798	2.80115	0.005092	0.031111
CCDC158	8.948886	3.627555	1.03449	3.50661	0.000454	0.006375
CCDC168	10.99839	2.964301	0.9385	3.15854	0.001586	0.014607
CCDC172	4.908947	5.655341	1.71081	3.30566	0.000948	0.01026
CCDC179	2.661676	4.746603	1.81862	2.61	0.009054	0.044874
CCDC183	12.33361	1.191212	0.40609	2.93334	0.003353	0.023862
CCDC191	11.70525	2.715358	0.86867	3.12588	0.001773	0.015687
CCDC198	2.7385	4.799735	1.74122	2.75653	0.005842	0.033905
CCDC26	2.862702	4.323697	1.67212	2.58575	0.009717	0.046809
CCDC38	3.927124	5.310828	1.60024	3.31877	0.000904	0.009941
CCDC39	6.968384	3.943497	1.38718	2.84281	0.004472	0.0287
CCDC40	12.04572	1.189111	0.43668	2.72304	0.006468	0.036035
CCDC73	6.417565	3.072746	1.14138	2.69212	0.0071	0.038134
CCDC8	2.728013	2.611625	0.90979	2.87059	0.004097	0.027199
CCDC80	15.07819	1.70047	0.61915	2.74644	0.006025	0.034514
CCDC83	5.053416	4.229845	1.59509	2.65179	0.008007	0.041237
CCDC88A	33.68883	1.611884	0.5946	2.71088	0.00671	0.036838
CCER1	4.101121	4.904651	1.81458	2.70291	0.006873	0.037428
CCL21	7.963202	2.542123	0.81586	3.11588	0.001834	0.015984
CCNA1	3.209345	4.466085	1.55679	2.86877	0.004121	0.02729
CCNA2	8.882076	2.326819	0.72322	3.2173	0.001294	0.012746
CCND2	59.49073	2.213141	0.50078	4.41939	9.90E-06	0.000381
CCND3	118.4601	1.167515	0.23843	4.89675	9.74E-07	5.97E-05
CCNF	6.456192	2.185555	0.72379	3.01961	0.002531	0.019853
CCNG2	79.46148	1.018451	0.23474	4.33861	1.43E-05	0.000505
CCNYL1	25.42277	1.294499	0.38706	3.34447	0.000824	0.009406
CCR5AS	14.55266	4.395363	0.91183	4.82038	1.43E-06	8.19E-05
CD160	1.875159	3.084072	1.20951	2.54986	0.010777	0.049979
CD163	41.85339	1.325892	0.39409	3.36446	0.000767	0.008985
CD226	39.35335	1.639467	0.55102	2.97534	0.002927	0.021804
CD244	2.592711	4.180759	1.42071	2.94273	0.003253	0.023366
CD28	6.803692	2.738127	0.84925	3.22418	0.001263	0.012569
CD36	31.77893	1.362822	0.51925	2.6246	0.008675	0.043434
CD4	6.885822	1.995856	0.77921	2.56139	0.010425	0.049124
CD40	3.580385	4.128294	1.40011	2.94856	0.003193	0.023129
CD6	2.025318	3.876191	1.23285	3.14408	0.001666	0.015044
CD68	37.29286	2.177919	0.34801	6.25825	3.89E-10	8.08E-08
CD7	1.647654	3.597502	1.35849	2.64816	0.008093	0.041497
CD74	83.04709	1.612461	0.57684	2.79534	0.005184	0.031471

CD82	16.3162	1.311625	0.4101	3.19829	0.001382	0.013343
CD84	11.12721	2.04212	0.59955	3.40607	0.000659	0.008171
CD96	8.469382	2.075814	0.73481	2.82497	0.004729	0.029665
CDC14B	31.26172	1.041593	0.27339	3.80987	0.000139	0.002796
CDC45	3.337346	3.53994	1.3509	2.62044	0.008782	0.043873
CDCA2	4.537105	2.295628	0.69977	3.28056	0.001036	0.010905
CDCA7L	13.08996	1.747571	0.53129	3.2893	0.001004	0.010683
CDH12	5.45221	4.809761	1.55237	3.09833	0.001946	0.016607
CDH18	9.36907	5.15569	1.51508	3.40291	0.000667	0.008219
CDH19	11.35554	4.688679	1.35802	3.45258	0.000555	0.007328
CDH20	2.419924	4.097299	1.59669	2.56612	0.010284	0.048731
CDH26	5.553148	3.237372	1.1593	2.79252	0.00523	0.031654
CDH7	4.988729	4.667519	1.41266	3.30406	0.000953	0.010278
CDK1	6.695123	2.654731	0.82985	3.19904	0.001379	0.013325
CDKL1	4.991398	3.315916	1.10154	3.01024	0.00261	0.020164
CDKN2A	5.741131	2.585489	0.87138	2.96713	0.003006	0.022259
CDKN2C	11.04264	1.153389	0.4437	2.59946	0.009337	0.045743
CDX1	1.611658	3.652684	1.31332	2.78125	0.005415	0.032449
CEACAM19	43.68423	2.204898	0.36222	6.08722	1.15E-09	2.04E-07
CENPE	15.53234	3.222306	0.9124	3.53168	0.000413	0.006017
CENPF	17.33347	2.489089	0.65885	3.77791	0.000158	0.003077
CENPK	9.825558	3.234049	0.90896	3.55797	0.000374	0.005605
CENPW	9.234408	2.756433	0.89448	3.08159	0.002059	0.017194
CEP126	8.754227	4.545121	1.21016	3.7558	0.000173	0.003286
CEP250	27.50697	1.171033	0.33423	3.50368	0.000459	0.006423
CEP70	29.20617	1.004095	0.34347	2.92341	0.003462	0.024263
CEP72	8.96956	3.840513	0.83536	4.59745	4.28E-06	0.000194
CERK	21.54143	2.381917	0.507	4.6981	2.63E-06	0.00013
CERKL	7.672792	6.364006	2.23403	2.84866	0.00439	0.028324
CERS4	16.52113	1.109382	0.37628	2.94832	0.003195	0.023132
CFAP206	9.320007	2.254975	0.70971	3.17733	0.001486	0.013963
CFAP20DC	5.041345	4.701395	1.37142	3.42813	0.000608	0.007731
CFAP47	14.06746	3.348249	0.98425	3.40183	0.000669	0.008231
CFAP54	17.36037	3.378031	1.07024	3.15634	0.001598	0.014634
CFAP61	5.334187	5.291497	1.65758	3.19231	0.001411	0.013526
CFAP69	11.9681	4.240277	1.18052	3.59188	0.000328	0.005089
CFAP91	7.106653	3.373215	1.29453	2.60574	0.009168	0.045214
CFH	12.41166	3.612097	1.00789	3.58381	0.000339	0.005214
CFHR4	3.604497	5.248677	1.95256	2.6881	0.007186	0.03833
CFI	4.596852	4.54794	1.45443	3.12697	0.001766	0.015638
CFTR	20.07958	1.940501	0.69355	2.79792	0.005143	0.031319
CGB3	264.2484	2.169382	0.51973	4.17403	2.99E-05	0.000878
CGB5	617.0299	1.797135	0.50175	3.58175	0.000341	0.005242
CGB7	137.7987	2.033736	0.45632	4.4568	8.32E-06	0.000334

CGB8	406.2218	1.795309	0.62467	2.87403	0.004053	0.027041
CHI3L2	10.75909	2.590945	0.81282	3.18762	0.001434	0.01365
CHL1	15.45352	3.768654	1.18392	3.18321	0.001457	0.013786
CHMP1B2P	3.276338	4.56609	1.60775	2.84005	0.004511	0.028869
CHP2	3.638483	2.298583	0.89465	2.56927	0.010191	0.048481
CHRM2	5.823186	4.035836	1.49501	2.69954	0.006944	0.037632
CHST8	3.358799	3.541661	1.3845	2.55808	0.010525	0.049364
CHST9	7.114063	5.740529	1.68023	3.41652	0.000634	0.007999
CHSY3	4.522077	4.01858	1.36079	2.95312	0.003146	0.022909
CIT	8.936662	2.246502	0.67505	3.32788	0.000875	0.009723
CLC	10.22743	2.415371	0.64466	3.74671	0.000179	0.003376
CLCA3P	5.47595	5.841451	1.54074	3.79134	0.00015	0.002967
CLCA4	5.730393	4.416186	1.39146	3.17377	0.001505	0.01407
CLDN1	478.0734	-2.17878	0.46612	-4.6743	2.95E-06	0.000142
CLDN16	55.14976	-2.2608	0.43894	-5.1506	2.60E-07	2.04E-05
CLDN9	8.834476	2.872014	0.73424	3.91152	9.17E-05	0.002085
CLEC12A	11.97206	3.246576	1.19263	2.7222	0.006485	0.036078
CLGN	9.310862	3.616784	1.20067	3.01229	0.002593	0.020091
CLIC2	6.174037	1.699803	0.65013	2.61455	0.008935	0.044417
CLSPN	11.19008	2.21336	0.56755	3.89987	9.62E-05	0.002152
CLU	46.86309	1.601898	0.41029	3.90431	9.45E-05	0.002131
CLVS2	11.28899	5.358561	1.39412	3.84368	0.000121	0.002538
CMBL	3.455	2.419803	0.8458	2.86096	0.004224	0.027744
CNGB3	7.116759	4.233822	1.29506	3.26921	0.001078	0.011205
CNKSR2	11.44415	2.447723	0.59408	4.12017	3.79E-05	0.00105
CNN2	28.87996	1.247215	0.44249	2.81863	0.004823	0.030024
CNR1	7.081538	3.850075	1.10187	3.49412	0.000476	0.006578
CNTN4	10.01266	3.13012	0.92309	3.3909	0.000697	0.008398
CNTN6	13.06048	2.939543	1.02237	2.87521	0.004038	0.026996
CNTNAP3	8.934533	3.48075	1.2095	2.87785	0.004004	0.026826
CNTNAP3B	5.074929	4.144564	1.5001	2.76285	0.00573	0.03349
CNTNAP3P2	2.722972	4.280822	1.24656	3.4341	0.000595	0.007647
CNTNAP4	11.25258	4.946874	1.2698	3.89578	9.79E-05	0.002156
CNTRL	30.48217	1.398259	0.51614	2.70904	0.006748	0.03698
COL11A1	25.62631	3.590634	0.92559	3.87927	0.000105	0.002261
COL12A1	25.93329	2.58963	0.84725	3.05652	0.002239	0.018284
COL15A1	48.59543	1.339455	0.31059	4.31261	1.61E-05	0.000548
COL16A1	12.01299	1.867455	0.60511	3.08614	0.002028	0.017021
COL17A1	166.1392	3.32032	0.44931	7.38982	1.47E-13	7.67E-11
COL19A1	11.76611	4.753316	1.34645	3.53026	0.000415	0.006041
COL22A1	12.65609	3.004681	1.14744	2.6186	0.008829	0.044065
COL24A1	18.52263	3.39528	1.05907	3.2059	0.001346	0.013105
COL27A1	54.17852	2.34028	0.45049	5.19494	2.05E-07	1.69E-05
COL4A3	6.832665	3.56691	1.23443	2.88952	0.003858	0.026185

COL4A4	15.53171	4.429419	1.25491	3.52966	0.000416	0.00605
COL5A2	29.41374	1.850335	0.62415	2.96455	0.003031	0.022357
COL6A4P2	6.927484	3.895019	1.32283	2.94446	0.003235	0.023308
COL6A5	8.245538	4.464528	1.48828	2.9998	0.002702	0.020612
COL6A6	12.90923	3.363068	1.21718	2.763	0.005727	0.03349
COL9A1	8.482276	5.515367	2.07052	2.66376	0.007727	0.040286
COLQ	5.652409	3.259286	1.04148	3.12947	0.001751	0.015547
COPG2IT1	4.626632	4.043691	1.38404	2.92166	0.003482	0.024368
CORO2A	46.55691	1.749161	0.54824	3.1905	0.00142	0.01357
CORO2B	3.309567	5.091083	1.81313	2.80789	0.004987	0.030736
COX1	41271.98	1.145635	0.26493	4.32428	1.53E-05	0.000526
CP	10.44913	3.387855	0.85555	3.95985	7.50E-05	0.001804
CPB2	3.090083	4.99087	1.81119	2.75557	0.005859	0.033969
CPB2-AS1	3.382862	4.050748	1.48461	2.72849	0.006362	0.035753
CPNE8	25.81432	1.10653	0.42348	2.61294	0.008977	0.044586
CPO	8.682232	2.836013	0.92268	3.07369	0.002114	0.017583
CPOX	20.50118	1.191726	0.34319	3.4725	0.000516	0.006964
CPXM1	3.292883	2.243672	0.80817	2.77625	0.005499	0.032739
CPXM2	219.4957	1.308385	0.32282	4.05305	5.06E-05	0.00131
CR2	6.714991	5.144243	1.50902	3.40899	0.000652	0.008127
CRACD	119.7302	-1.76744	0.32152	-5.4971	3.86E-08	4.28E-06
CRB1	7.016352	5.672144	1.37758	4.11748	3.83E-05	0.001059
CRH	2455.313	1.67924	0.43715	3.84132	0.000122	0.002556
CRISP1	3.600069	4.651149	1.55156	2.99772	0.00272	0.020714
CRISPLD2	21.9854	1.681854	0.29923	5.62054	1.90E-08	2.35E-06
CRTAP	21.20485	1.33861	0.44718	2.99343	0.002759	0.020889
CSHL1	7252.341	-1.06234	0.2348	-4.5245	6.05E-06	0.000259
CSMD2	6.864019	4.016541	1.23012	3.26517	0.001094	0.011337
CSMD3	28.26516	5.366845	1.25631	4.27189	1.94E-05	0.000629
CST6	36.80876	1.979814	0.41016	4.82692	1.39E-06	8.01E-05
CTCFL	2.575634	4.142612	1.47815	2.80256	0.00507	0.031077
CTH	2.807843	3.751431	1.33113	2.81823	0.004829	0.030045
CTIF	15.11501	1.29571	0.3154	4.10816	3.99E-05	0.001087
CTNNA3	14.62931	3.195149	1.08667	2.94032	0.003279	0.023486
CTNND2	9.142471	3.165498	1.00279	3.15669	0.001596	0.014634
CTSC	23.17167	1.917618	0.55595	3.44927	0.000562	0.0074
CTSD	262.9859	1.072823	0.25101	4.27403	1.92E-05	0.000626
CTSV	5.028902	2.461348	0.809	3.04247	0.002346	0.018892
CTTNBP2	8.900122	5.062282	1.32046	3.83372	0.000126	0.00261
CTXN2	2.598127	3.624725	1.39365	2.60088	0.009299	0.045596
CUBN	17.13048	5.03037	1.16721	4.30976	1.63E-05	0.000553
CUEDC1	10.67649	1.045997	0.37519	2.78789	0.005305	0.031959
CWH43	3.635078	4.681915	1.55246	3.01581	0.002563	0.019978
CXADR	6.440938	4.08251	1.33168	3.06569	0.002172	0.017877

CXCL10	15.10448	4.530342	1.62331	2.7908	0.005258	0.031765
CXCL13	2.502874	3.576153	1.35387	2.64143	0.008256	0.041919
CXCR2P1	19.07745	1.694659	0.54224	3.12531	0.001776	0.0157
CXXC4	7.78344	3.774785	1.34956	2.79705	0.005157	0.03138
CYBB	25.80338	1.371212	0.47506	2.88643	0.003896	0.026301
CYBRD1	10.84313	2.580116	0.81065	3.18279	0.001459	0.013786
CYLC2	7.575027	5.326268	1.60737	3.31366	0.000921	0.010059
CYP2J2	28.80819	1.171869	0.30546	3.83638	0.000125	0.002591
CYP3A5	5.395597	4.801055	1.54484	3.1078	0.001885	0.016286
CYP4F11	2.634588	4.724682	1.7989	2.62643	0.008629	0.043309
CYTH4	8.720185	1.616008	0.55079	2.93398	0.003346	0.023842
CYYR1	12.98829	2.551238	0.75699	3.37025	0.000751	0.008843
DAPK2	5.061283	3.516373	1.16322	3.02296	0.002503	0.019702
DARS1-AS1	5.213694	2.127678	0.76643	2.77607	0.005502	0.032739
DAZL	11.44108	1.910866	0.60278	3.17006	0.001524	0.014193
DBF4	16.96534	1.194744	0.42422	2.81632	0.004858	0.030159
DBF4B	7.966458	1.863559	0.55471	3.35953	0.000781	0.009107
DCAF13P3	3.393363	4.017081	1.19887	3.35073	0.000806	0.009263
DCC	9.973058	4.144518	1.34435	3.08293	0.00205	0.017144
DCDC2	7.008835	2.811791	1.02007	2.75648	0.005843	0.033905
DCHS2	10.02952	4.536239	1.34709	3.36743	0.000759	0.008908
DCLK1	10.91502	3.025545	0.90145	3.3563	0.00079	0.009174
DCN	40.48746	1.333172	0.4405	3.02648	0.002474	0.019522
DDAH1	18.95645	1.315773	0.41998	3.13295	0.001731	0.015413
DDIT4	18.85625	1.572291	0.38856	4.04642	5.20E-05	0.001341
DDIT4L	24.45308	2.601455	0.5238	4.96655	6.82E-07	4.39E-05
DDR2	32.46111	1.249912	0.41295	3.02676	0.002472	0.019522
DDX11L10	5.216543	2.773004	0.85271	3.25199	0.001146	0.011697
DDX4	4.409386	5.010683	1.46328	3.42429	0.000616	0.007816
DELEC1	2.550901	4.155223	1.62638	2.5549	0.010622	0.049574
DENND2A	15.78645	1.384628	0.43346	3.19438	0.001401	0.013453
DEPDC1	9.743774	3.100252	0.99571	3.11361	0.001848	0.016047
DEPP1	955.371	-1.00014	0.16016	-6.2447	4.25E-10	8.69E-08
DEUP1	6.385184	5.562546	1.67752	3.31593	0.000913	0.010006
DGKB	14.74927	4.178755	1.21283	3.44546	0.00057	0.007457
DGKD	34.98655	1.14281	0.35508	3.21845	0.001289	0.012729
DGKE	7.656745	2.481439	0.74333	3.33829	0.000843	0.009501
DIAPH3	34.66646	1.475672	0.54677	2.69888	0.006957	0.037644
DIO2	258.6338	4.674632	0.63124	7.40543	1.31E-13	7.05E-11
DIPK2A	7.274361	1.894559	0.72176	2.62491	0.008667	0.043408
DKK1	6.202404	2.015028	0.72085	2.79534	0.005185	0.031471
DKK3	5.228432	1.634247	0.62146	2.62971	0.008546	0.042987
DLC1	73.22413	1.282218	0.32366	3.96165	7.44E-05	0.001793
DLG2	11.87315	2.9563	1.00985	2.92748	0.003417	0.024109

DLGAP1-AS5	3.196822	5.048074	1.95038	2.58825	0.009647	0.046596
DLGAP5	6.770753	2.780899	0.89302	3.11402	0.001846	0.01604
DLSTP1	3.979837	3.288329	1.19994	2.74041	0.006136	0.034932
DMTN	22.78383	1.406852	0.51589	2.72706	0.00639	0.03581
DNAAF11	5.283212	3.37996	1.27984	2.64093	0.008268	0.041967
DNAAF9	8.168299	2.365869	0.80767	2.92925	0.003398	0.024009
DNAH11	116.6455	1.231137	0.43896	2.80464	0.005037	0.030917
DNAH12	19.83938	2.926691	0.87704	3.33703	0.000847	0.009519
DNAH2	8.675409	3.966526	1.31867	3.00797	0.00263	0.020247
DNAH5	24.20238	3.902821	1.17224	3.32938	0.00087	0.009689
DNAH6	17.37316	2.132463	0.83002	2.56917	0.010194	0.048481
DNAH7	21.89462	3.280863	0.86027	3.81376	0.000137	0.002766
DNAH8	24.42053	3.781985	0.97896	3.86329	0.000112	0.002383
DNAH9	11.03675	4.987899	1.40058	3.5613	0.000369	0.00555
DNAI3	4.101464	3.404964	1.30168	2.61583	0.008901	0.044319
DNAJA4	64.64305	-1.10487	0.34399	-3.2119	0.001319	0.012905
DNAJB7	3.558899	4.659646	1.70093	2.73947	0.006154	0.034992
DNAJB9	848.4424	-1.03526	0.26876	-3.852	0.000117	0.002467
DNM1	2.300951	3.00423	1.17706	2.55231	0.010701	0.049757
DNM3	34.82663	1.726429	0.50275	3.43396	0.000595	0.007647
DNM3OS	7.854729	3.897147	1.18348	3.29297	0.000991	0.010586
DOCK10	19.29557	2.575622	0.68854	3.74068	0.000184	0.003426
DOCK2	17.90556	1.710481	0.59198	2.88942	0.003859	0.026185
DOCK4	14.9558	2.447873	0.62442	3.92024	8.85E-05	0.002023
DOK2	10.09551	1.989006	0.57719	3.44602	0.000569	0.007452
DOK5	2.983041	3.834972	1.47176	2.6057	0.009169	0.045214
DOK6	8.429319	3.764337	1.13809	3.30759	0.000941	0.010231
DPF3	4.854036	4.644843	1.35436	3.42954	0.000605	0.007715
DPP10	14.5665	2.620634	0.89532	2.92703	0.003422	0.024117
DPP4	30.96124	1.767051	0.32926	5.36673	8.02E-08	8.00E-06
DPP6	2.837505	3.706393	1.39095	2.66464	0.007707	0.040232
DPY19L2	11.09534	1.812067	0.59491	3.04596	0.002319	0.018739
DPY19L2P2	3.520193	4.622313	1.49461	3.09264	0.001984	0.016818
DPYS	4.178842	3.885811	1.3825	2.81071	0.004943	0.030573
DPYSL2	50.29789	1.389713	0.38038	3.65352	0.000259	0.004311
DPYSL3	19.36642	2.013456	0.66751	3.01637	0.002558	0.019951
DSC1	7.359656	3.024216	1.12042	2.69919	0.006951	0.037633
DSEL-AS1	3.50928	5.186037	2.00316	2.58893	0.009628	0.046546
DSG1	8.77498	3.901972	1.07754	3.62118	0.000293	0.004692
DSG2-AS1	1.846092	4.103855	1.48722	2.75941	0.005791	0.033748
DSG3	4.40739	5.541471	1.89071	2.93089	0.00338	0.023935
DSG4	6.084998	5.99563	1.74429	3.43729	0.000588	0.00761
DSPP	4.055273	5.370812	1.6453	3.26433	0.001097	0.01136
DTHD1	5.123851	3.32657	1.17366	2.83435	0.004592	0.02909

DTL	10.07487	2.699709	0.7574	3.56446	0.000365	0.005489
DTNA	15.97272	3.567655	0.86472	4.12579	3.69E-05	0.001033
DTWD2	18.17321	1.154135	0.39775	2.90162	0.003712	0.025453
DUBR	185.6496	-1.26231	0.29194	-4.3239	1.53E-05	0.000526
DUOXA1	2.96128	3.226099	1.18893	2.71344	0.006659	0.036679
DVL1	185.9147	1.07072	0.23185	4.6181	3.87E-06	0.000178
DYDC1	4.031571	5.408675	1.88366	2.87136	0.004087	0.027179
DYNC1I1	14.5636	2.013452	0.72544	2.77549	0.005512	0.032749
DYNC2H1	44.27169	2.434203	0.71623	3.39862	0.000677	0.00828
DYNC2I1	300.7708	1.248706	0.25015	4.99193	5.98E-07	4.06E-05
DYNLT5	2.583876	4.122068	1.51069	2.7286	0.00636	0.035753
DZIP1	7.916537	1.883671	0.62349	3.02117	0.002518	0.01979
DZIP1L	6.27508	5.021464	1.48533	3.3807	0.000723	0.00861
E2F3	9.279109	1.739145	0.57638	3.01734	0.00255	0.019916
E2F7	7.212702	5.203225	1.28438	4.05115	5.10E-05	0.001316
EBF1	6.10809	2.063924	0.79568	2.59392	0.009489	0.046109
ECE1	26.40481	2.016964	0.31673	6.36806	1.91E-10	4.76E-08
EDIL3	7.320836	4.097886	1.41995	2.88594	0.003903	0.026321
EDNRA	4.594826	3.375666	1.25731	2.68484	0.007256	0.038575
EDNRB-AS1	3.559389	4.157647	1.52391	2.72827	0.006367	0.035764
EEF2K	24.47679	1.278624	0.33378	3.8307	0.000128	0.002637
EFCAB5	10.96456	3.58079	1.11812	3.20252	0.001362	0.013228
EFNA5	6.89224	2.146713	0.8139	2.63758	0.00835	0.042225
EGF	14.06133	2.543547	0.81197	3.13255	0.001733	0.015413
EGFR-AS1	10.66489	1.762951	0.60145	2.93118	0.003377	0.023923
EGLN3	51.30267	3.472306	0.42146	8.23869	1.74E-16	1.88E-13
EHF	9.252328	2.136453	0.79127	2.70003	0.006933	0.037611
EID1	370.867	-1.04769	0.24154	-4.3375	1.44E-05	0.000507
EIF3C	31.32255	-1.26327	0.34648	-3.646	0.000266	0.004389
EIF4EBP1	16.59903	1.639451	0.53629	3.05699	0.002236	0.018264
ELAVL2	5.16322	4.231976	1.55894	2.71465	0.006635	0.036558
ELAVL4	6.858515	4.645834	1.31193	3.54123	0.000398	0.005848
ELMO3	43.89582	1.015324	0.25939	3.9143	9.07E-05	0.002067
ELOVL6	6.362584	2.674349	1.02504	2.60902	0.00908	0.044976
EMBP1	3.241894	2.943674	1.14814	2.56387	0.010351	0.04893
EML5	15.08249	2.947436	1.04295	2.82607	0.004712	0.029575
EML6	11.74307	3.150452	1.02845	3.06332	0.002189	0.017973
EMP1	10.95536	1.713498	0.55534	3.08549	0.002032	0.01704
EMP3	30.67821	1.467647	0.43075	3.40719	0.000656	0.00815
EMX2OS	3.578604	2.949839	1.01531	2.90537	0.003668	0.025289
ENAH	53.46677	1.241323	0.39374	3.15267	0.001618	0.014769
ENAM	3.056956	4.408921	1.32075	3.3382	0.000843	0.009501
ENC1	12.75802	1.769712	0.52779	3.35304	0.000799	0.009229
ENG	729.0717	2.098167	0.21658	9.68781	3.40E-22	7.85E-19

ENKUR	10.44602	1.968413	0.6969	2.82451	0.004735	0.029677
ENO4	6.849123	4.630346	1.38739	3.33744	0.000846	0.009513
ENPP2	14.53178	1.661249	0.6173	2.69114	0.007121	0.038158
ENPP3	5.524869	3.801023	1.2267	3.09857	0.001945	0.016607
ENTHD1	4.997915	4.224156	1.50491	2.80691	0.005002	0.030788
ENTPD5	20.32028	1.118251	0.35414	3.15766	0.00159	0.014623
EPGN	6.094316	4.265051	1.36682	3.12041	0.001806	0.015825
EPHA3	7.454402	3.426072	1.20237	2.84944	0.00438	0.028288
EPHA4	8.11622	4.413581	1.40299	3.14585	0.001656	0.014982
EPHA5	19.38234	5.58443	1.77391	3.14808	0.001643	0.014944
EPHA6	11.55211	5.52883	2.14134	2.58195	0.009824	0.04717
EPHB1	4.627378	3.346427	1.31252	2.54963	0.010784	0.049983
ERBB4	21.53229	2.932423	0.67828	4.32334	1.54E-05	0.000526
EREG	5.230684	4.22836	1.39073	3.04039	0.002363	0.018994
ERGIC1	438.2953	1.135932	0.22575	5.03184	4.86E-07	3.52E-05
ERICH3	17.84242	4.443028	1.1361	3.91079	9.20E-05	0.002088
ERICH5	59.71107	-1.40307	0.42574	-3.2956	0.000982	0.010508
ERMN	4.870578	4.618512	1.49659	3.08603	0.002028	0.017021
ERO1A	229.8484	1.206861	0.24222	4.98247	6.28E-07	4.18E-05
ERV3-1	287.7405	-1.07788	0.3198	-3.3705	0.00075	0.008843
ESCO2	5.634901	3.002075	1.11125	2.70152	0.006902	0.037572
ESPL1	2.795737	3.245203	1.13431	2.86096	0.004224	0.027744
ESR1	9.282391	1.409282	0.54255	2.59754	0.00939	0.045861
ESYT1	32.03095	1.826512	0.2623	6.96334	3.32E-12	1.38E-09
ETNPPL	2.933701	4.879342	1.28803	3.78823	0.000152	0.002991
ETS1	44.33745	1.56667	0.49012	3.19649	0.001391	0.013395
ETV1	10.7624	4.97269	1.29676	3.8347	0.000126	0.002602
EVC	7.903738	2.501631	0.8904	2.80955	0.004961	0.030648
EVC2	5.597888	2.956927	0.83314	3.54914	0.000386	0.005738
EXT1	38.17605	1.103491	0.31788	3.47139	0.000518	0.006982
EXTL3	12.84218	1.148546	0.38164	3.00954	0.002616	0.020187
EYA1	9.392966	3.435319	1.08797	3.15755	0.001591	0.014623
EYA2	4.570929	2.468462	0.86629	2.84947	0.004379	0.028288
EYS	16.84134	2.373517	0.87896	2.70037	0.006926	0.037605
F13B	4.227122	3.837724	1.46931	2.61192	0.009003	0.044705
F2R	9.794902	1.502532	0.57961	2.59233	0.009533	0.046184
F8	10.25334	1.429401	0.51598	2.77027	0.005601	0.033059
FABP5	5.726979	1.787494	0.66461	2.68955	0.007155	0.038276
FABP7	4.262136	3.890996	1.45829	2.6682	0.007626	0.039973
FAM110A	32.53833	1.007991	0.2861	3.52322	0.000426	0.006121
FAM131B	3.92604	4.287599	1.182	3.62742	0.000286	0.004647
FAM171B	635.2447	-1.02532	0.2048	-5.0066	5.54E-07	3.81E-05
FAM177B	4.932238	4.18004	1.50839	2.77119	0.005585	0.033003
FAM184A	144.0229	1.229094	0.25537	4.813	1.49E-06	8.35E-05

FAM222A	3.415967	2.721366	0.84993	3.20188	0.001365	0.013249
FAM227B	13.18466	2.835458	0.92837	3.05423	0.002256	0.018383
FAM229B	3.903841	4.285171	1.29184	3.31712	0.00091	0.009976
FAM53B	41.25671	1.201597	0.30891	3.88982	0.0001	0.00219
FAM78A	11.21044	1.720041	0.6389	2.69219	0.007098	0.038134
FANCI	22.6799	1.751216	0.44328	3.95056	7.80E-05	0.001843
FANK1	19.72872	1.321147	0.34142	3.86955	0.000109	0.002338
FAR1	26.19428	1.012151	0.35669	2.83759	0.004546	0.028985
FAS	7.608428	3.012329	1.00565	2.99539	0.002741	0.020824
FAT3	9.795956	2.937711	0.88833	3.307	0.000943	0.010239
FAT4	20.61028	2.851722	0.85427	3.33822	0.000843	0.009501
FBXO43	3.068973	3.961384	1.52472	2.59811	0.009374	0.045826
FBXO5	27.56846	-1.05376	0.26668	-3.9514	7.77E-05	0.001843
FCER1G	11.06733	1.62526	0.51403	3.16181	0.001568	0.014485
FCGR3A	18.91757	1.763105	0.65151	2.70616	0.006807	0.037201
FCHO1	6.532729	2.440026	0.71435	3.41575	0.000636	0.008012
FCMR	5.244408	1.837402	0.67019	2.74162	0.006114	0.034837
FCRL1	4.847434	4.640446	1.32607	3.4994	0.000466	0.006482
FCRL2	4.479951	5.02266	1.62542	3.09007	0.002001	0.01692
FCRL5	2.360981	4.011128	1.45214	2.76222	0.005741	0.033531
FCRLB	3.445919	2.211716	0.77384	2.8581	0.004262	0.027854
FERMT1	12.02304	2.141859	0.79605	2.6906	0.007132	0.038207
FGF14	4.373722	3.176039	1.18116	2.68891	0.007169	0.038322
FGF14-IT1	4.818352	4.635734	1.67925	2.76061	0.005769	0.033649
FGF23	4.647635	3.991769	1.47655	2.70345	0.006862	0.037393
FGF5	3.652803	5.257224	1.91289	2.74831	0.00599	0.034428
FGF7	7.573446	3.273689	0.97632	3.35309	0.000799	0.009229
FGFR1	25.88386	1.267957	0.34302	3.6965	0.000219	0.003845
FGGY	31.92696	1.129202	0.28346	3.98358	6.79E-05	0.001673
FHAD1	2.858564	4.342787	1.62437	2.67351	0.007506	0.039567
FHL2	135.0662	1.008951	0.23576	4.27957	1.87E-05	0.000614
FHL5	5.15526	3.333735	0.97912	3.40484	0.000662	0.008186
FILIP1	9.989195	2.964651	0.81521	3.63667	0.000276	0.004519
FKBP5	14.22509	1.950577	0.54398	3.58576	0.000336	0.005185
FLG2	4.923687	3.786788	1.36904	2.76602	0.005674	0.033351
FLG-AS1	3.297688	2.997322	1.09732	2.7315	0.006305	0.035552
FLJ46284	4.194198	4.936149	1.85701	2.65811	0.007858	0.040796
FLNA	204.5374	1.361495	0.34265	3.97339	7.09E-05	0.001728
FLNB	244.1072	1.930624	0.20715	9.31984	1.17E-20	2.35E-17
FLT1	6013.279	3.071992	0.28287	10.8601	1.78E-27	7.21E-24
FMO1	2.533398	4.713763	1.75328	2.68854	0.007177	0.038325
FMO9P	3.095158	5.005898	1.92624	2.59879	0.009355	0.045768
FN1	2594.044	1.652523	0.2121	7.79113	6.64E-15	5.37E-12
FOLH1	8.089883	3.751306	1.17464	3.19358	0.001405	0.013483

FOLH1B	5.35787	4.326864	1.52477	2.83771	0.004544	0.028985
FOLR1	64.72161	-1.01383	0.30182	-3.359	0.000782	0.009112
FPGS	13.27364	1.664209	0.46143	3.60665	0.00031	0.004892
FPR3	5.812207	2.666854	0.84163	3.16867	0.001531	0.014221
FREM1	14.31978	5.762693	1.32493	4.34943	1.36E-05	0.000488
FREM2	18.23772	2.540209	0.68646	3.70046	0.000215	0.003811
FRMD3	14.56811	1.568137	0.55325	2.83439	0.004591	0.02909
FRMD4A	78.31831	1.969652	0.25362	7.7662	8.09E-15	5.94E-12
FRRS1	13.45096	1.445527	0.47856	3.0206	0.002523	0.019818
FRZB	595.8459	-1.65351	0.33867	-4.8824	1.05E-06	6.37E-05
FSD1L	8.762775	3.185885	1.01543	3.13747	0.001704	0.015272
FSD2	3.365585	4.570079	1.6636	2.74711	0.006012	0.034488
FSHR	3.372827	5.126925	1.65327	3.10109	0.001928	0.01651
FSIP2	27.12892	1.987662	0.6834	2.90847	0.003632	0.025156
FSTL3	1059.991	4.978037	0.63496	7.83988	4.51E-15	3.84E-12
FTH1	1248.719	1.502903	0.28142	5.34037	9.28E-08	9.04E-06
FTSJ1	8.651982	1.476715	0.52094	2.83473	0.004587	0.02909
FUT9	7.506996	2.629867	1.02041	2.57727	0.009959	0.047591
FXYD3	292.7858	-1.18196	0.35502	-3.3292	0.000871	0.009689
FYN	22.04016	1.377184	0.42485	3.24155	0.001189	0.01199
FZD3	27.55555	1.49796	0.57239	2.61702	0.00887	0.04422
FZD5	10.01171	1.33322	0.4795	2.78045	0.005428	0.032492
GABRA1	5.465315	5.319562	1.56068	3.40848	0.000653	0.008127
GABRB2	8.963195	5.120823	1.61224	3.17623	0.001492	0.013992
GABRG1	10.22365	5.766944	1.45104	3.97436	7.06E-05	0.001724
GABRG2	4.23998	4.930526	1.40025	3.52118	0.00043	0.006158
GABRP	3.8077	4.262979	1.15024	3.70617	0.00021	0.003742
GABRR1	29.96968	-1.08161	0.33397	-3.2386	0.001201	0.012068
GAD2	4.106888	4.897808	1.8309	2.67508	0.007471	0.039451
GALK1	24.59825	1.098947	0.36397	3.0193	0.002534	0.019855
GALNT13	10.24772	3.764477	1.08602	3.46631	0.000528	0.00705
GALNT15	6.229543	1.83687	0.60409	3.04072	0.00236	0.018983
GALNT18	11.72154	1.91084	0.74412	2.56792	0.010231	0.048534
GALNT6	18.04335	1.100707	0.36104	3.04867	0.002299	0.018617
GAPDH	3065.774	3.261959	0.28898	11.2879	1.51E-29	1.22E-25
GARNL3	8.134765	1.929859	0.6778	2.84722	0.00441	0.028441
GAS2	9.194128	3.966565	1.47053	2.69737	0.006989	0.037739
GAS5	85.02489	1.196944	0.19992	5.98719	2.13E-09	3.71E-07
GASK1B	7.104422	1.644175	0.56578	2.90605	0.00366	0.025256
GBAP1	3.32055	2.657195	1.01616	2.61495	0.008924	0.044389
GBGT1	55.88299	1.339489	0.33329	4.01901	5.84E-05	0.001474
GBP1	81.4732	1.480384	0.27193	5.44401	5.21E-08	5.50E-06
GBP4	30.15013	2.141379	0.69821	3.06695	0.002163	0.01782
GBP5	17.3633	3.157461	0.82153	3.84338	0.000121	0.002538

GC	2.427991	4.636134	1.69083	2.74193	0.006108	0.034837
GCLM	217.1623	-1.04211	0.2272	-4.5868	4.50E-06	0.000201
GCNT2	18.281	1.546494	0.58236	2.65558	0.007917	0.040963
GDF11	4.698917	4.542163	1.21207	3.74746	0.000179	0.00337
GDNF-AS1	4.733653	4.62661	1.66503	2.77869	0.005458	0.032572
GFOD1	15.97401	2.710635	0.60497	4.48062	7.44E-06	0.000307
GH1	22.43087	-3.50627	0.88264	-3.9725	7.11E-05	0.001732
GIMAP1	7.417505	2.055395	0.74391	2.76297	0.005728	0.03349
GIMAP4	18.96678	2.278114	0.5842	3.89952	9.64E-05	0.002152
GIMAP7	9.947236	2.161853	0.73459	2.94295	0.003251	0.023363
GINS1	8.232621	2.471237	0.6859	3.60289	0.000315	0.004949
GLRA3	6.86559	4.165303	1.47658	2.8209	0.004789	0.029916
GLRB	3.750025	4.747499	1.62278	2.92553	0.003439	0.024189
GLT1D1	4.192045	2.875996	1.12582	2.55458	0.010632	0.049588
GLT8D2	3.673016	2.243617	0.87942	2.55124	0.010734	0.049868
GLYATL1	3.67376	4.685807	1.54131	3.04014	0.002365	0.019001
GLYATL3	3.952386	3.736056	1.44006	2.59437	0.009477	0.046085
GMDS	14.28926	1.011741	0.39387	2.56874	0.010207	0.048502
GML	1.449508	3.948196	1.5028	2.62723	0.008608	0.043242
GNA12	97.42583	1.181079	0.27178	4.34575	1.39E-05	0.000493
GNAL	4.251577	2.590374	1.00454	2.57867	0.009918	0.047427
GNB4	43.49711	1.19403	0.4009	2.97834	0.002898	0.021642
GNG2	15.72896	2.013343	0.68127	2.95526	0.003124	0.022781
GNLY	14.01959	2.771119	0.6382	4.34207	1.41E-05	0.0005
GNRHR	3.652225	5.242412	2.02092	2.59407	0.009485	0.046103
GOLGA2P6	2.850696	4.813914	1.64774	2.92152	0.003483	0.024368
GOLGA6L2	3.834716	4.792549	1.6208	2.9569	0.003107	0.022703
GPAM	10.54784	1.473453	0.50469	2.91953	0.003506	0.024503
GPC4	26.28492	1.645749	0.48948	3.36227	0.000773	0.009037
GPC5	12.1409	3.543162	1.04986	3.37488	0.000738	0.008753
GPM6A	6.802018	4.123532	1.25477	3.28627	0.001015	0.01076
GPR157	226.5967	1.338257	0.26652	5.02131	5.13E-07	3.62E-05
GPR17	2.503221	3.257842	1.04841	3.10742	0.001887	0.01629
GPR21	10.947	3.132702	0.9876	3.17203	0.001514	0.014146
GPR32	2.724582	3.678338	1.07837	3.41103	0.000647	0.008116
GPR85	2.875844	4.375076	1.58902	2.75331	0.0059	0.034087
GPRC5D-AS1	60.54943	-1.03865	0.23039	-4.5081	6.54E-06	0.000276
GPRIN2	5.081007	4.659045	1.42098	3.27875	0.001043	0.010953
GPRIN3	14.17173	3.167811	0.85193	3.71838	0.000201	0.003626
GPT2	55.71807	1.394221	0.29994	4.64833	3.35E-06	0.000158
GPX3	135.0359	2.195919	0.31582	6.953	3.58E-12	1.41E-09
GRAP	5.841067	1.931293	0.69847	2.76505	0.005691	0.033387
GRB14	33.12811	1.151266	0.29178	3.9457	7.96E-05	0.001872
GREB1	7.185938	3.418822	0.95696	3.57257	0.000353	0.005366

GRHL3	9.608464	1.277475	0.46923	2.7225	0.006479	0.036069
GRIA1	7.723952	3.859198	1.2618	3.05849	0.002225	0.01821
GRIA2	11.19284	4.953973	1.40927	3.51529	0.000439	0.006246
GRIA3	3.297493	4.568905	1.61602	2.82726	0.004695	0.029556
GRID2	8.673002	3.738417	1.23589	3.02487	0.002487	0.019597
GRIK1	3.521368	3.173854	1.17641	2.69792	0.006977	0.037707
GRIN2A	10.05159	5.17977	1.41638	3.65704	0.000255	0.004275
GRIN2B	24.89666	4.126018	1.24262	3.32042	0.000899	0.009912
GRIN3A	7.189129	3.441128	1.24889	2.75535	0.005863	0.033973
GRM3	4.544334	4.528183	1.5966	2.83615	0.004566	0.029045
GRM5	7.413575	3.577747	1.33065	2.68871	0.007173	0.038322
GRM5P1	2.705507	4.218813	1.4451	2.91939	0.003507	0.024503
GRM8	8.693057	3.494158	0.99054	3.52752	0.000419	0.006063
GSAP	63.61118	1.06758	0.28966	3.68565	0.000228	0.003957
GSDMC	4.19289	4.402136	1.47775	2.97895	0.002892	0.021608
GSTA3	276.767	-1.3245	0.26469	-5.004	5.62E-07	3.84E-05
GTSE1	4.802129	3.588711	0.9243	3.88263	0.000103	0.002239
GUCY1A2	26.65364	1.889061	0.70781	2.66888	0.00761	0.039922
GUCY2EP	4.53273	4.007611	1.07674	3.72197	0.000198	0.003588
GYPB	5.11152	3.422641	1.1072	3.09126	0.001993	0.01687
H1-5	14.28542	1.503063	0.44912	3.3467	0.000818	0.009357
H3C2	14.20294	1.534904	0.57825	2.65441	0.007945	0.041075
H4C1	3.664647	1.763927	0.68133	2.58895	0.009627	0.046546
HAS2	3.577942	5.186734	1.47622	3.51352	0.000442	0.00626
HCAR1	2.174662	3.0363	1.15047	2.63919	0.00831	0.042098
HCN1	7.957561	3.677695	1.19692	3.07262	0.002122	0.017637
HDAC9	30.64232	1.896499	0.59097	3.20914	0.001331	0.013005
HDX	6.969575	4.095554	1.39903	2.92742	0.003418	0.024109
HECW1	5.233125	4.766954	1.49648	3.18544	0.001445	0.013697
HELZ2	8.539948	1.786079	0.56948	3.13632	0.001711	0.01529
HERC3	38.65501	1.430785	0.44831	3.1915	0.001415	0.013555
HEY1	63.70949	1.305834	0.30389	4.2971	1.73E-05	0.000579
HFE	13.12337	1.659731	0.38899	4.26677	1.98E-05	0.000639
HIP1	16.88519	1.49252	0.55797	2.6749	0.007475	0.039455
HK2	116.757	2.875066	0.4903	5.86385	4.52E-09	6.59E-07
HLA-A	11.12855	2.588241	0.98358	2.63146	0.008502	0.042806
HLF	859.9394	-1.31524	0.25239	-5.2112	1.88E-07	1.60E-05
HLTF	17.52922	2.524849	0.59819	4.22083	2.43E-05	0.000748
HMCN1	27.69698	2.657325	0.82733	3.21194	0.001318	0.012905
HMGA2	2.723156	4.175875	1.36745	3.05377	0.00226	0.018387
HMGA2-AS1	6.095404	5.489942	1.6411	3.34527	0.000822	0.009388
HNF4G	7.88619	3.206353	1.15746	2.77016	0.005603	0.033059
HNRNPA3P1	2.749219	3.713708	1.39987	2.65289	0.007981	0.041181
HOXB3	7.823457	4.596696	1.46741	3.13252	0.001733	0.015413

HPCAL1	549.4147	1.298212	0.32578	3.9849	6.75E-05	0.001669
HPSE2	6.779583	5.144663	1.40584	3.65951	0.000253	0.004247
HS6ST1	23.35621	1.062064	0.29258	3.62995	0.000283	0.004611
HS6ST3	9.050199	4.370867	1.23424	3.54133	0.000398	0.005848
HSD3BP4	6.917823	1.750443	0.65696	2.66447	0.007711	0.04024
HSPA2	74.50248	1.35697	0.16411	8.2688	1.35E-16	1.59E-13
HSPG2	140.6227	1.099084	0.18779	5.85282	4.83E-09	6.91E-07
HTR1A	3.819273	4.262692	1.60724	2.65218	0.007998	0.041203
HTR1F	5.512796	3.758784	1.38544	2.71307	0.006666	0.036696
HTR2A	3.382979	4.037266	1.28618	3.13897	0.001695	0.015236
HTR2C	2.061881	4.34264	1.59779	2.7179	0.00657	0.036312
HTRA1	3893.187	1.695119	0.21862	7.75369	8.93E-15	6.27E-12
HTRA4	423.7306	4.232474	0.38547	10.9801	4.76E-28	2.57E-24
HUNK	9.331799	1.909826	0.59133	3.22974	0.001239	0.01238
HYDIN	10.28463	2.369746	0.86544	2.73821	0.006178	0.035085
HYDIN2	4.477459	3.247829	1.1604	2.79889	0.005128	0.031272
HYMAI	3.246609	3.921479	1.35694	2.88994	0.003853	0.026185
IAPP	4.453385	4.520814	1.68529	2.68251	0.007307	0.038809
ICOS	6.296699	5.015758	1.33063	3.76946	0.000164	0.00316
IDH1	101.8145	-1.85279	0.35702	-5.1896	2.11E-07	1.72E-05
IFI16	16.5905	1.622427	0.54439	2.98025	0.00288	0.021547
IFI44	7.144509	2.530206	0.79343	3.18896	0.001428	0.013603
IFI44L	26.50697	2.826671	0.58254	4.85233	1.22E-06	7.20E-05
IFIT1	11.67811	2.550655	0.53749	4.74549	2.08E-06	0.00011
IFIT3	18.02606	2.132367	0.48257	4.41877	9.93E-06	0.000381
IFITM2	66.52956	-1.68399	0.27598	-6.1018	1.05E-09	1.90E-07
IFITM3	170.6223	-1.29012	0.28641	-4.5045	6.65E-06	0.00028
IFT172	6.430457	2.899875	1.02581	2.8269	0.0047	0.029558
IGDCC3	8.61008	1.130406	0.4176	2.70688	0.006792	0.037146
IGF1	14.50734	2.032856	0.64008	3.17593	0.001494	0.013998
IGF2	196.6819	2.432283	0.34495	7.05112	1.77E-12	7.55E-10
IGF2BP1	7.88694	3.026238	0.92833	3.25988	0.001115	0.011485
IGFBP3	101.6625	2.401516	0.41094	5.84389	5.10E-09	7.17E-07
IGFBP4	34.7611	1.347549	0.37438	3.59941	0.000319	0.004992
IGFBP5	38.37669	1.362738	0.42321	3.21997	0.001282	0.012703
IGFL2	3.257238	4.063327	1.37274	2.96001	0.003076	0.022555
IGFL2-AS1	34.80703	2.099123	0.51693	4.06075	4.89E-05	0.00128
IGHA1	18.78782	-1.89038	0.61897	-3.0541	0.002257	0.018383
IGSF1	3.613903	2.866388	1.07413	2.66856	0.007618	0.039947
IGSF8	41.82189	1.856941	0.37877	4.90253	9.46E-07	5.83E-05
IKZF3	34.98379	1.831124	0.56958	3.21489	0.001305	0.012833
IL10RA	11.14246	2.209489	0.80973	2.72868	0.006359	0.035753
IL16	11.99378	2.276487	0.63833	3.56633	0.000362	0.00546
IL18R1	5.88619	3.185189	1.22274	2.60495	0.009189	0.045251

IL1RL1	8.539424	2.033497	0.73368	2.77163	0.005578	0.032983
IL2RB	73.65029	1.226245	0.31266	3.92199	8.78E-05	0.002011
IL32	24.131	1.478739	0.53075	2.78613	0.005334	0.032082
IL7	16.10301	5.273759	1.25561	4.20014	2.67E-05	0.000797
INHA	364.4699	1.906608	0.21356	8.92754	4.36E-19	5.87E-16
INHBA	1480.171	1.601506	0.2225	7.1979	6.11E-13	2.75E-10
INHBA-AS1	24.54074	1.436883	0.34036	4.22164	2.43E-05	0.000747
INPP4B	22.36211	1.410329	0.53216	2.65022	0.008044	0.04135
INSC	4.683167	5.60013	1.77955	3.14694	0.00165	0.01496
INTU	10.00953	2.178853	0.81358	2.67809	0.007404	0.039179
IPMK	130.5963	1.105783	0.29065	3.80452	0.000142	0.002839
IPW	8.164039	3.597897	1.35445	2.65636	0.007899	0.040904
IQCA1	4.299804	4.973094	1.6196	3.07057	0.002137	0.017686
IQCH	9.476638	1.907849	0.74573	2.55836	0.010517	0.049364
IRAG2	10.75865	2.238654	0.74656	2.99863	0.002712	0.020672
IRAK3	10.15722	2.523995	0.63991	3.94431	8.00E-05	0.001878
IRF8	18.70334	1.74395	0.48481	3.59717	0.000322	0.00502
IRS1	31.85802	1.213598	0.36196	3.35287	0.0008	0.009229
IRS4	2.449946	4.671298	1.68998	2.76411	0.005708	0.033446
IRX2	6.384401	1.814899	0.59104	3.0707	0.002136	0.017686
ISG15	31.88346	2.02859	0.38663	5.2468	1.55E-07	1.35E-05
ISL1	5.863082	2.727376	1.00269	2.72006	0.006527	0.036216
ISL1-DT	5.183531	3.123858	1.16199	2.68837	0.00718	0.038325
ISYNA1	106.9871	1.527049	0.42203	3.61832	0.000297	0.004728
ITGA10	4.251541	3.892955	1.39362	2.79342	0.005215	0.031591
ITGA2	8.454821	3.190664	0.93959	3.39581	0.000684	0.008307
ITGA4	13.29177	3.069287	0.86459	3.54997	0.000385	0.005738
ITGA6	29.79405	1.229781	0.45452	2.70569	0.006816	0.037242
ITGA8	12.97161	5.629875	1.31359	4.28586	1.82E-05	0.000599
ITGAD	3.644865	2.859509	1.07559	2.65855	0.007848	0.040756
ITGB2	20.71886	1.851375	0.6676	2.77318	0.005551	0.032874
ITGB8	35.69142	1.664488	0.40164	4.14428	3.41E-05	0.000969
ITGBL1	10.6415	3.004464	0.94301	3.18603	0.001442	0.013688
ITIH2	3.446705	5.167995	1.94357	2.65903	0.007837	0.040712
ITK	7.021058	2.283551	0.86739	2.63267	0.008472	0.04272
ITLN1	2.042481	3.83294	1.35545	2.82779	0.004687	0.02953
ITM2A	5.122292	3.71144	1.21761	3.04814	0.002303	0.018641
ITPR3	42.64349	1.216087	0.45578	2.66812	0.007628	0.039973
IYD	4.834867	3.259429	1.15989	2.81012	0.004952	0.030617
JAKMIP2	8.387644	3.49432	1.21322	2.8802	0.003974	0.026672
JPH2	30.8866	4.494562	0.62819	7.15482	8.38E-13	3.66E-10
JPT1	67.81492	1.368904	0.2846	4.80993	1.51E-06	8.45E-05
KATNAL1	13.02086	2.690644	0.70416	3.82107	0.000133	0.002701
KAZALD1	5.346684	1.7445	0.67171	2.59709	0.009402	0.045865

KCNA2	5.421228	5.329751	1.83211	2.90908	0.003625	0.025131
KCNA3	10.62502	1.802246	0.7053	2.5553	0.01061	0.049568
KCNA4	4.327002	4.991836	1.78657	2.79409	0.005205	0.03154
KCNAB1	2.88989	4.903742	1.7268	2.83979	0.004514	0.028874
KCNAB2	10.16469	1.853292	0.58369	3.17512	0.001498	0.014029
KCNC1	9.353783	3.945547	1.29015	3.05821	0.002227	0.018218
KCNC2	13.02775	4.458498	1.35671	3.28626	0.001015	0.01076
KCND2	9.053948	5.101358	1.35764	3.75751	0.000172	0.003268
KCND3	4.095563	3.815827	1.22231	3.12182	0.001797	0.015825
KCNG3	3.896016	4.304972	1.60639	2.67991	0.007364	0.039021
KCNH8	7.386111	3.27727	1.21918	2.68809	0.007186	0.03833
KCNIP1	3.011256	3.874407	1.38791	2.79154	0.005246	0.031704
KCNJ13	2.736534	4.239734	1.52619	2.77799	0.00547	0.032618
KCNJ15	8.250571	2.177488	0.75836	2.87132	0.004088	0.027179
KCNJ16	4.722009	4.593466	1.32738	3.46056	0.000539	0.007161
KCNJ3	5.892949	3.949513	1.34961	2.9264	0.003429	0.024145
KCNK2	3.778737	5.258861	1.37681	3.81958	0.000134	0.002708
KCNMB4	8.598802	3.682262	1.00554	3.66198	0.00025	0.004218
KCNN2	3.949836	5.364628	1.99397	2.69043	0.007136	0.038213
KCNQ1OT1	44.27983	2.518838	0.73744	3.41564	0.000636	0.008012
KCNQ5	6.508799	5.572542	1.41888	3.92741	8.59E-05	0.001989
KCNT2	12.82894	4.185057	1.08974	3.84043	0.000123	0.002562
KCNV1	7.950061	5.374125	1.44274	3.72496	0.000195	0.003557
KCTD12	61.66577	1.41379	0.41568	3.40115	0.000671	0.008231
KCTD16	13.31859	4.240084	1.18391	3.58142	0.000342	0.005242
KCTD4	12.37472	-1.11786	0.35449	-3.1534	0.001614	0.014748
KHDRBS2	6.503387	3.291798	1.19562	2.75321	0.005901	0.034087
KHDRBS3	12.33207	1.990252	0.63748	3.12208	0.001796	0.015825
KIAA0513	33.87604	1.556064	0.42391	3.67071	0.000242	0.004129
KIAA0825	13.67428	3.027206	0.98572	3.07106	0.002133	0.017686
KIAA0895	7.801879	2.433962	0.84731	2.87258	0.004071	0.027132
KIAA1671	13.03841	1.876843	0.52489	3.57569	0.000349	0.005318
KIF15	7.703717	2.020778	0.69107	2.92411	0.003454	0.024218
KIF20B	30.28099	1.375267	0.5071	2.71203	0.006687	0.036765
KIF28P	1.942057	3.809483	1.40099	2.71914	0.006545	0.036251
KIF3C	4.848567	2.313939	0.73071	3.16668	0.001542	0.014302
KLF12	15.95005	2.816208	0.74427	3.78386	0.000154	0.003032
KLF7	23.75176	1.871598	0.43289	4.32351	1.54E-05	0.000526
KLF9	13.98803	1.600321	0.62562	2.55796	0.010529	0.049368
KLHL13	4.476618	5.069195	1.17232	4.32409	1.53E-05	0.000526
KLHL14	7.062614	5.685051	1.38815	4.09541	4.21E-05	0.001143
KLHL4	5.333006	3.621101	1.16495	3.10838	0.001881	0.016264
KLHL6	10.14019	2.301132	0.7786	2.95546	0.003122	0.022777
KLKB1	2.542761	4.633714	1.42827	3.24428	0.001177	0.011927

KLRC1	4.933758	3.326149	1.03112	3.22577	0.001256	0.012507
KLRG1	5.775763	2.847661	1.05179	2.70743	0.006781	0.037122
KMT5C	5.47964	1.517053	0.58733	2.58295	0.009796	0.04712
KNSTRN	109.8521	-1.01818	0.25425	-4.0047	6.21E-05	0.001554
KRT14	17.38877	1.765077	0.55227	3.19606	0.001393	0.013407
KRT17	29.48655	2.082172	0.50202	4.14759	3.36E-05	0.000962
KRT24	6.676936	4.613329	1.02699	4.49208	7.05E-06	0.000295
KRT5	16.31414	2.798812	0.68328	4.09612	4.20E-05	0.001142
KRT6A	24.77797	2.631868	0.50138	5.24924	1.53E-07	1.33E-05
KSR2	9.874951	4.19184	1.32201	3.1708	0.00152	0.014182
KYAT1	15.68258	1.448638	0.3356	4.31652	1.59E-05	0.000541
L1CAM	4.678162	2.33446	0.65616	3.55776	0.000374	0.005605
L1TD1	12.69226	2.279153	0.60801	3.74858	0.000178	0.003359
L3MBTL4	6.869406	3.216139	1.07052	3.00427	0.002662	0.020417
LAMA1	6.697602	3.621091	1.2269	2.95141	0.003163	0.023005
LAMA4	17.99972	1.391505	0.40943	3.39864	0.000677	0.00828
LAMA5	69.9941	1.373071	0.35591	3.85791	0.000114	0.00242
LAMC2	13.00872	2.60115	0.58248	4.46563	7.98E-06	0.000324
LAPTM5	40.44045	1.423369	0.49936	2.85038	0.004367	0.028261
LARP6	2.427775	3.534393	1.34603	2.62578	0.008645	0.043324
LASP1	113.7169	1.324603	0.32261	4.10588	4.03E-05	0.001096
LBR	10.5421	1.876285	0.60599	3.09621	0.00196	0.016687
LCAL1	213.6846	-1.38231	0.30993	-4.4601	8.19E-06	0.000329
LDHA	616.2992	2.718511	0.30316	8.96721	3.04E-19	4.54E-16
LDLR	28.32347	1.300534	0.30633	4.24555	2.18E-05	0.000685
LEKR1	8.1787	2.893632	1.04131	2.77885	0.005455	0.032572
LEP	3235.57	5.230946	0.44479	11.7606	6.23E-32	1.01E-27
LETM2	26.83861	1.160659	0.34645	3.35016	0.000808	0.009271
LGALS1	41.47929	1.413545	0.4208	3.35918	0.000782	0.009112
LGALS9	11.53256	1.584958	0.58037	2.73096	0.006315	0.035579
LGR6	2.840179	4.305563	1.65052	2.60862	0.009091	0.045014
LHFPL3	2.643166	3.796204	1.3847	2.74153	0.006115	0.034837
LHFPL6	7.714018	1.38633	0.47701	2.90631	0.003657	0.025246
LHX2	2.947329	4.450988	1.38394	3.21618	0.001299	0.012783
LIMD1	330.7295	1.057819	0.31173	3.39338	0.00069	0.00836
LIMS2	41.37197	2.018749	0.47923	4.21251	2.53E-05	0.000762
LINGO2	18.59756	4.966745	1.11936	4.43714	9.12E-06	0.000359
LIPI	3.29327	5.099693	1.97671	2.57989	0.009883	0.047329
LIPK	5.192331	4.198765	1.55896	2.69331	0.007075	0.03805
LIPM	4.459815	4.536593	1.53322	2.95887	0.003088	0.022618
LMNB1	12.93452	1.273965	0.47164	2.70114	0.00691	0.037572
LMNTD1	5.804957	3.937032	1.47013	2.67802	0.007406	0.039179
LMO3	9.544376	2.993731	1.10504	2.70915	0.006745	0.03698
LPAR1	14.70504	2.274649	0.47368	4.80205	1.57E-06	8.70E-05

LPGAT1-AS1	3.440167	4.663642	1.81385	2.57113	0.010137	0.0483
LRAT	4.832919	4.040524	1.48361	2.72343	0.006461	0.036035
LRATD1	12.80857	2.065342	0.70311	2.93746	0.003309	0.02363
LRCH2	3.578622	3.215605	1.11604	2.88127	0.003961	0.026592
LRFN2	1.707029	4.144287	1.43665	2.8847	0.003918	0.0264
LRFN5	4.085413	2.853437	1.05823	2.69642	0.007009	0.037809
LRIG1	4.087168	3.53371	1.33296	2.65102	0.008025	0.041312
LRP12	8.452908	3.346939	1.029	3.25263	0.001143	0.011685
LRP1B	33.94669	4.955767	1.25388	3.95236	7.74E-05	0.001843
LRP2	32.59559	1.633868	0.57351	2.84888	0.004387	0.028324
LRRC19	2.784378	4.842515	1.7081	2.83503	0.004582	0.029085
LRRC2	6.279753	4.50721	1.43878	3.13265	0.001732	0.015413
LRRC31	5.623759	3.977649	1.50143	2.64924	0.008067	0.041429
LRRC32	60.82267	1.401879	0.26397	5.31085	1.09E-07	1.02E-05
LRRC4C	7.343024	3.805292	1.37275	2.77202	0.005571	0.032971
LRRC7	8.396162	5.978928	1.62948	3.66923	0.000243	0.004149
LRRC8A	223.7622	1.397697	0.1729	8.08388	6.27E-16	6.34E-13
LRRC8B	20.52567	1.382143	0.47358	2.91851	0.003517	0.024533
LRRC8C	8.693083	2.115656	0.80372	2.63234	0.00848	0.042748
LRRC8D	8.653946	1.82368	0.48811	3.73622	0.000187	0.003457
LRRCC1	6.37631	2.352392	0.91775	2.56323	0.01037	0.04898
LRRIQ3	4.313494	4.003473	1.37723	2.9069	0.00365	0.025242
LRRK2	25.54489	3.054451	0.81062	3.76804	0.000165	0.003173
LRRN4	1.823971	3.693157	1.30137	2.83791	0.004541	0.028983
LRRTM4	5.203147	3.440857	1.29675	2.65345	0.007967	0.041153
LSAMP	6.310019	3.684997	1.22301	3.01305	0.002586	0.020084
LSP1	25.81119	1.519583	0.46862	3.24269	0.001184	0.011964
LTA4H	32.44507	1.545663	0.39714	3.89201	9.94E-05	0.002184
LTF	29.91492	3.451123	0.657	5.25283	1.50E-07	1.32E-05
LUM	29.51144	1.477203	0.42847	3.44764	0.000565	0.007416
LUZP2	5.900942	4.944907	1.65879	2.98104	0.002873	0.021502
LY6D	73.40882	1.284765	0.38515	3.33576	0.000851	0.009539
LY6K	31.36749	1.614577	0.48175	3.35145	0.000804	0.00925
LYG2	3.873692	3.360675	1.26074	2.66564	0.007684	0.040165
LYPD1	2.549513	4.15288	1.59663	2.60102	0.009295	0.04559
LYSMD2	3.188163	3.469531	1.1398	3.04398	0.002335	0.018832
LYSMD4	4.556211	2.480925	0.89876	2.76038	0.005773	0.03366
LYVE1	10.73335	1.651115	0.62214	2.65394	0.007956	0.041119
LZTFL1	10.96618	1.678396	0.59788	2.80726	0.004997	0.030773
MACC1	44.40116	1.952276	0.31035	6.29057	3.16E-10	7.06E-08
MAF	14.59771	2.20842	0.56956	3.87741	0.000106	0.002276
MAFB	18.42332	1.353246	0.44106	3.0682	0.002154	0.017763
MAGI2	10.37186	1.900508	0.74406	2.55425	0.010642	0.049588
MAK	6.683197	2.750748	0.91183	3.01673	0.002555	0.019944

MALRD1	17.80646	4.046324	1.0198	3.96775	7.26E-05	0.001756
MAP1A	7.947031	1.679756	0.50087	3.3537	0.000797	0.009229
MAP2	13.88532	3.081026	0.94982	3.24379	0.00118	0.011938
MAP3K20	31.59697	1.431511	0.4423	3.23648	0.00121	0.012119
MAP3K7CL	61.53405	1.49128	0.47041	3.17016	0.001524	0.014193
MAPK10	17.75254	2.298358	0.68164	3.3718	0.000747	0.008832
MAPRE2	22.22843	1.660749	0.39281	4.2279	2.36E-05	0.000734
MASP1	4.481265	4.470332	1.42845	3.1295	0.001751	0.015547
MAST1	3.164944	4.490422	1.67975	2.67327	0.007512	0.039576
MATK	4.672839	2.205952	0.74294	2.9692	0.002986	0.022152
MBLAC2	7.811244	3.229618	1.00379	3.21742	0.001294	0.012746
MBNL1-AS1	11.0841	3.012785	0.86419	3.48625	0.00049	0.006717
MBOAT2	34.43186	1.179141	0.38176	3.08871	0.00201	0.016962
MBP	20.92904	1.491656	0.42025	3.54947	0.000386	0.005738
MCC	11.56922	2.622111	0.63152	4.15208	3.29E-05	0.000946
MCEMP1	2.113757	3.480182	1.16341	2.99137	0.002777	0.020981
MCM10	5.466087	2.375181	0.82382	2.88314	0.003937	0.026501
MCM6	17.30178	1.325569	0.51881	2.55501	0.010619	0.049574
MCOLN2	4.29647	2.916047	0.97728	2.98383	0.002847	0.021366
MCOLN3	4.515204	2.259495	0.81244	2.78112	0.005417	0.032449
MCTP1	13.52648	2.874263	0.68516	4.19501	2.73E-05	0.000812
MDFI	27.2225	1.33525	0.3733	3.57688	0.000348	0.005303
MDN1	67.78571	1.236396	0.37247	3.31945	0.000902	0.009926
MECOM	15.38682	2.772322	0.80547	3.44185	0.000578	0.007527
MEDAG	4.074339	2.745119	1.01365	2.70815	0.006766	0.037055
MEF2C	16.21659	1.527501	0.55695	2.74261	0.006095	0.034796
MEGF10	11.58412	2.970045	1.04708	2.8365	0.004561	0.029033
MEGF8	5.586352	2.090517	0.67452	3.09927	0.00194	0.016577
MEGF9	12.32888	1.345397	0.45941	2.92855	0.003405	0.024053
MEI1	2.209347	3.875607	1.23029	3.15015	0.001632	0.014855
MEI4	4.682145	4.592325	1.66766	2.75376	0.005892	0.034087
MGAM	4.014104	4.319137	1.43495	3.00995	0.002613	0.020173
MGAT3	30.73293	2.859549	0.48534	5.89183	3.82E-09	5.67E-07
MGAT4C	8.405271	3.93208	1.18748	3.31129	0.000929	0.010131
MGAT5B	2.150389	4.389687	1.63252	2.6889	0.007169	0.038322
MIAT	2.750887	4.816526	1.5013	3.20824	0.001336	0.01303
MICAL3	78.78215	1.480222	0.25501	5.80448	6.46E-09	8.92E-07
MID1	104.6443	1.759312	0.24376	7.21727	5.30E-13	2.45E-10
MIPOL1	13.02169	3.241398	1.04441	3.10356	0.001912	0.016434
MISP	6.646852	1.694235	0.66267	2.55668	0.010568	0.049478
MKI67	14.85549	2.18857	0.65495	3.34159	0.000833	0.00945
MKS1	6.922111	1.42836	0.51671	2.76435	0.005704	0.033434
MLIP	8.949721	2.149892	0.809	2.65746	0.007873	0.040836
MME	380.3122	2.04559	0.34567	5.91768	3.27E-09	5.12E-07

MMP14	68.31816	1.931245	0.32366	5.96694	2.42E-09	4.08E-07
MMP16	11.8657	3.811351	1.09051	3.49502	0.000474	0.006561
MMP2	12.5993	2.753596	0.59332	4.64102	3.47E-06	0.000163
MMRN1	23.63234	1.435856	0.52582	2.73068	0.00632	0.035582
MMS22L	20.27309	2.514494	0.73297	3.43056	0.000602	0.007698
MNS1	4.559233	3.664657	1.38335	2.64911	0.00807	0.041429
MOK	7.458026	1.988758	0.77528	2.5652	0.010312	0.048788
MORC1	6.661065	3.322164	1.03702	3.20356	0.001357	0.013196
MOV10L1	3.712205	5.195468	1.3229	3.92733	8.59E-05	0.001989
MOXD1	28.20232	-1.5111	0.52093	-2.9008	0.003722	0.025511
MPDZ	49.18915	1.426625	0.49907	2.85854	0.004256	0.027845
MPPED2	3.627039	4.204397	1.3749	3.05796	0.002228	0.018224
MPZL2	4.336835	2.837262	0.92979	3.0515	0.002277	0.018517
MPZL3	7.090089	2.118132	0.76937	2.75309	0.005904	0.034087
MROH2B	3.533657	3.253756	1.26304	2.57613	0.009991	0.04772
MROH9	5.366537	4.774026	1.49306	3.19748	0.001386	0.013373
MS4A2	3.46879	4.660487	1.58981	2.93147	0.003374	0.02392
MSLN	2.016104	3.279102	1.23162	2.66243	0.007758	0.040381
MSN	97.78616	1.343287	0.37627	3.56999	0.000357	0.005409
MSR1	18.06169	2.7491	0.79448	3.46025	0.00054	0.007163
MSTN	2.000375	4.308566	1.48142	2.90841	0.003633	0.025156
MTARC1	17.18042	1.4456	0.371	3.89647	9.76E-05	0.002156
MTDH	9.701064	1.984903	0.72779	2.72728	0.006386	0.03581
MTFR2	6.309639	3.061299	1.05259	2.90836	0.003633	0.025156
MTHFD2	24.28811	1.54162	0.41919	3.67762	0.000235	0.004046
MTNR1A	3.111105	4.988596	1.80512	2.76358	0.005717	0.033477
MTRNR2L2	83.36599	3.172486	0.83425	3.80282	0.000143	0.002855
MTRNR2L8	119.343	2.497377	0.73001	3.42103	0.000624	0.007898
MTSS2	275.4515	2.108143	0.31169	6.76369	1.35E-11	4.94E-09
MTUS2	4.125954	4.359033	1.35382	3.21981	0.001283	0.012703
MUC1	25.25485	2.259293	0.64032	3.52837	0.000418	0.006063
MUC15	1606.098	-1.40526	0.28666	-4.9022	9.48E-07	5.83E-05
MUC19	31.52074	2.886326	0.79665	3.6231	0.000291	0.004674
MUSK	10.63079	4.111602	1.21622	3.38063	0.000723	0.00861
MX1	27.86467	1.605138	0.35896	4.47165	7.76E-06	0.000318
MXRA7	27.88452	1.332227	0.47275	2.81805	0.004832	0.030045
MYB	5.250698	3.222857	1.13359	2.84305	0.004468	0.02869
MYBL2	6.447129	1.778928	0.69717	2.55164	0.010722	0.049823
MYBPC1	6.419602	5.073878	1.54165	3.29121	0.000998	0.010624
MYCN	10.84337	2.042021	0.58778	3.47411	0.000513	0.00694
MYCT1	11.03525	1.730201	0.55202	3.13429	0.001723	0.01537
MYEF2	20.90049	3.866589	1.03333	3.74188	0.000183	0.003422
MYH1	4.793945	4.633697	1.48769	3.11468	0.001841	0.016023
MYH15	9.873818	4.558624	1.30354	3.49711	0.00047	0.006521

MYH2	5.510528	4.439619	1.37903	3.21938	0.001285	0.012703
MYH6	13.23868	-2.48406	0.96569	-2.5723	0.010102	0.048178
MYL9	36.23163	1.519453	0.41081	3.69868	0.000217	0.003829
MYLK3	4.734393	3.725477	1.23925	3.00623	0.002645	0.020344
MYO10	26.89694	1.209788	0.30615	3.95159	7.76E-05	0.001843
MYO18B	6.311819	3.473875	1.22159	2.84374	0.004459	0.028651
MYO3A	10.06459	4.881551	1.40729	3.46876	0.000523	0.007015
MYO7B	57.30874	2.649401	0.57148	4.63606	3.55E-06	0.000165
MYOCD-AS1	1.519163	3.879877	1.50336	2.5808	0.009857	0.04726
MYOF	622.9708	1.114758	0.23212	4.80258	1.57E-06	8.70E-05
MYT1L	8.079309	2.945098	1.10981	2.65368	0.007962	0.041137
NALF1	6.016765	3.354364	1.15593	2.90188	0.003709	0.025443
NANOS1	16.47926	1.220867	0.39527	3.08873	0.00201	0.016962
NAPSB	3.668929	2.943984	1.09329	2.69278	0.007086	0.038085
NAT1	7.213358	2.841308	0.79078	3.59302	0.000327	0.005073
NAV2-AS4	133.0532	-1.43958	0.36695	-3.9231	8.74E-05	0.002007
NAV3	18.31251	3.684215	0.87249	4.22264	2.41E-05	0.000746
NBEA	27.35361	2.932062	0.94234	3.11148	0.001862	0.016135
NBEAL2	19.30181	1.211691	0.41302	2.93376	0.003349	0.023842
NBPF13P	8.571766	4.647017	0.94125	4.93708	7.93E-07	5.01E-05
NCALD	12.59789	3.914799	0.78834	4.96585	6.84E-07	4.39E-05
NCAPG	10.99579	4.104596	0.92692	4.42821	9.50E-06	0.000371
NCAPH	3.787052	1.926418	0.67239	2.86503	0.00417	0.027533
NCKAP1L	20.0707	1.777094	0.61736	2.87852	0.003996	0.026781
NCKAP5L	13.40837	1.478539	0.40799	3.624	0.00029	0.004671
NCMAP	1534.453	-1.09298	0.18414	-5.9355	2.93E-09	4.78E-07
NCOR2	146.249	1.270171	0.22925	5.54057	3.01E-08	3.45E-06
ND1	6901.722	1.330939	0.30081	4.42455	9.66E-06	0.000376
ND2	8781.759	1.300542	0.28038	4.63857	3.51E-06	0.000164
ND4	16458.17	1.006228	0.27079	3.71588	0.000202	0.003646
ND6	853.2692	1.359459	0.49436	2.74996	0.00596	0.034316
NDRG1	1217.651	1.881428	0.2774	6.78225	1.18E-11	4.45E-09
NDRG2	219.7686	1.167804	0.29631	3.94117	8.11E-05	0.001897
NDST4	2.882313	4.835928	1.66515	2.9042	0.003682	0.025346
NECTIN4	360.5037	1.128265	0.18151	6.21604	5.10E-10	9.93E-08
NEDD9	166.1783	1.930378	0.21136	9.13292	6.67E-20	1.20E-16
NEFM	6.974976	5.190875	1.61859	3.20703	0.001341	0.013066
NEIL3	9.304441	1.62426	0.5761	2.8194	0.004811	0.029994
NEK10	10.54996	2.691648	0.96088	2.80122	0.005091	0.031111
NEK11	375.9163	1.806355	0.32724	5.51998	3.39E-08	3.81E-06
NEK2	5.452291	4.835482	1.43069	3.37982	0.000725	0.008617
NEK5	8.864952	2.53479	0.8635	2.93547	0.00333	0.02376
NEK6	42.34249	2.520964	0.39383	6.40109	1.54E-10	4.22E-08
NELL1	5.519922	5.353923	1.83047	2.9249	0.003446	0.024189

NELL2	7.430006	5.788263	1.44516	4.00526	6.19E-05	0.001553
NETO1	8.054888	3.371613	1.23627	2.72725	0.006386	0.03581
NEURL1	214.9717	1.058038	0.17892	5.91331	3.35E-09	5.17E-07
NEUROD4	4.359001	4.971306	1.50806	3.29649	0.000979	0.010489
NFATC2	20.50731	1.569047	0.58817	2.66768	0.007638	0.039996
NFIB	35.83969	1.274608	0.40009	3.18584	0.001443	0.013688
NHS	16.68298	1.113795	0.39826	2.79663	0.005164	0.031408
NKAIN2	5.549784	4.36879	1.44743	3.01832	0.002542	0.019878
NKAIN3	3.752544	3.700649	1.4459	2.5594	0.010485	0.049272
NKG7	11.38453	2.547207	0.82087	3.10306	0.001915	0.016444
NLRC3	6.809314	1.846915	0.6687	2.76197	0.005745	0.033545
NLRC5	22.94375	1.442993	0.55798	2.58609	0.009707	0.046804
NLRP13	3.345774	4.572233	1.6877	2.70914	0.006746	0.03698
NLRP9	3.025419	4.948581	1.85019	2.67463	0.007481	0.039474
NME5	3.828644	4.776258	1.70045	2.80883	0.004972	0.03068
NME7	7.914974	1.989997	0.6826	2.9153	0.003553	0.024753
NME9	3.010444	2.901307	1.13508	2.55604	0.010587	0.049532
NMNAT2	179.9116	-1.11465	0.30571	-3.6461	0.000266	0.004389
NNT-AS1	5.128369	3.071837	0.89381	3.4368	0.000589	0.007613
NOCT	16.50585	1.439253	0.44785	3.21369	0.00131	0.012855
NOG	5.044034	2.804805	0.90543	3.09777	0.00195	0.016624
NOL4L	8.600805	2.014459	0.58386	3.45024	0.00056	0.00738
NOTCH3	49.15148	1.25536	0.34649	3.62308	0.000291	0.004674
NOTUM	53.90243	1.409501	0.27099	5.20123	1.98E-07	1.66E-05
NOVA1	6.307037	4.572457	1.58999	2.87577	0.00403	0.02697
NOX4	12.58509	1.866942	0.71719	2.60314	0.009237	0.045407
NPAS3	11.07633	1.124812	0.4184	2.68836	0.00718	0.038325
NPC2	111.7468	1.521994	0.20608	7.38536	1.52E-13	7.68E-11
NPHP1	8.729664	4.734881	1.47172	3.21724	0.001294	0.012746
NPNT	67.04931	3.092342	0.42106	7.34426	2.07E-13	9.84E-11
NPR3	11.7027	1.68158	0.64128	2.62222	0.008736	0.043685
NR2F1-AS1	9.329349	4.692137	1.25789	3.73015	0.000191	0.003505
NR2F2	20.6154	1.124269	0.37053	3.03423	0.002412	0.019205
NR4A3	14.9632	2.113645	0.55179	3.83052	0.000128	0.002637
NRAD1	304.7784	-2.4245	0.29328	-8.2669	1.37E-16	1.59E-13
NRAP	5.889383	4.44543	1.44294	3.08081	0.002064	0.01723
NRG1	6.38052	6.086864	2.25444	2.69994	0.006935	0.037611
NRGN	49.4471	1.255779	0.39124	3.20977	0.001328	0.012985
NRIP1	730.9535	1.347344	0.24568	5.48423	4.15E-08	4.54E-06
NRP2	10.37395	1.546465	0.48724	3.17393	0.001504	0.01407
NRXN1	7.079679	3.231984	1.1132	2.90333	0.003692	0.02539
NTF4	3.446488	2.69633	0.80346	3.35589	0.000791	0.009181
NTM	2.27087	3.480535	1.31616	2.64447	0.008182	0.041715
NTRK2	13.46523	3.437025	1.00085	3.43412	0.000594	0.007647

NTRK3	5.617479	5.337956	1.43823	3.71147	0.000206	0.003697
NUAK1	16.27388	1.696007	0.53151	3.19089	0.001418	0.01356
NUAK2	7.769579	1.644552	0.59244	2.77592	0.005505	0.032742
NUDT18	10.96881	1.689571	0.55676	3.03467	0.002408	0.019205
NUDT4B	1.77702	-3.52837	1.32298	-2.667	0.007654	0.040044
NUPR1	22.89565	1.440095	0.40385	3.56596	0.000363	0.005462
NXNL2	6.324244	1.683564	0.52997	3.17671	0.00149	0.013978
NXPE3	15.47483	2.126071	0.63756	3.33471	0.000854	0.00956
NYAP2	11.1029	4.380661	1.2895	3.39717	0.000681	0.008307
OAS1	13.77881	2.108598	0.5817	3.62491	0.000289	0.004671
OAS2	24.22548	1.652058	0.43238	3.82084	0.000133	0.002701
OAS3	21.27989	1.819481	0.47992	3.79119	0.00015	0.002967
OASL	8.041032	2.698827	0.72787	3.70785	0.000209	0.00373
OAT	22.55032	1.63667	0.44148	3.70727	0.00021	0.00373
OBSCN	48.47748	1.961785	0.39335	4.98738	6.12E-07	4.12E-05
ODAD2	7.908001	4.231876	1.20168	3.52165	0.000429	0.006152
ODC1	27.69818	1.280541	0.35047	3.65375	0.000258	0.004311
ODF2L	16.02224	1.856521	0.66092	2.80899	0.00497	0.030678
OFCC1	4.7237	2.224502	0.71399	3.11558	0.001836	0.015992
ONECUT2	7.346553	4.364169	1.33854	3.26039	0.001113	0.011471
OPN5	13.40171	3.859228	1.26349	3.05441	0.002255	0.018383
OPRK1	40.84481	-1.19107	0.28968	-4.1117	3.93E-05	0.001074
OPRM1	9.509112	4.092232	1.42933	2.86305	0.004196	0.027631
OR2C3	2.170842	3.862821	1.46335	2.63971	0.008298	0.042066
OR2T8	8.098677	1.973219	0.71024	2.77823	0.005466	0.032606
OR4F13P	2.096079	4.348494	1.51385	2.87247	0.004073	0.027132
OR4K2	2.936885	4.912465	1.71925	2.85732	0.004272	0.027879
OR52N4	4.035547	5.395218	1.74875	3.08519	0.002034	0.01704
OR7D2	4.046789	2.710278	1.05822	2.56118	0.010432	0.049141
OTOA	3.271295	5.077674	1.96852	2.57944	0.009896	0.047362
OTOL1	3.153762	4.998944	1.51039	3.3097	0.000934	0.010168
OTULINL	7.210846	2.721233	0.98353	2.7668	0.005661	0.033293
OVCH1	6.545085	3.567047	1.38155	2.58191	0.009825	0.04717
P2RX7	6.810749	2.300979	0.84495	2.72322	0.006465	0.036035
P2RY14	3.032537	3.334511	1.15474	2.88767	0.003881	0.026253
P4HA1	56.07872	1.431445	0.28067	5.10012	3.39E-07	2.56E-05
PABPC1P2	3.769737	4.752647	1.48111	3.20883	0.001333	0.013011
PABPC4L	3.643757	4.741836	1.57982	3.00151	0.002686	0.020542
PACRG	10.66234	4.137563	1.40416	2.94665	0.003212	0.023195
PADI1	5.083762	5.203282	1.0668	4.87746	1.07E-06	6.48E-05
PADI4	5.988691	1.86575	0.69226	2.69517	0.007035	0.037887
PAEP	25.27203	2.025145	0.76694	2.64056	0.008277	0.042
PAK6	115.0921	1.203486	0.23982	5.0183	5.21E-07	3.66E-05
PAK6-AS1	15.42342	1.482746	0.54277	2.73182	0.006299	0.035552

PALD1	5.098899	3.213418	0.94809	3.38937	0.000701	0.008421
PALM	4.633315	1.990542	0.69782	2.85253	0.004337	0.028116
PAMR1	5.376805	2.679766	1.031	2.59918	0.009345	0.045753
PAPPA2	27153.73	2.138789	0.29074	7.35629	1.89E-13	9.26E-11
PAPSS2	51.60635	1.088532	0.42595	2.55552	0.010603	0.049568
PARD3B	18.0071	1.530349	0.55579	2.75347	0.005897	0.034087
PARP9	53.98742	1.032396	0.40385	2.55639	0.010577	0.049505
PARPBP	11.8936	1.571376	0.58107	2.70427	0.006846	0.037351
PART1	5.197207	5.236807	1.50537	3.47875	0.000504	0.006873
PARVB	9.971729	1.909379	0.58747	3.25015	0.001153	0.011743
PARVG	8.058298	2.045043	0.7319	2.79414	0.005204	0.03154
PASK	7.017456	1.81505	0.51184	3.54611	0.000391	0.005772
PATJ	30.99512	1.825253	0.47721	3.82486	0.000131	0.002664
PAX6	6.749808	3.496018	1.1839	2.95296	0.003147	0.022911
PCA3	3.932837	5.335376	1.75831	3.03437	0.00241	0.019205
PCAT4	3.35005	5.125754	1.97442	2.59608	0.009429	0.045954
PCDH17	5.966787	3.37838	1.09458	3.08646	0.002026	0.017021
PCDH20	4.283567	3.988637	1.4665	2.71983	0.006532	0.036225
PCDH7	6.594574	4.361695	1.45471	2.99832	0.002715	0.020683
PCDH9	17.04154	5.062861	1.64211	3.08315	0.002048	0.01714
PCDHB4	4.01396	4.854373	1.67096	2.90514	0.003671	0.025293
PCED1B-AS1	4.771878	3.254989	1.08951	2.98758	0.002812	0.021204
PCGF2	7.856184	5.416426	1.5577	3.4772	0.000507	0.006905
PCLAF	4.143613	3.948059	1.36731	2.88747	0.003884	0.026254
PCNX2	8.079166	2.0194	0.68595	2.94396	0.00324	0.023335
PCSK1	5.487682	4.360526	1.4208	3.06907	0.002147	0.017739
PCSK5	10.33795	2.239333	0.7464	3.00019	0.002698	0.020595
PCYT1B	4.701302	3.593261	1.09376	3.28525	0.001019	0.010774
PDE11A	8.54541	4.525148	1.57982	2.86434	0.004179	0.027575
PDE1A	11.07611	2.990238	1.02965	2.90414	0.003683	0.025346
PDE1C	12.67076	2.711502	0.91392	2.96688	0.003008	0.022259
PDE2A	8.597747	1.96114	0.66144	2.96496	0.003027	0.022347
PDE3A	18.80652	1.770042	0.64437	2.74693	0.006016	0.034488
PDE4B	13.15515	1.362779	0.51368	2.65296	0.007979	0.041181
PDE7B	35.14439	-1.18531	0.39265	-3.0188	0.002538	0.019864
PDGFC	8.78955	2.124836	0.7627	2.78595	0.005337	0.032089
PDGFD	25.10977	-1.52095	0.57844	-2.6294	0.008554	0.043013
PDGFRA	19.42569	1.328405	0.45477	2.92103	0.003489	0.024396
PDGFRB	15.79253	1.100609	0.39302	2.80041	0.005104	0.03116
PDLIM1	48.44265	1.023351	0.32798	3.12018	0.001807	0.015829
PDZD7	7.828836	2.875385	0.76598	3.75385	0.000174	0.003304
PDZK1IP1	11.99686	2.916537	0.881	3.31049	0.000931	0.010153
PDZRN3	5.376482	4.768675	1.303	3.65976	0.000252	0.004247
PECR	3.093264	3.973284	1.53941	2.58104	0.00985	0.047249

PEG3	984.3419	1.009573	0.28462	3.5471	0.000389	0.005761
PENK	5.118069	2.653122	0.84034	3.1572	0.001593	0.014624
PEX5L	9.804773	5.261446	1.88419	2.79242	0.005232	0.031654
PFKFB3	14.09291	2.172887	0.63745	3.40872	0.000653	0.008127
PFKP	17.6939	2.820288	0.47793	5.90106	3.61E-09	5.51E-07
PGLYRP4	5.935947	2.795033	1.02092	2.73776	0.006186	0.035115
PGM5	11.24293	1.891076	0.59108	3.19938	0.001377	0.013317
PGM5P2	5.282155	3.836741	1.38738	2.76546	0.005684	0.033381
PGR	14.55836	2.782642	0.99549	2.79526	0.005186	0.031471
PGR-AS1	2.1865	3.887092	1.50317	2.58593	0.009712	0.046809
PHACTR1	8.175222	2.975981	0.92598	3.21388	0.00131	0.012855
PHLDA1	11.10914	1.762908	0.62345	2.82767	0.004689	0.02953
PHYHIP	82.71852	1.702091	0.3585	4.74776	2.06E-06	0.000109
PIEZO1	16.90685	1.099612	0.40425	2.7201	0.006526	0.036216
PIEZO2	8.106996	3.064427	0.92643	3.30776	0.00094	0.010231
PIK3AP1	994.4816	1.016166	0.17872	5.68592	1.30E-08	1.70E-06
PIK3CG	10.69615	1.526554	0.55207	2.76513	0.00569	0.033387
PIK3R1	554.1167	1.094988	0.25915	4.22537	2.39E-05	0.00074
PIK3R6	3.764144	3.249665	1.20746	2.69132	0.007117	0.038158
PIM2	7.616473	1.739905	0.66327	2.62324	0.00871	0.043582
PITPNC1	11.41648	1.486629	0.53411	2.78337	0.00538	0.032297
PITPNM1	36.46578	1.062814	0.32643	3.25589	0.00113	0.011603
PITPNM3	3.848299	2.68072	0.89546	2.99366	0.002757	0.020889
PIWIL3	4.338507	2.628934	0.99176	2.65078	0.008031	0.041321
PKD1L2	8.427384	2.512887	0.61604	4.0791	4.52E-05	0.001208
PKDCC	2.542693	3.590051	1.19993	2.99189	0.002773	0.020955
PKHD1L1	35.17539	2.770429	0.6869	4.03325	5.50E-05	0.001409
PKM	915.3798	1.118159	0.1708	6.54675	5.88E-11	1.90E-08
PKP4	15.0774	1.91373	0.70844	2.70134	0.006906	0.037572
PLA2G15	9.996864	1.555318	0.41681	3.73147	0.00019	0.003496
PLA2G2F	36.69161	1.205623	0.39049	3.08743	0.002019	0.017009
PLA2G4A	8.603194	5.485807	1.29112	4.24888	2.15E-05	0.000678
PLAAT4	53.0391	1.18243	0.40068	2.95108	0.003167	0.02302
PLAC8	33.31315	1.7857	0.30418	5.87048	4.35E-09	6.39E-07
PLAGL1	15.59691	1.242424	0.47491	2.6161	0.008894	0.044298
PLCB4	11.14938	2.921834	0.79405	3.67966	0.000234	0.00403
PLCG2	24.80373	1.313361	0.45548	2.88349	0.003933	0.026482
PLCH1	9.124672	3.037479	0.96813	3.13748	0.001704	0.015272
PLCL1	12.35351	3.926643	1.08615	3.61519	0.0003	0.004766
PLCXD3	6.808849	4.193477	1.13064	3.70893	0.000208	0.00372
PLCZ1	9.735268	4.283165	1.26097	3.39671	0.000682	0.008307
PLEC	712.9079	1.097405	0.2055	5.34021	9.28E-08	9.04E-06
PLEK	39.67222	1.465165	0.49786	2.9429	0.003252	0.023363
PLEKHA2	157.2923	2.943838	0.32835	8.96556	3.09E-19	4.54E-16

PLEKHH2	7.171621	2.921407	0.84242	3.46789	0.000525	0.007032
PLEKHO1	17.14807	1.320782	0.45256	2.91847	0.003517	0.024533
PLEKHS1	3.172761	4.516461	1.60002	2.82275	0.004761	0.029801
PLG	3.490838	5.158994	1.74744	2.95232	0.003154	0.022948
PLOD2	68.91044	2.166466	0.33158	6.53374	6.41E-11	1.99E-08
PLPP2	25.52081	-1.72901	0.63286	-2.7321	0.006294	0.03554
PLPPR4	6.080394	3.690311	1.41697	2.60438	0.009204	0.045312
PLPPR5	5.049771	4.677298	1.43951	3.24923	0.001157	0.011766
PLS3	15.12993	1.567795	0.47523	3.29905	0.00097	0.010414
PLSCR2	7.364902	3.235218	1.01118	3.19944	0.001377	0.013317
PLTP	10.54458	1.701594	0.56038	3.0365	0.002393	0.019137
PLXNA4	8.280184	2.876834	1.06533	2.70041	0.006925	0.037605
PMAIP1	38.27512	-1.69834	0.31416	-5.406	6.45E-08	6.59E-06
PMEL	12.1019	1.499691	0.42905	3.49541	0.000473	0.006557
PMEPA1	5.358096	2.824267	0.73194	3.85862	0.000114	0.002416
PMP2	4.597097	5.066273	1.68167	3.01265	0.00259	0.020084
PNCK	8.602167	2.622888	0.52655	4.9813	6.32E-07	4.18E-05
PNMA5	3.882685	3.856171	1.45644	2.64766	0.008105	0.041517
POLQ	13.4167	2.731999	0.80118	3.40997	0.00065	0.008126
POLR3GL	4.076088	2.760596	0.89432	3.0868	0.002023	0.017019
POSTN	646.5099	-1.49765	0.31257	-4.7913	1.66E-06	9.02E-05
POT1-AS1	6.195281	3.130823	1.1156	2.80639	0.00501	0.030819
POTEC	5.693893	4.402229	1.54685	2.84593	0.004428	0.028534
POTEH	3.093106	4.965885	1.59415	3.11506	0.001839	0.016011
PPARGC1A	9.132056	3.334384	1.21292	2.74907	0.005977	0.034385
PPFIA3	7.351738	2.433837	0.67213	3.62106	0.000293	0.004692
PPFIBP1	94.67883	1.395497	0.30401	4.59025	4.43E-06	0.000199
PPFIBP2	9.233009	2.844032	0.77263	3.681	0.000232	0.004013
PPIA	161.7193	1.269024	0.2616	4.85102	1.23E-06	7.22E-05
PPIAP46	93.25738	4.715252	0.7556	6.24037	4.37E-10	8.82E-08
PPL	218.0468	1.050356	0.26569	3.95327	7.71E-05	0.001843
PPM1E	4.039344	4.876977	1.57877	3.08911	0.002008	0.016962
PPM1F	47.11769	1.070142	0.30964	3.45612	0.000548	0.007244
PPM1K	12.78084	1.734143	0.51552	3.36388	0.000769	0.008997
PPP1R12C	332.0576	1.125874	0.17449	6.45226	1.10E-10	3.18E-08
PPP1R1C	19.83812	2.78247	0.53513	5.19957	2.00E-07	1.66E-05
PPP1R3A	3.561912	5.204245	2.03009	2.56355	0.010361	0.048949
PPP2R2B	15.50607	1.175147	0.46081	2.55015	0.010768	0.049959
PREX1	55.0214	1.209596	0.32946	3.67141	0.000241	0.004122
PREX2	14.4593	1.915221	0.73505	2.60556	0.009173	0.045214
PRG2	399.4885	1.461073	0.24467	5.97156	2.35E-09	4.04E-07
PRICKLE2	8.716231	2.287487	0.73668	3.10511	0.001902	0.016367
PRKAA2	29.92854	1.28386	0.42891	2.99329	0.00276	0.020889
PRKAB2	70.45851	1.662048	0.33218	5.00346	5.63E-07	3.84E-05

PRKCB	21.84467	1.389875	0.49171	2.82662	0.004704	0.029562
PRKD1	8.770402	3.074584	1.11017	2.76947	0.005615	0.033105
PRKD3	276.3484	2.467623	0.24659	10.0071	1.42E-23	3.82E-20
PRKX	18.81349	2.046351	0.57658	3.54914	0.000386	0.005738
PRKXP1	4.112019	2.604876	0.87795	2.96699	0.003007	0.022259
PRNCR1	9.190276	2.970347	1.02757	2.89066	0.003844	0.026158
PROS1	13.67791	2.158294	0.68727	3.14037	0.001687	0.015189
PROSER2	5.062348	3.27213	0.74652	4.38319	1.17E-05	0.000433
PROX1	15.89483	2.745136	0.67964	4.03911	5.37E-05	0.001377
PRPS2	3.955121	2.356337	0.88929	2.64968	0.008057	0.041403
PRR16	2.362564	4.028065	1.49429	2.69563	0.007026	0.037873
PRRX1	5.802571	3.954871	0.90487	4.37066	1.24E-05	0.000454
PRSS12	17.7097	2.732202	0.73805	3.70191	0.000214	0.003802
PRSS22	4.3997	4.544924	1.35228	3.36092	0.000777	0.009074
PRXL2A	206.3681	-1.62274	0.34849	-4.6565	3.22E-06	0.000153
PTCH1	11.06795	2.023784	0.63147	3.20486	0.001351	0.013145
PTGDS	4.721657	1.866221	0.72828	2.56249	0.010392	0.048998
PTGER3	11.85413	3.183397	0.91767	3.46901	0.000522	0.007015
PTGER4	4.687099	5.055131	1.32056	3.82803	0.000129	0.002647
PTGIS	6.213162	2.98132	0.85692	3.47911	0.000503	0.006869
PTGS1	42.4539	1.531488	0.46473	3.29543	0.000983	0.010508
PTK2B	24.80955	1.500699	0.43278	3.46754	0.000525	0.007035
PTPDC1	5.831923	2.515398	0.80213	3.13588	0.001713	0.015304
PTPN13	22.42715	2.679979	0.56686	4.72772	2.27E-06	0.000117
PTPN22	9.352291	2.49848	0.83589	2.98902	0.002799	0.021114
PTPRC	16.62286	3.266308	0.75851	4.30621	1.66E-05	0.000561
PTPRD	16.3082	2.986114	0.98372	3.03552	0.002401	0.01918
PTPRE	13.79282	1.561	0.45397	3.43856	0.000585	0.007582
PTPRO	8.393684	2.787679	1.03123	2.70327	0.006866	0.037401
PTPRQ	68.73009	-1.10794	0.36279	-3.054	0.002258	0.018383
PTPRR	23.17541	2.178008	0.59896	3.63635	0.000277	0.00452
PTPRT	6.33228	3.4819	1.27236	2.73658	0.006208	0.035192
PTPRU	53.88856	1.49341	0.26829	5.56639	2.60E-08	3.14E-06
PTPRZ1	11.82196	3.806351	1.25343	3.03676	0.002391	0.019137
PTTG1	5.794294	3.348629	0.92392	3.62439	0.00029	0.004671
PWAR5	6.94688	4.321599	1.55478	2.77955	0.005443	0.032546
PWRN2	4.205976	4.939381	1.93246	2.556	0.010588	0.049532
PXDNL	8.090205	2.173553	0.79674	2.72805	0.006371	0.035764
PZP	8.541436	4.302352	1.48833	2.89072	0.003844	0.026158
QPCT	14.62164	2.59719	0.53929	4.8159	1.47E-06	8.28E-05
QSOX1	476.4613	1.362913	0.28311	4.81399	1.48E-06	8.33E-05
RAB17	29.64211	1.227698	0.47258	2.59786	0.009381	0.045832
RAB39A	16.74104	-1.29053	0.43793	-2.9469	0.00321	0.023195
RAB9B	2.156269	3.002278	1.1039	2.7197	0.006534	0.036226

RAC2	18.815	1.388071	0.50907	2.72667	0.006398	0.035833
RACGAP1	25.78922	1.970888	0.49466	3.98432	6.77E-05	0.00167
RAD21L1	5.136306	4.249229	1.53754	2.76366	0.005716	0.033477
RAD51AP2	2.870444	4.347212	1.66027	2.61838	0.008835	0.044071
RAD54L	2.608709	4.20157	1.59226	2.63874	0.008321	0.042134
RAI2	3.813563	2.590268	0.93463	2.77143	0.005581	0.032991
RALYL	5.485814	5.829503	1.56232	3.73131	0.00019	0.003496
RAPGEF4	7.569954	3.369089	1.03098	3.26784	0.001084	0.011246
RAPH1	198.8385	-1.05166	0.18963	-5.546	2.92E-08	3.42E-06
RARRES1	13.2933	5.35016	1.01246	5.28431	1.26E-07	1.15E-05
RASA3	17.92886	1.704066	0.5233	3.25636	0.001128	0.011597
RASAL1	4.302299	3.231354	1.00588	3.21247	0.001316	0.012894
RASAL3	4.352515	1.839812	0.68607	2.68165	0.007326	0.03887
RASEF	153.4854	2.441527	0.31039	7.86607	3.66E-15	3.29E-12
RASGRF2	39.22902	1.68015	0.40641	4.13417	3.56E-05	0.001005
RASL11B	67.02415	-1.13493	0.30596	-3.7094	0.000208	0.00372
RASSF3	89.68101	1.244456	0.26432	4.70809	2.50E-06	0.000126
RASSF5	17.41398	1.672533	0.54961	3.04315	0.002341	0.018858
RASSF9	8.757628	2.643131	0.89381	2.95715	0.003105	0.022703
RBM44	4.930227	4.17999	1.38942	3.00845	0.002626	0.020235
RBM46	3.333396	3.557102	1.30835	2.71876	0.006553	0.036268
RBP1	5.260155	2.240289	0.72874	3.07421	0.002111	0.01758
RCAN1	153.4758	-1.87773	0.30708	-6.1148	9.67E-10	1.78E-07
RCOR2	3.393006	2.126375	0.83261	2.55387	0.010653	0.049592
RCSD1	12.64152	1.677493	0.54861	3.05771	0.00223	0.01823
RDH16	3.481269	2.778698	0.9661	2.87621	0.004025	0.026944
RELN	17.52664	3.114599	1.00149	3.10997	0.001871	0.016193
RELT	14.41356	1.392324	0.50338	2.76593	0.005676	0.033351
REXO5	3.955229	2.409286	0.87648	2.74882	0.005981	0.034396
RFLNB	13.03551	1.659564	0.42535	3.90168	9.55E-05	0.002145
RFX6	4.730309	3.65556	1.39982	2.61146	0.009016	0.044738
RGN	2.872184	3.741123	1.32231	2.82924	0.004666	0.029501
RGPD4	7.934326	1.525781	0.58528	2.60692	0.009136	0.045142
RGS16	13.23626	2.227405	0.71381	3.12046	0.001806	0.015825
RGS22	9.717625	3.430985	0.90932	3.77313	0.000161	0.003129
RGS3	20.46555	1.449269	0.42139	3.43928	0.000583	0.007576
RGS5-AS1	5.736195	5.396123	1.63446	3.30148	0.000962	0.010352
RGS8	2.014196	4.417522	1.69949	2.59932	0.009341	0.045748
RGS9	2.742918	3.125954	1.20122	2.60233	0.009259	0.045472
RGSL1	4.409251	5.49341	1.60131	3.43057	0.000602	0.007698
RHOF	51.96317	1.888065	0.37081	5.09167	3.55E-07	2.66E-05
RHOH	6.613849	2.686904	1.00547	2.67227	0.007534	0.039661
RHPN2	15.96993	1.120708	0.3909	2.86699	0.004144	0.027401
RIMS1	14.71241	3.735076	1.07638	3.47005	0.00052	0.007011

RIMS2	18.72993	4.608153	1.14894	4.01079	6.05E-05	0.001522
RIMS3	2.766517	2.822743	0.98184	2.87495	0.004041	0.027006
RIPOR1	13.03428	1.666217	0.39492	4.21917	2.45E-05	0.000748
RIT2	5.35142	3.418491	1.33384	2.56289	0.01038	0.048983
RNASE11	18.86758	-2.61177	0.47935	-5.4485	5.08E-08	5.40E-06
RNASEH2C	75.36976	1.108121	0.26282	4.2163	2.48E-05	0.000753
RNF112	2.313441	3.931952	1.1531	3.40989	0.00065	0.008126
RNF125	7.373518	2.618144	0.82361	3.17885	0.001479	0.013914
RNF144B	25.56625	1.305412	0.44044	2.96388	0.003038	0.022385
RNF150	9.224953	3.631228	1.14278	3.17753	0.001485	0.013962
RNF152	75.6058	-1.66535	0.34458	-4.833	1.35E-06	7.82E-05
RNF165	4.215042	4.425453	1.70933	2.589	0.009626	0.046546
RNF17	11.86902	4.015558	1.14403	3.51001	0.000448	0.00631
RNF175	4.558903	4.545421	1.7751	2.56065	0.010448	0.049187
RNF180	8.926644	4.554576	1.23222	3.69623	0.000219	0.003845
RNF212	2.103865	4.373516	1.58938	2.75172	0.005928	0.034193
RNF212B	3.245746	4.021686	1.56751	2.56566	0.010298	0.048767
RNF222	3.431679	3.649456	1.12132	3.25459	0.001136	0.011634
RNF32-DT	2.140703	4.430234	1.53118	2.89335	0.003812	0.026
RNFT1	8.661801	1.685844	0.61316	2.74942	0.00597	0.03436
RNR1	422187.9	1.111406	0.41576	2.67322	0.007513	0.039576
ROBO1	13.53722	3.046221	1.00217	3.03963	0.002369	0.019008
ROBO2	18.00084	4.461131	1.07854	4.13627	3.53E-05	0.000999
RORB	8.709984	3.033972	0.70582	4.29853	1.72E-05	0.000577
ROS1	14.879	4.08702	1.07863	3.78909	0.000151	0.002985
RPE65	2.700584	4.777205	1.75563	2.72108	0.006507	0.036151
RPGRIP1L	12.32938	2.693275	0.79897	3.37091	0.000749	0.008835
RPS16P5	2.108915	3.895623	1.43015	2.72393	0.006451	0.036
RPS6KA2	16.34885	1.586027	0.50044	3.16927	0.001528	0.014213
RPS6KA4	40.06491	1.233367	0.30668	4.02168	5.78E-05	0.00146
RRAS2	854.116	-1.09274	0.16607	-6.58	4.71E-11	1.56E-08
RRM2	9.053393	2.914337	0.74901	3.89089	9.99E-05	0.002188
RRN3P1	2.721311	4.745868	1.25861	3.77073	0.000163	0.003152
RSAD2	16.32436	1.400309	0.49012	2.85707	0.004276	0.027879
RTKN2	13.55464	3.426555	1.03352	3.31542	0.000915	0.010016
RTL6	12.01932	1.489894	0.5342	2.78904	0.005286	0.03189
RUBCNL	4.257209	3.422134	1.20569	2.83831	0.004535	0.028969
RUNX1	16.56067	2.116899	0.59501	3.55775	0.000374	0.005605
RUNX1T1	11.00092	2.516559	0.95072	2.647	0.008121	0.041551
RUNX3	7.754004	1.908243	0.74712	2.55414	0.010645	0.049588
RUSC2	60.9638	1.558012	0.24871	6.26446	3.74E-10	7.96E-08
RXFP1	10.63042	2.236407	0.80719	2.77061	0.005595	0.033037
RYR1	7.472713	2.772008	1.01153	2.74041	0.006136	0.034932
RYR2	42.65436	1.972152	0.66715	2.95608	0.003116	0.022752

RYR3	14.26909	3.080479	0.97971	3.14426	0.001665	0.015044
S100A4	53.77877	1.782519	0.43237	4.12267	3.75E-05	0.00104
S1PR2	11.97063	2.211558	0.54278	4.07448	4.61E-05	0.001224
SACS	11.04925	2.785851	0.77953	3.57375	0.000352	0.005347
SALL4	5.068402	4.731126	1.63498	2.89369	0.003807	0.025984
SAMD13	3.942599	3.814269	1.27913	2.98193	0.002864	0.021469
SAMD3	6.661552	2.284739	0.88374	2.58531	0.009729	0.046854
SAMD4A	114.1948	1.232929	0.33868	3.64041	0.000272	0.004468
SAMD5	8.353738	2.576391	1.00828	2.55524	0.010611	0.049568
SAMD9	18.57643	1.289221	0.48982	2.63202	0.008488	0.042776
SAMSN1	6.59837	2.947731	0.82404	3.57719	0.000347	0.005302
SAP30L-AS1	3.116868	4.96806	1.55389	3.19717	0.001388	0.013379
SASH1	753.7976	2.789537	0.27865	10.0109	1.37E-23	3.82E-20
SATB1	18.71081	1.745664	0.62219	2.80569	0.005021	0.030864
SATB2	37.58051	1.260396	0.30281	4.16236	3.15E-05	0.000913
SAXO2	3.586108	3.285338	1.19776	2.7429	0.00609	0.034778
SCD	29.82578	1.885305	0.36108	5.22128	1.78E-07	1.53E-05
SCEL	6.457302	2.321172	0.86947	2.66964	0.007593	0.039858
SCFD2	13.35113	1.559621	0.45419	3.43388	0.000595	0.007647
SCML4	5.715912	4.34264	1.12307	3.86676	0.00011	0.002362
SCN10A	4.121202	5.430053	1.97878	2.74414	0.006067	0.034696
SCN1A	13.17872	5.227987	1.3905	3.75979	0.00017	0.003242
SCN1A-AS1	3.250855	5.053405	1.74606	2.89417	0.003802	0.025955
SCN3A	17.23047	3.499903	1.16201	3.01193	0.002596	0.0201
SCN7A	13.29681	3.317339	1.19601	2.77366	0.005543	0.032849
SCN8A	9.094355	2.552107	0.99872	2.55538	0.010607	0.049568
SCRN1	14.99512	1.14024	0.38978	2.92536	0.003441	0.024189
SDC2	5.382685	2.150162	0.63085	3.40837	0.000654	0.008127
SDC3	90.18903	2.070289	0.3269	6.33317	2.40E-10	5.80E-08
SDC4	184.216	1.046081	0.16644	6.28518	3.27E-10	7.15E-08
SEC16B	3.819502	2.71712	0.95771	2.83711	0.004552	0.028998
SEL1L2	4.117656	4.362841	1.58237	2.75716	0.005831	0.033902
SELENOP	25.93242	1.14047	0.26799	4.25565	2.08E-05	0.000661
SELL	10.59052	2.487238	0.70561	3.52494	0.000424	0.006103
SELP	8.471275	1.668656	0.59676	2.79617	0.005171	0.03143
SEMA3A	15.46497	2.237094	0.73858	3.02891	0.002454	0.019489
SEMA3D	15.19567	4.589614	1.55022	2.96063	0.00307	0.022541
SEMA3E	7.741737	3.377514	1.18273	2.85569	0.004294	0.027939
SEMA4C	51.2253	2.10711	0.31475	6.69451	2.16E-11	7.77E-09
SEMA4D	29.21403	1.191424	0.43022	2.76934	0.005617	0.033106
SEMA5A	12.69594	1.822257	0.65963	2.76253	0.005735	0.033511
SEMA5B	2.047739	3.748992	1.27134	2.94885	0.00319	0.023129
SEPTIN14	3.506872	4.09444	1.35304	3.0261	0.002477	0.019537
SEPTIN4	8.629982	1.35545	0.47704	2.84138	0.004492	0.028806

SEPTIN9	144.7013	1.070572	0.21667	4.94105	7.77E-07	4.95E-05
SERPINA3	16.56023	5.531144	1.10356	5.01211	5.38E-07	3.75E-05
SERPINB11	2.926852	4.856647	1.63263	2.97475	0.002932	0.021832
SERPINE3	5.749539	4.909782	1.68932	2.90637	0.003656	0.025246
SERPING1	25.68043	1.727133	0.42302	4.08283	4.45E-05	0.001195
SERPINI1	30.93517	-1.19859	0.34793	-3.4449	0.000571	0.00746
SETDB2	24.62932	1.093886	0.39177	2.79219	0.005235	0.031664
SFN	8.386771	2.546544	0.90039	2.82827	0.00468	0.02953
SFR1	29.95722	1.906939	0.48692	3.91632	8.99E-05	0.002053
SFRP1	17.62712	2.134515	0.72983	2.92466	0.003448	0.024196
SFXN3	363.9894	1.556253	0.287	5.42251	5.88E-08	6.13E-06
SGCB	39.48034	-1.34595	0.31685	-4.2479	2.16E-05	0.00068
SGCD	8.123076	3.640784	1.21172	3.00464	0.002659	0.020412
SGCZ	3.031779	4.384678	1.56115	2.80862	0.004975	0.03068
SGIP1	6.294465	4.55405	1.31359	3.46687	0.000527	0.007041
SGO1	11.06079	3.576871	1.11831	3.19847	0.001382	0.013343
SGPP2	7.539918	2.355138	0.82639	2.8499	0.004373	0.028276
SGSM2	17.83258	1.22602	0.38545	3.18074	0.001469	0.013832
SH2D1B	6.868398	4.349468	1.31307	3.31245	0.000925	0.010096
SH3BGRL	56.56306	1.168036	0.39732	2.93978	0.003284	0.023511
SH3BP5	1122.892	2.433359	0.32356	7.52065	5.45E-14	3.04E-11
SH3GL2	2.725645	4.793491	1.74959	2.73977	0.006148	0.034974
SH3PXD2A	8658.092	3.074907	0.488	6.30104	2.96E-10	6.83E-08
SH3PXD2B	30.83343	1.74137	0.33489	5.19978	2.00E-07	1.66E-05
SH3RF2	3.631094	4.245361	1.36198	3.11704	0.001827	0.015941
SHC3	24.39563	3.530301	0.71324	4.94966	7.43E-07	4.75E-05
SHE	7.575336	1.784825	0.62406	2.86002	0.004236	0.027783
SHF	7.982197	1.32726	0.46719	2.84091	0.004498	0.028828
SHISA9	7.313635	4.040907	1.37131	2.94675	0.003211	0.023195
SHISAL1	4.392614	3.900788	1.36558	2.8565	0.004283	0.027901
SHOC1	8.30702	4.592995	1.36425	3.36668	0.000761	0.00892
SI	21.05695	5.316048	1.39692	3.80556	0.000141	0.002834
SIGLEC6	574.1169	2.734428	0.61418	4.45219	8.50E-06	0.000338
SIGLECL1	2.767204	4.809883	1.79637	2.67755	0.007416	0.039221
SILC1	4.457782	4.510812	1.67311	2.69606	0.007017	0.037838
SIPA1L2	16.63626	1.510425	0.5684	2.65731	0.007877	0.040841
SIPA1L3	13.77865	1.343712	0.41637	3.22724	0.00125	0.012459
SKIDA1	14.88102	1.407655	0.50652	2.77909	0.005451	0.032572
SKP2	60.48729	1.217585	0.30014	4.05669	4.98E-05	0.001294
SLA	21.08558	2.099313	0.76571	2.74165	0.006113	0.034837
SLC11A1	37.20797	1.279563	0.25553	5.00742	5.52E-07	3.81E-05
SLC12A1	4.81595	4.149773	1.50672	2.75417	0.005884	0.034072
SLC12A2	20.79709	1.632485	0.63767	2.56009	0.010465	0.049223
SLC12A9	12.50741	1.07266	0.33703	3.18271	0.001459	0.013786

SLC13A1	8.188783	5.917133	1.42011	4.16668	3.09E-05	0.0009
SLC16A3	191.6307	1.191652	0.22468	5.30367	1.13E-07	1.05E-05
SLC16A7	18.6338	3.56121	1.06155	3.35473	0.000794	0.009213
SLC17A1	2.227362	4.499068	1.73521	2.59282	0.009519	0.046184
SLC17A4	5.37707	4.228736	1.31619	3.21286	0.001314	0.012884
SLC19A3	212.6589	-1.07139	0.28641	-3.7407	0.000183	0.003426
SLC1A1	4.712715	4.097283	1.18176	3.46712	0.000526	0.007041
SLC1A2	15.01203	1.52613	0.59405	2.56901	0.010199	0.048481
SLC1A5	20.14872	1.386802	0.42181	3.28771	0.00101	0.010722
SLC1A6	11.25577	5.427686	1.07102	5.06777	4.03E-07	2.97E-05
SLC22A23	36.26067	1.490938	0.30836	4.83499	1.33E-06	7.80E-05
SLC22A25	4.290606	4.467616	1.74816	2.55561	0.0106	0.049568
SLC22A3	4.63401	4.558523	1.41962	3.21109	0.001322	0.012933
SLC23A3	2.288217	3.368013	1.26366	2.66528	0.007692	0.040195
SLC24A2	6.933541	3.193724	1.16612	2.73877	0.006167	0.035044
SLC24A5	3.567791	5.167542	1.57148	3.28833	0.001008	0.010713
SLC25A21	3.025481	3.852101	1.42415	2.70484	0.006834	0.037312
SLC25A30	77.66702	1.033778	0.29497	3.50465	0.000457	0.006411
SLC26A5	6.309813	3.485604	1.25642	2.77424	0.005533	0.032827
SLC27A1	2.845682	3.797718	1.19319	3.18284	0.001458	0.013786
SLC28A1	25.40651	1.782014	0.38826	4.58979	4.44E-06	0.000199
SLC28A2	3.028645	4.946512	1.82128	2.71595	0.006608	0.036439
SLC2A10	4.363737	2.620792	0.91239	2.87245	0.004073	0.027132
SLC2A3	16.77726	1.664274	0.38427	4.33099	1.48E-05	0.000517
SLC2A5	9.127908	1.330383	0.50721	2.62294	0.008717	0.043606
SLC2A9	17.17457	1.350465	0.41243	3.27442	0.001059	0.011058
SLC35F1	3.828541	4.763149	1.70887	2.7873	0.005315	0.032002
SLC35G1	6.130501	4.414062	1.29607	3.40572	0.00066	0.008171
SLC38A4	7.212746	5.250696	1.52935	3.43329	0.000596	0.007651
SLC40A1	1492.223	-1.02517	0.20876	-4.9108	9.07E-07	5.64E-05
SLC44A3	23.74337	1.66606	0.43936	3.79199	0.000149	0.002964
SLC44A3-AS1	13.55434	1.826084	0.49655	3.67753	0.000236	0.004046
SLC45A4	385.884	-1.23785	0.23225	-5.3297	9.84E-08	9.35E-06
SLC4A10	10.48102	5.799243	1.37632	4.21358	2.51E-05	0.00076
SLC4A8	14.79273	3.408391	1.09848	3.10282	0.001917	0.016449
SLC52A3	15.72763	1.02149	0.37145	2.75001	0.005959	0.034316
SLC5A11	6.075078	3.305428	1.16576	2.83543	0.004576	0.029081
SLC6A14	6.267048	5.031403	1.6039	3.13699	0.001707	0.01528
SLC6A8	173.0911	1.857432	0.29517	6.2927	3.12E-10	7.06E-08
SLC6A9	12.42984	1.2615	0.38317	3.29226	0.000994	0.010599
SLC7A13	2.898712	3.786078	1.43906	2.63094	0.008515	0.042858
SLC7A14	3.819628	4.247132	1.49426	2.84229	0.004479	0.028735
SLC9A3-AS1	6.604369	1.8665	0.72629	2.56992	0.010172	0.048425
SLC9A7	9.436417	2.13604	0.81984	2.60543	0.009176	0.045214

SLC9A9	11.22527	2.523492	0.7416	3.40279	0.000667	0.008219
SLC9B2	5.817759	3.124545	1.09094	2.86409	0.004182	0.027585
SLC9C2	5.913071	2.978537	1.1442	2.60315	0.009237	0.045407
SLCO1A2	14.72137	5.341013	1.25523	4.25501	2.09E-05	0.000661
SLCO1B1	3.131536	5.031986	1.90395	2.64292	0.008219	0.041761
SLCO1B3	4.519785	4.512107	1.58898	2.83962	0.004517	0.028874
SLCO1B7	3.538358	5.187657	1.78759	2.90205	0.003707	0.025443
SLCO1C1	4.993912	5.218891	1.74742	2.98662	0.002821	0.02126
SLCO2A1	436.2977	1.632732	0.29157	5.59988	2.15E-08	2.63E-06
SLCO6A1	8.065893	2.197559	0.81967	2.68103	0.00734	0.03893
SLF1	23.29563	1.961629	0.62072	3.16025	0.001576	0.014544
SLFN11	9.796851	3.283385	0.83106	3.95084	7.79E-05	0.001843
SLFN13	6.175718	2.293031	0.84804	2.70393	0.006852	0.037364
SLFN5	23.67194	1.689209	0.53175	3.17668	0.00149	0.013978
SLIT2	12.54809	3.722744	0.87675	4.24608	2.18E-05	0.000684
SLIT3	6.327435	2.379741	0.78989	3.01276	0.002589	0.020084
SLITRK4	5.466177	4.225495	1.20373	3.51035	0.000448	0.006308
SLITRK5	4.217267	4.422211	1.56214	2.83086	0.004642	0.029375
SLITRK6	2.42085	4.063784	1.30023	3.12544	0.001775	0.0157
SMARCA1	222.5524	1.126142	0.21782	5.16997	2.34E-07	1.86E-05
SMC1B	7.95443	3.736976	1.35163	2.7648	0.005696	0.0334
SMCO4	12.0155	1.095734	0.42453	2.58108	0.009849	0.047249
SMIM21	4.839942	3.723252	1.45011	2.56757	0.010241	0.048569
SMIM22	24.10771	1.07217	0.31533	3.40019	0.000673	0.008248
SMKR1	34.87524	-1.75688	0.30587	-5.7438	9.26E-09	1.27E-06
SNAP91	7.468742	3.532022	0.97482	3.62326	0.000291	0.004674
SNCAIP	6.556985	3.029538	1.14573	2.64419	0.008189	0.041715
SNPH	3.054082	3.074125	1.17345	2.61974	0.0088	0.043949
SNTG1	8.744213	5.525398	1.44438	3.82543	0.000131	0.002661
SNTG2	2.279408	4.489336	1.47142	3.05102	0.002281	0.018536
SNX21	15.60031	1.122526	0.39592	2.8352	0.00458	0.029081
SNX30	12.02773	1.487349	0.50429	2.94939	0.003184	0.023114
SNX33	19.24201	2.01291	0.38708	5.20022	1.99E-07	1.66E-05
SOD1	1857.776	-1.54524	0.24023	-6.4324	1.26E-10	3.56E-08
SOD2	248.8448	1.005151	0.28486	3.52863	0.000418	0.006063
SORBS3	98.06734	1.148995	0.25357	4.53128	5.86E-06	0.000252
SORCS1	7.87082	4.45578	1.3963	3.19112	0.001417	0.013557
SORCS3	5.772023	4.432412	1.70048	2.60656	0.009146	0.04515
SORL1	23.05006	1.091852	0.39445	2.768	0.00564	0.033213
SOX11	4.637399	4.057039	1.52455	2.66113	0.007788	0.040484
SOX2-OT	5.023974	4.723225	1.4806	3.19008	0.001422	0.013575
SOX4	21.41448	1.523392	0.37812	4.0289	5.60E-05	0.001429
SOX5	17.87315	4.088369	0.95861	4.26488	2.00E-05	0.000642
SP140	10.44424	3.044626	1.01596	2.99678	0.002728	0.020758

SP6	28.33393	2.79987	0.43597	6.42215	1.34E-10	3.75E-08
SPAG17	8.402569	4.664684	1.38824	3.36014	0.000779	0.009093
SPAG4	13.35002	2.360848	0.56474	4.1804	2.91E-05	0.000855
SPATA22	3.622087	3.615163	1.22304	2.95589	0.003118	0.022756
SPATA6	9.462889	1.83584	0.68277	2.6888	0.007171	0.038322
SPHKAP	5.985412	4.942279	1.4661	3.37104	0.000749	0.008835
SPIN4	22.23598	1.307778	0.33418	3.91344	9.10E-05	0.002072
SPINK1	14.304	2.833367	0.98578	2.87425	0.00405	0.027033
SPN	6.448459	3.741082	1.04417	3.58283	0.00034	0.005228
SPON2	5.023195	2.569558	0.91301	2.81437	0.004887	0.030308
SPP1	45.69488	1.631194	0.41257	3.9537	7.70E-05	0.001843
SPRED1	12.53205	1.861171	0.5726	3.2504	0.001152	0.01174
SPTBN4	2.884342	4.344018	1.61664	2.68706	0.007208	0.038386
SQOR	10.61364	1.974894	0.70506	2.80104	0.005094	0.031111
SRD5A2	3.559187	4.657512	1.5521	3.00078	0.002693	0.020565
SRD5A3	3.725261	2.602713	0.94535	2.75317	0.005902	0.034087
SSPOP	3.94441	3.014602	1.17991	2.55494	0.010621	0.049574
ST3GAL5	5.091351	2.487257	0.7295	3.40954	0.000651	0.008127
ST3GAL6-AS1	15.34153	-1.18821	0.45083	-2.6356	0.008399	0.042404
ST6GAL1	12.07026	2.179062	0.63548	3.42898	0.000606	0.007719
ST6GAL2	8.627182	3.951907	1.02017	3.87379	0.000107	0.002301
ST6GALNAC5	3.882835	4.300991	1.54646	2.78118	0.005416	0.032449
ST8SIA3	2.656199	4.744659	1.71462	2.76719	0.005654	0.033271
ST8SIA4	53.09607	1.319365	0.40109	3.28948	0.001004	0.010683
ST8SIA6	9.316211	3.577206	0.82682	4.32645	1.52E-05	0.000526
STARD4-AS1	3.180049	3.986065	1.49344	2.66906	0.007606	0.039914
STC1	7.747157	3.138084	0.80774	3.88503	0.000102	0.002222
STC2	5.811875	4.435011	1.05984	4.18462	2.86E-05	0.000843
STEAP2	6.108649	4.531912	1.69493	2.6738	0.0075	0.039546
STK39	17.3362	1.786214	0.53474	3.34036	0.000837	0.00946
STOX2	8.800036	3.18418	1.2034	2.64598	0.008146	0.041582
STXBP5-AS1	6.943833	3.152854	0.94096	3.35066	0.000806	0.009263
STXBP5L	20.08508	4.910079	1.77146	2.77177	0.005575	0.03298
STXBP6	7.019469	2.028299	0.66495	3.0503	0.002286	0.018554
SUGCT	7.131414	2.591383	0.88401	2.93141	0.003374	0.02392
SUN2	80.58336	1.048491	0.2446	4.28661	1.81E-05	0.000599
SYBU	6.193945	1.868398	0.62392	2.9946	0.002748	0.020868
SYCP1	12.93795	4.355385	1.30025	3.34966	0.000809	0.009271
SYCP2	16.84006	3.561132	1.03804	3.43062	0.000602	0.007698
SYCP3	2.777975	4.775594	1.57784	3.02667	0.002473	0.019522
SYDE1	913.2293	1.250131	0.24047	5.19878	2.01E-07	1.66E-05
SYNE1	62.04018	2.187133	0.59977	3.6466	0.000266	0.004389
SYNPO2	23.84574	1.958985	0.54116	3.61996	0.000295	0.004702

SYNPO2L	74.68843	-1.05845	0.3535	-2.9942	0.002752	0.020875
SYT10	4.970839	4.67982	1.55341	3.0126	0.00259	0.020084
SYT14	10.19893	4.538523	1.24178	3.65484	0.000257	0.004302
SYT9	7.956042	5.391389	1.56858	3.43712	0.000588	0.00761
SYTL1	5.399775	2.573688	0.81767	3.1476	0.001646	0.014951
TAGAP	12.66318	1.765369	0.6625	2.6647	0.007706	0.040232
TAGLN	30.37345	1.883471	0.38713	4.86523	1.14E-06	6.82E-05
TARID	1.93893	4.275722	1.58551	2.69674	0.007002	0.037785
TBC1D16	8.169234	1.780467	0.61821	2.88006	0.003976	0.026672
TBC1D31	19.80284	1.47459	0.55918	2.63705	0.008363	0.042252
TBX18	6.389284	4.034972	1.44527	2.79184	0.005241	0.031687
TCEAL3	10.14927	2.687964	0.7222	3.72189	0.000198	0.003588
TCEAL7	4.245461	3.367589	1.08048	3.11676	0.001829	0.015945
TCERG1L	3.333432	3.066915	0.99328	3.08767	0.002017	0.017004
TCF4	42.4452	1.577267	0.4044	3.90028	9.61E-05	0.002151
TCF7L2	26.29876	1.323023	0.34262	3.86153	0.000113	0.002394
TCHH	613.8833	-1.08836	0.31061	-3.504	0.000458	0.006421
TCHHL1	9.964026	-1.14154	0.42416	-2.6913	0.007117	0.038158
TDRD1	4.796456	5.133585	1.37145	3.74317	0.000182	0.003408
TDRD15	6.553528	4.174274	1.47654	2.82707	0.004698	0.029558
TDRD5	5.996559	2.459425	0.89475	2.74874	0.005983	0.034396
TDRD9	8.334287	3.744372	1.33869	2.79704	0.005157	0.03138
TECRL	5.652783	4.867022	1.4933	3.25924	0.001117	0.011503
TENM1	13.16897	4.724849	1.30817	3.61179	0.000304	0.004815
TENM2	3.437596	3.193131	1.18829	2.68716	0.007206	0.038386
TENM4	7.998172	2.483579	0.67095	3.70157	0.000214	0.003803
TESPA1	10.57298	3.803052	1.21798	3.12243	0.001794	0.01582
TET1	14.00835	1.725292	0.34402	5.01509	5.30E-07	3.71E-05
TET3	339.5412	2.016385	0.31234	6.45569	1.08E-10	3.17E-08
TEX15	11.52078	5.111904	1.88818	2.70732	0.006783	0.037122
TG	7.139335	4.260266	1.22985	3.46405	0.000532	0.007086
TGFB2	8.467265	1.749427	0.58273	3.00214	0.002681	0.020541
TGIF2	10.68407	1.882292	0.66734	2.82058	0.004794	0.029934
TGM1	9.802744	2.636422	0.57774	4.56331	5.04E-06	0.000221
THBS4	3.59493	5.204089	1.79654	2.89674	0.003771	0.025776
THRB-AS2	183.069	1.84841	0.34949	5.28895	1.23E-07	1.13E-05
THSD7A	1190.169	-1.13189	0.22118	-5.1175	3.10E-07	2.38E-05
THSD7B	7.577819	5.303568	1.36077	3.89747	9.72E-05	0.002156
TIGIT	6.125418	4.469066	1.39058	3.21381	0.00131	0.012855
TIMP3	1470.625	1.556722	0.2876	5.41274	6.21E-08	6.43E-06
TIPIN	6.002866	3.581968	1.00335	3.57003	0.000357	0.005409
TK1	5.45603	2.120354	0.79914	2.65329	0.007971	0.041159
TLCD5	85.02701	-1.70755	0.26811	-6.3689	1.90E-10	4.76E-08
TLE6	8.412028	1.593976	0.46395	3.43567	0.000591	0.007633

TLR1	3.795195	2.332821	0.87021	2.68076	0.007345	0.038936
TLR7	3.925896	3.821311	1.29993	2.93963	0.003286	0.023511
TM4SF1	55.41369	1.783484	0.47334	3.76787	0.000165	0.003173
TM7SF3	15.5511	1.210599	0.4085	2.96351	0.003042	0.022394
TMC2	1.715891	4.1008	1.47744	2.77562	0.00551	0.032749
TMC6	28.06221	1.187929	0.42066	2.82393	0.004744	0.029715
TMED8	45.04206	1.130285	0.27498	4.11048	3.95E-05	0.001078
TMEM132B	12.20751	4.826708	1.02563	4.7061	2.52E-06	0.000126
TMEM168	100.5092	-1.22287	0.22378	-5.4645	4.64E-08	5.00E-06
TMEM178B	5.103914	3.837133	1.35443	2.83303	0.004611	0.029199
TMEM184A	19.76299	1.488431	0.39201	3.79692	0.000147	0.00291
TMEM196	5.448306	5.284378	1.46162	3.61543	0.0003	0.004766
TMEM200A	6.412969	3.483334	1.18246	2.94584	0.003221	0.023246
TMEM201	6.013324	1.471216	0.54114	2.71872	0.006554	0.036268
TMEM207	3.137582	3.981571	1.53297	2.5973	0.009396	0.045865
TMEM215	5.058591	4.675252	1.54458	3.02688	0.002471	0.019522
TMEM252	2.769152	4.361198	1.54995	2.81376	0.004897	0.030354
TMEM256	3.957686	2.350642	0.71888	3.26986	0.001076	0.011205
TMEM26	6.627293	3.43456	1.21594	2.82462	0.004734	0.029677
TMEM30A-DT	4.784045	3.487087	1.22775	2.84022	0.004508	0.028865
TMEM45A	74.89571	4.039097	0.53335	7.57303	3.65E-14	2.18E-11
TMEM67	9.068217	3.097855	0.85468	3.62457	0.000289	0.004671
TMEM74	6.029129	-1.944	0.72356	-2.6867	0.007216	0.038414
TMEM91	24.39353	2.485867	0.45839	5.42303	5.86E-08	6.13E-06
TMOD2	9.058722	2.071992	0.68636	3.01882	0.002538	0.019864
TMPRSS15	9.484571	3.84128	1.30279	2.9485	0.003193	0.023129
TMPRSS5	2.070638	4.381004	1.54396	2.83751	0.004547	0.028985
TMTC4	9.479017	1.523561	0.50033	3.04511	0.002326	0.018774
TMX4	203.4821	-1.00577	0.19638	-5.1215	3.03E-07	2.36E-05
TNC	7.314176	2.24876	0.84633	2.65707	0.007882	0.040846
TNFAIP2	148.9784	2.080332	0.33914	6.13409	8.56E-10	1.61E-07
TNFAIP6	4.113615	3.789141	1.46582	2.58499	0.009738	0.046884
TNFSF10	21.67359	2.205016	0.51657	4.26853	1.97E-05	0.000635
TNIK	16.36901	1.956376	0.65008	3.00946	0.002617	0.020187
TNIP3	3.662125	4.230184	1.58272	2.67272	0.007524	0.039621
TNNT1	29.48085	2.109257	0.40054	5.26599	1.39E-07	1.25E-05
TNNT2	2.401683	3.485436	1.19565	2.9151	0.003556	0.024757
TNNT3	6.256875	2.803499	0.68908	4.06847	4.73E-05	0.001246
TNR	8.133307	2.989232	0.70787	4.22286	2.41E-05	0.000746
TNS2	11.08142	1.101008	0.43036	2.55835	0.010517	0.049364
TONSL	4.036547	2.164718	0.75639	2.8619	0.004211	0.027709
TOP2A	19.34565	1.691051	0.49075	3.44584	0.000569	0.007452
TOX3	2.69154	4.772737	1.74371	2.73711	0.006198	0.03516
TPBG	57.28434	2.227214	0.29399	7.57584	3.57E-14	2.18E-11

TPH2	2.806018	4.241762	1.40268	3.02404	0.002494	0.019642
TPM2	12.21586	2.03861	0.50062	4.07219	4.66E-05	0.001228
TPPP	12.74851	5.272853	1.14145	4.61944	3.85E-06	0.000177
TPRN	8.273409	1.309741	0.4977	2.63158	0.008499	0.042806
TRAF1	4.268492	2.778838	1.08663	2.55729	0.010549	0.04942
TRDC	7.493582	2.494016	0.78697	3.16916	0.001529	0.014213
TRDN-AS1	3.660824	4.21204	1.64576	2.55932	0.010488	0.049272
TREM1	100.7233	3.094265	0.50222	6.16117	7.22E-10	1.37E-07
TREML2	758.5769	1.097833	0.24283	4.52091	6.16E-06	0.000263
TRERF1	6.977604	3.238722	1.03892	3.11739	0.001825	0.015937
TRERNA1	2.35126	3.413825	1.24297	2.74651	0.006023	0.034514
TRHDE	8.663564	4.32108	1.27147	3.3985	0.000678	0.00828
TRHDE-AS1	6.371962	4.478488	1.45516	3.07766	0.002086	0.017395
TRIM14	38.67764	2.507889	0.56753	4.41896	9.92E-06	0.000381
TRIM16	8.195706	1.446382	0.50591	2.85897	0.00425	0.027841
TRIM36	6.176547	3.146892	1.14684	2.74397	0.00607	0.034702
TRIM51	4.464185	5.023916	1.85297	2.71128	0.006702	0.036831
TRIM65	4.875716	2.249905	0.7669	2.93376	0.003349	0.023842
TRIM72	2.075969	3.232419	1.24873	2.58857	0.009638	0.046567
TRIP6	13.66815	1.739396	0.41538	4.18747	2.82E-05	0.000834
TRMT9B	97.79052	-1.19378	0.31324	-3.811	0.000138	0.002786
TRNC	24.65186	1.435614	0.52685	2.72491	0.006432	0.035906
TRND	11.02273	2.99393	0.93947	3.18681	0.001438	0.01368
TRNE	295.3183	2.682314	0.77327	3.4688	0.000523	0.007015
TRNF	47.98489	1.795118	0.52631	3.41078	0.000648	0.008116
TRNG	32.29572	2.349226	0.62472	3.76042	0.00017	0.003241
TRNH	13.78865	2.522653	0.67866	3.71712	0.000202	0.003637
TRNI	65.61125	1.947311	0.3916	4.97265	6.60E-07	4.31E-05
TRNK	16.3867	3.339875	0.84367	3.95875	7.53E-05	0.00181
TRNL1	98.5206	2.19287	0.50626	4.33155	1.48E-05	0.000517
TRNL2	33.99496	1.92085	0.57847	3.32058	0.000898	0.009912
TRNM	63.11569	2.251802	0.50942	4.4203	9.86E-06	0.000381
TRNP	345.308	2.643609	0.55865	4.73212	2.22E-06	0.000115
TRNQ	307.7755	2.731826	0.83222	3.28259	0.001029	0.010847
TRNS1	158.9828	2.86964	0.75128	3.81964	0.000134	0.002708
TRNS2	2.847012	3.244431	1.18745	2.73226	0.00629	0.035532
TRNT	77.77654	2.384397	0.38966	6.11921	9.40E-10	1.75E-07
TRNV	41.97457	1.719129	0.4775	3.60026	0.000318	0.00498
TRNW	425.8926	2.33262	0.36559	6.38047	1.77E-10	4.60E-08
TRNY	18.85171	1.69198	0.53913	3.13836	0.001699	0.015259
TRPA1	6.022499	4.973055	1.59836	3.11135	0.001862	0.016135
TRPC1	7.744261	3.840849	1.14861	3.34391	0.000826	0.009418
TRPM3	7.005402	4.749011	1.3826	3.43484	0.000593	0.007647
TRPM8	5.527025	5.341807	1.69037	3.16013	0.001577	0.014544

TSC22D3	39.45356	1.327847	0.37908	3.50282	0.00046	0.006438
TSG1	6.10728	4.977215	1.3338	3.73161	0.00019	0.003496
TSHZ2	11.67146	3.173717	0.89361	3.55157	0.000383	0.005722
TSHZ3	3.967714	3.195106	1.18293	2.701	0.006913	0.037572
TSIX	20.8567	5.440009	1.35183	4.02419	5.72E-05	0.001449
TTC26	7.621625	3.506507	1.20357	2.91342	0.003575	0.024859
TTC6	5.66383	4.859127	1.29884	3.74114	0.000183	0.003426
TTK	11.49078	3.244511	0.81637	3.97429	7.06E-05	0.001724
TTN	81.04829	4.019663	0.97341	4.12947	3.64E-05	0.001022
TTYH3	12.84219	1.326051	0.44794	2.96036	0.003073	0.02255
TUBA4A	120.2566	1.129368	0.27616	4.08948	4.32E-05	0.001171
TUBB6	22.7073	1.193764	0.39929	2.98975	0.002792	0.021074
TVP23A	10.06327	3.281695	0.82971	3.95521	7.65E-05	0.001834
TXLNB	7.616359	3.498611	1.31337	2.66384	0.007725	0.040286
TXNIP	106.214	1.220297	0.38127	3.20063	0.001371	0.013283
TXNRD1	1497.486	-1.2758	0.23336	-5.467	4.58E-08	4.97E-06
TYRP1	3.339523	5.092152	1.79278	2.84036	0.004506	0.028864
U2AF1	9.741203	-5.69277	1.92202	-2.9619	0.003058	0.022481
UACA	24.04263	1.698101	0.58774	2.88922	0.003862	0.026185
UBASH3A	2.984755	3.366584	1.23471	2.72661	0.006399	0.035833
UBASH3B	65.9326	1.85799	0.29954	6.20276	5.55E-10	1.07E-07
UBE2L6	34.78424	1.976274	0.56265	3.51242	0.000444	0.006271
UCA1	1093.435	1.848242	0.35601	5.19156	2.09E-07	1.71E-05
UGT2B11	5.393774	4.308688	1.57674	2.73265	0.006283	0.035515
UGT3A1	3.535915	5.17062	1.58514	3.26194	0.001107	0.011446
ULK3	18.38124	1.188134	0.377	3.15158	0.001624	0.01481
UMOD	3.626049	5.198875	1.5741	3.30275	0.000957	0.010319
UMODL1	1.914623	3.679622	1.4187	2.59366	0.009496	0.046117
UNC13A	6.303555	4.497329	1.34332	3.34791	0.000814	0.009323
UNC13B	30.78648	1.045761	0.28143	3.71593	0.000202	0.003646
UNC13C	11.34945	5.088645	1.37003	3.71425	0.000204	0.003665
UNC5C	9.004367	4.672301	1.30507	3.58012	0.000343	0.005263
UNC5D	10.41243	4.048413	1.44233	2.80685	0.005003	0.030788
UNC80	17.85057	4.42387	1.64934	2.6822	0.007314	0.038832
UPK2	5.300159	4.755119	1.04449	4.55258	5.30E-06	0.000229
URB1	24.34042	1.146465	0.39992	2.86671	0.004148	0.027414
USH2A	22.17299	2.245549	0.78575	2.85784	0.004265	0.027862
USP18	8.840701	3.79703	0.89988	4.21948	2.45E-05	0.000748
USP28	12.68491	1.977332	0.64388	3.07095	0.002134	0.017686
USP32P1	2.796225	2.890882	0.99746	2.89824	0.003753	0.025664
USP44	4.394946	5.519822	1.73459	3.18221	0.001462	0.013794
USP46-DT	45.20206	-1.2075	0.21798	-5.5396	3.03E-08	3.45E-06
USP6	4.211189	3.963366	1.38724	2.85702	0.004276	0.027879
VANGL1	6.427238	2.623192	0.75495	3.47468	0.000511	0.006931

VANGL2	6.099385	3.128574	1.22167	2.5609	0.01044	0.049166
VASH1	14.48258	1.231526	0.44828	2.74725	0.00601	0.034488
VCAM1	4.379422	3.143854	1.19211	2.63721	0.008359	0.042245
VCAN-AS1	5.388291	5.274221	1.35395	3.89543	9.80E-05	0.002156
VDR	66.2172	1.402827	0.30417	4.61203	3.99E-06	0.000182
VEGFA	6.446396	1.768349	0.55815	3.16823	0.001534	0.014234
VEGFC	2.673746	3.174414	1.11304	2.85203	0.004344	0.028149
VIM	202.3211	1.126397	0.36258	3.10662	0.001892	0.016309
VIP	4.236693	4.433759	1.71815	2.58054	0.009864	0.047267
VIT	3.831751	4.279112	1.29485	3.30471	0.000951	0.010278
VNN3P	35.49314	-1.92381	0.42488	-4.5279	5.96E-06	0.000255
VOPP1	10.01628	2.202255	0.69117	3.18628	0.001441	0.013688
VSTM2A	5.093478	3.647532	1.29258	2.82191	0.004774	0.029857
VWA1	123.9823	1.184355	0.22033	5.37536	7.64E-08	7.67E-06
VWA2	2.146701	4.487097	1.66208	2.69969	0.00694	0.037628
VWA3A	5.908137	4.418923	1.55548	2.84088	0.004499	0.028828
VWA5B1	4.516984	5.557355	1.37807	4.0327	5.51E-05	0.001411
VWC2L	4.238701	5.460334	1.54783	3.52773	0.000419	0.006063
VWCE	18.7533	2.238891	0.43926	5.09699	3.45E-07	2.59E-05
VWDE	21.08863	2.767376	0.64007	4.32354	1.54E-05	0.000526
WASF3	7.528473	2.282953	0.77863	2.93201	0.003368	0.023911
WDFY4	16.67285	2.439863	0.72372	3.37126	0.000748	0.008835
WDR17	14.31635	5.718914	1.6681	3.42839	0.000607	0.007729
WDR31	6.206411	2.74835	1.01972	2.6952	0.007035	0.037887
WDR72	11.6732	2.409884	0.79801	3.01987	0.002529	0.019847
WDR76	10.80985	1.544085	0.43929	3.51498	0.00044	0.006248
WDR86	21.0606	2.459476	0.49373	4.98142	6.31E-07	4.18E-05
WDR86-AS1	95.73816	2.163935	0.45095	4.79856	1.60E-06	8.82E-05
WDR87	2.718732	4.312071	1.62111	2.65995	0.007815	0.040613
WHRN	5.126192	2.988356	0.89424	3.34179	0.000832	0.00945
WIF1	3.870227	5.308101	1.81612	2.92277	0.003469	0.024302
WIPF1	87.56659	1.01113	0.30941	3.26794	0.001083	0.011246
XAF1	13.02695	1.830075	0.57747	3.16915	0.001529	0.014213
XIRP2	11.45875	4.483655	1.37145	3.26927	0.001078	0.011205
XKR3	1.554306	3.997244	1.46617	2.72632	0.006405	0.03584
YEATS2	30.91092	1.119954	0.34207	3.27403	0.00106	0.011066
ZAN	4.267741	4.456282	1.62314	2.74547	0.006042	0.03458
ZBBX	6.95216	3.401391	1.17846	2.88631	0.003898	0.026301
ZBED2	4.687047	3.557285	1.28969	2.75825	0.005811	0.03384
ZBTB16	3.238493	4.512546	1.31708	3.42618	0.000612	0.007768
ZBTB4	192.3745	1.11979	0.1609	6.95963	3.41E-12	1.38E-09
ZBTB7C	12.49365	1.37831	0.49206	2.80111	0.005093	0.031111
ZC2HC1A	77.57462	-1.53043	0.37533	-4.0776	4.55E-05	0.001214
ZC3H12B	4.860497	3.76009	1.25266	3.00169	0.002685	0.020542

ZDHHC14	12.70505	1.193217	0.40231	2.96594	0.003018	0.022297
ZEB1	46.1577	1.511824	0.38297	3.94762	7.89E-05	0.00186
ZEB2	33.40612	1.604759	0.48237	3.32682	0.000878	0.009747
ZFHX2	3.570478	4.151823	1.47041	2.82357	0.004749	0.029737
ZFHX4	12.99436	6.142127	1.77633	3.45777	0.000545	0.007216
ZFP42	2.554314	3.220707	1.14673	2.8086	0.004976	0.03068
ZFPM2	5.918371	2.83763	0.94818	2.99271	0.002765	0.020914
ZFTA	17.30527	1.050671	0.3973	2.64452	0.008181	0.041715
ZMAT1	8.86364	5.545842	1.30037	4.26481	2.00E-05	0.000642
ZNF114	62.03662	1.564353	0.42903	3.64622	0.000266	0.004389
ZNF204P	6.718038	2.397638	0.73099	3.28	0.001038	0.010919
ZNF215	13.96188	-2.22299	0.76001	-2.925	0.003445	0.024189
ZNF219	15.23847	1.425083	0.41755	3.413	0.000643	0.008075
ZNF300	3.100734	4.976908	1.43001	3.48032	0.000501	0.00685
ZNF300P1	4.589267	2.670468	0.84277	3.16867	0.001531	0.014221
ZNF358	15.85331	1.333223	0.47641	2.79848	0.005134	0.031288
ZNF365	7.044954	3.218113	1.03102	3.1213	0.001801	0.015825
ZNF367	8.307699	2.504655	0.7322	3.42074	0.000625	0.0079
ZNF385B	7.564956	5.327814	1.69868	3.13644	0.00171	0.01529
ZNF385D	18.50571	3.285068	1.05124	3.12494	0.001778	0.015711
ZNF441	27.80043	-1.06031	0.31691	-3.3458	0.00082	0.009381
ZNF483	11.97846	3.267335	0.86663	3.77015	0.000163	0.003155
ZNF516	6.901921	1.988389	0.63203	3.14602	0.001655	0.014982
ZNF519	10.57113	3.608263	1.2306	2.93211	0.003367	0.023911
ZNF521	6.599246	2.253583	0.7795	2.89105	0.00384	0.026147
ZNF536	4.051518	4.856741	1.52667	3.18126	0.001466	0.013815
ZNF554	427.3951	-1.03788	0.23269	-4.4603	8.19E-06	0.000329
ZNF555	27.60651	-1.04932	0.3144	-3.3376	0.000845	0.009513
ZNF648	2.147273	3.847311	1.45839	2.63805	0.008339	0.042194
ZNF696	2.875348	2.992243	0.96597	3.09765	0.001951	0.016624
ZNF702P	249.1017	-1.16478	0.21022	-5.5408	3.01E-08	3.45E-06
ZNF704	52.86703	1.195651	0.35309	3.38622	0.000709	0.008492
ZNF711	3.35179	3.45604	1.30528	2.64774	0.008103	0.041517
ZNF716	4.376386	4.491205	1.74107	2.57957	0.009892	0.047358
ZNF726	5.071979	2.409417	0.92651	2.60053	0.009308	0.045615
ZNF775	2.161936	2.852349	1.05973	2.69158	0.007111	0.038158
ZNF826P	1.642412	3.590432	1.16341	3.08613	0.002028	0.017021
ZNF831	10.96967	2.235503	0.79949	2.79617	0.005171	0.03143
ZNF852	13.12104	-1.0705	0.37049	-2.8894	0.00386	0.026185
ZNF99	5.297598	5.285307	1.4904	3.54624	0.000391	0.005772
ZPBP	4.243928	4.932593	1.74756	2.82255	0.004764	0.029808
ZSWIM2	5.545653	2.030041	0.78008	2.60234	0.009259	0.045472
ZSWIM6	7.671049	1.868565	0.68748	2.71798	0.006568	0.036312
ZSWIM9	20.70031	1.210481	0.39681	3.05052	0.002284	0.01855

ZWINT	6.942726	2.366267	0.89628	2.6401	0.008288	0.042031
